# Elucidation of α-glucosidase inhibitory activity and UHPLC-ESI-QTOF-MS based metabolic profiling of endophytic fungi *Alternaria alternata* BRN05 isolated from seeds of *Swietenia macrophylla* king

**DOI:** 10.3389/ffunb.2025.1447609

**Published:** 2025-01-28

**Authors:** Piyush Kumar, Sai Anand Kannakazhi Kantari, Ranendra Pratap Biswal, Prasanth Ghanta, Malleswara Dharanikota

**Affiliations:** ^1^ Department of Biosciences, Sri Sathya Sai Institute of Higher Learning, Puttaparthi, Andhra Pradesh, India; ^2^ Research Executive Karkinos Healthcare, Navi Mumbai, Maharashtra, India

**Keywords:** α-glucosidase inhibitors, *Alternaria alternata*, full-strength media (FS), quarter-strength media (QS), diabetes, ultra-high-performance liquid chromatography - electrospray ionization - quadrupole time-of-flight mass spectrometry (UHPLC-ESI-QTOF-MS), molecular dynamics (MD) simulation

## Abstract

There is a growing demand for new diabetes drugs with fewer side effects to replace current medications known for their adverse effects. Inhibition of α-glucosidase responsible for postprandial hyperglycemia among diabetes patients is a promising strategy for managing the disease. This study aims to explore and identify novel bioactive metabolites with anti-diabetes potential from *Alternaria alternata* BRN05, an endophytic fungus isolated from a well-known medicinal plant *Swietenia macrophylla* King. Ethyl acetate extracts of *Alternaria alternata* BRN05 grown in full-strength (EFS) and quarter-strength (EQS) media, respectively were evaluated for their α-glucosidase inhibitory activities. Based on IC_50_ values, EQS exhibited significantly greater inhibitory activity (0.01482 ± 1.809 mg/mL) as compared to EFS (1.16 ± 0.173 mg/mL) as well as acarbose control (0.494 ± 0.009 mg/mL). EFS and EQS were subjected to metabolic profiling using Ultra-High-Performance Liquid Chromatography - Electrospray Ionization - Quadrupole Time-of-Flight Mass Spectrometry (UHPLC-ESI-QTOF-MS). A total of nineteen metabolites from EFS and twenty from EQS were tentatively identified based on MS/MS fragmentation. Molecular docking analysis revealed that twelve among these exhibited greater binding energies than that of acarbose (-6.6 kcal/mol). Molecular Dynamics (MD) simulations of 3’,4’,7-trihydroxyisoflavanone (THF) and alternariol 9-methyl ether (AME) from EQS, exhibiting high binding energies (-7.5 and -7 kcal/mol, respectively), were performed to investigate their interactions with human intestinal α-glucosidase. Results suggest THF possesses strong inhibitory potential, making it a promising candidate for diabetes management.

## 1 Introduction

Diabetes has become a pandemic, with the number of cases rising from 200 million in 1990 to 830 million in 2022, affecting more people in low- and middle-income countries (Diabetes, 2024). Currently, 10.5% of adults (20-79 years) have diabetes, with nearly half unaware of their condition. Over 90% of them have type 2 diabetes, driven by factors like urbanization, aging populations, reduced physical activity, and rising obesity rates. By 2045, diabetes is projected to affect 1 in 8 adults (783 million), a 46% increase (IDF Diabetes Atlas, n.d.). Diabetes management has seen significant advancements in recent years, with a range of medications now available to control blood sugar levels. These include metformin, GLP-1 agonists, SGLT-2 inhibitors, DPP-4 inhibitors, and dual GLP-1/GIP receptor agonists. However, these treatments often come with serious side effects and may not be suitable for all patients due to individual health variations (Vesa and Bungau, 2023). The enzyme α-glucosidase, found in the small intestine, is crucial in breaking down complex carbohydrates and contributes to elevated blood sugar levels in diabetic patients. Alpha-glucosidase inhibitors (AGIs) effectively slow down the breakdown and absorption of carbohydrates, preventing sudden spikes in blood glucose levels associated with serious complications such as cardiovascular disease (Dirir et al., 2022).

AGIs are generally considered to have fewer side effects compared to other oral diabetes drugs, as they work primarily in the intestines rather than affecting the body’s overall metabolism (Hussain et al., 2021). While commercial α-glucosidase inhibitors like acarbose, voglibose, and miglitol are widely used to manage postprandial hyperglycemia, they too can cause adverse effects such as diarrhea and bloating. This situation highlights the need for alternative treatments that effectively inhibit α-glucosidase with minimal side effects. Natural sources, particularly endophytic fungi, have emerged as a promising avenue for developing novel α-glucosidase inhibitors (Shao et al., 2023).

Since many genes encoding secondary metabolites in endophytic fungi are usually silent or cryptic under normal culture conditions, there is a need to activate them. Genes responsible for producing secondary metabolites normally occur in groups referred to as Biosynthetic Gene Clusters (BGCs). A fungal genome usually contains about 40-50 BGCs. Activation of BGCs can lead to the production of a set of novel compounds (Keller, 2019). Several methods can be employed for activating BGCs including co-culturing with microbes like bacteria and fungi; addition of small-molecule elicitors such as DNA methyltransferase inhibitors (e.g., 5-azacytidine) or histone deacetylase inhibitors (e.g., SAHA); addition of physical scaffolds like cotton scaffold and microparticles to the culture media; changing physical or chemical conditions such as temperature, light, salt concentration; and altering media concentration (Tomm et al., 2019). Analysis of available databases using antiSMASH software showed that *Alternaria* harbors 22 BGCs involved in the production of secondary metabolites. Out of these BGCs, only eight have been studied to some extent, while information for the remaining fourteen is not available (Tao et al., 2022). Exploring their fungal metabolites can lead to the production of new molecules which can be screened for various diseases, including diabetes.

Some well-known drugs derived from fungus include cephalosporins, lovastatin, cyclosporin, ergotamine, and myriocin (Nazir et al., 2021). Metabolites isolated for anti-diabetic activity from fungi include fumosorinone A from *Isaria fumosorosea*, which inhibited PTP1B and enhanced insulin signaling (Liu et al., 2015). Encindolenes D-H (compounds 1-5) from *Penicillium* sp. HFF16 inhibit hepatic glucose production with EC50 values ranging from 9.6 to 30.1 μM (Xiao et al., 2022). Thielavin K, derived from the MEXU 27095, has exhibited *in vivo* antihyperglycemic properties in mice (Rivera-Chávez et al., 2013).

Genus *Alternaria*, belonging to the family Pleosporaceae, is a fungus distributed widely across the world. In the last twenty years, at least 268 metabolites exhibiting good bioactivity have been reported from different species of *Alternaria* (Sabreen et al., 2015). Bioactive metabolites with diverse medicinal uses are reported from endophytic species of *Alternaria*, such as saponins, phenols, steroids, and glycosides possessing antioxidant properties; taxol, coumarins, paclitaxel, and camptothecin showing anti-tumor properties; and alternariol, alterchromone A, tannins, and alternariol-9-methyl ether exhibiting antimicrobial properties (Eram et al., 2018). Porritoxin is a well-known chemopreventive molecule with potential for use as a drug in the treatment of cancer (Kim et al., 2009).

*Swietenia macrophylla* King, belonging to the family Meliaceae, is a medicinal plant traditionally used for treating various ailments. In India, leaf and bark decoctions traditionally treat nerve ailments and diarrhea, while fruit extracts address hypertension and skin problems. In Malaysia and Indonesia, raw or crushed *Swietenia* seeds are used for hypertension, malaria, and diabetes treatment. Species of the genus *Swietenia* are rich in phytochemicals like limonoids, and polyphenolics known to exhibit medicinal properties. Bioactive metabolites such as swietemacrophia and swietenolide possess good anti-inflammatory and antibacterial activities (Da Silva et al., 2008). During the last decade, *Swietenia* has gained prominence in diabetes treatment (Sukardiman and Ervina, 2020). In this study, we explore the potential of endophytic fungi *Alternaria* isolated from this host as a potential antidiabetic agent.

## 2 Materials and methods

### 2.1 Chemicals and reagents

We purchased α-glucosidase derived from *Saccharomyces cerevisiae* (CAS No: 9001-42-7) from Merck Saint Louis, USA, PNPG (p-nitrophenyl-alpha-D-glucopyranoside) LOT: 3533550 was obtained from Merck Darmstadt, Germany, and Acarbose (CAS No: 56180-94-0, purity >98%) was sourced from TCI, Tokyo, Japan. Potato dextrose agar (PDA, GMO96-500G), Potato dextrose broth (PDB, GM403-G00G), Folin & Ciocalteu’s Phenol reagent Hi-LR™ (RM10822-100ML), Ethyl acetate (CAS No: 141-78-6, purity 99.9%), and Methanol (CAS No: 67-56-1, purity 99.8%) of HPLC grade used for extraction were purchased from Himedia, Mumbai, India. LC-MS grade Acetonitrile (CAS No: 75-05-8, >99.9% purity) was procured from Fisher Scientific, Fair Lawn, NJ, USA. Milli Q: Type 1 water, purified using the Merck Milli Q IQ7000 water purification system.

### 2.2 Collection of samples

Seeds were collected from *Swietenia macrophylla* King located at the geographical coordinates (Latitude – 13.001549˚ and Longitude - 77.758875˚) at Sri Sathya Sai Institute of Higher Learning, Brindavan campus, Whitefield, Kadugodi, Bengaluru, Karnataka-560067. L. Rasingam from the Botanical Survey of India (Deccan Regional Centre), Ministry of Environment, Forests & Climatic Change, Hyderabad, Telangana, India, authenticated the plant sample. The voucher number for the sample is BSI/DRC/2022-23/Identification/94.

### 2.3 Isolation of endophytic fungi

The mature fruits of *Swietenia macrophylla* King yielded approximately 35 to 40 seeds. The seeds were washed with distilled water and then cut into small pieces measuring 0.5 cm². To sterilize the seed surfaces, around 300 seed pieces were placed in a beaker and treated with 70% ethanol (50 mL) for 10 seconds, followed by a 3-minute treatment with 4% sodium hypochlorite (50 mL). After one minute, the seeds were rinsed with sterile distilled water (50 mL) (Ramdanis et al., 2012). Next, five sterilized seed pieces were placed on 9 mm petri plates containing 20 mL of Potato Dextrose Agar (PDA) media. The plated seeds were then incubated for 3-6 days until colonies of endophytic fungi became visible (refer to **Supplementary File S1**). Fungal growth from the seeds was transferred using a loop into fresh petri plates containing PDA media to obtain pure cultures. Glycerol stocks of the resulting pure fungal cultures were preserved at -80°C for future use.

### 2.4 Extracellular metabolites extraction

The pure isolates of endophytic fungi were inoculated into 100 mL full strength (FS) and Quarter strength (QS) PDB media and was cultured for 21 days in static condition at 25°C (Ranganathan and Mahalingam, 2019). Before extraction, the fungal culture was homogenized with 10% methanol. Ethyl acetate was used as the solvent for extraction in a 1:1 ratio, and the process was repeated twice to extract maximum metabolites from the culture. Ethyl acetate was evaporated using a rotary evaporator, and the resulting extract was dried and stored at -20°C for further analysis (Kantari et al., 2023).

### 2.5 Phylogenetic analysis of endophytic fungi using ITS-rDNA fragment

The isolated endophytic fungi were investigated by first extracting genomic DNA from their mycelium using a phenol-chloroform extraction method. Next, PCR was carried out by using specific primers (ITS1 forward primer and ITS4 reverse primer) to amplify the ITS-rDNA fragment (Raja et al., 2017). The resulting sequences were then compared to other sequences in the NCBI GenBank using BLAST searches. To determine the relationships between different fungal species, phylogenetic analysis was conducted using MEGA X (Kumar et al., 2018). The evolutionary distances were calculated using the maximum composite likelihood technique. The resulting tree was created using the Neighbour-Joining method. To ensure the accuracy of the tree, a process called bootstrapping was used (Tamura et al., 2004). The tree was tested 1000 times to determine the frequency with which the species grouped together. This allows us to confidently infer the evolutionary history of endophytic fungi.

### 2.6 Total phenolic content

The total phenolic content (TPC) was determined with slight modifications from Akhtar et al. (2018) using Folin-Ciocalteu reagents (FC reagents). Gallic acid served as the reference for constructing the standard curve. It was dissolved in methanol at concentrations ranging from 1 µg/mL to 10 µg/mL to prepare the standards. Samples of EFS and EQS were prepared at a concentration of 1 mg/mL in methanol. Thirty microliters (30 µL) of each standard solution and samples were added to the 96-well plate, followed by the addition of 10X-diluted Folin reagent (150 µL). Subsequently, 120 µL of 5% sodium carbonate solutions was added and plate was incubated in the dark at room temperature for thirty minutes. After incubation, the absorbance was measured at 765 nm using a microplate reader. The results of the TPC analysis were expressed in gallic acid equivalents per gram of dry extract weight (mg GAE/g).

### 2.7 Antidiabetic assays

The α-glucosidase inhibition assay was optimized by incorporating modification from Bhatia et al. (2019). Ethyl acetate (EtOAc) extracts from full strength (FS) and quarter strength (QS) PDB cultures of the nine endophytic fungi isolated from *Swietenia macrophylla* King seeds were screened for inhibitory activity of α-glucosidase. First, a 0.1 M phosphate buffer at pH 6.9 was prepared. Subsequently, α-glucosidase was dissolved in the phosphate buffer to achieve a concentration of 0.2 U/mL. Subsequently, a solution of 1 mM PNPG (p-nitrophenyl-alpha-D-glucopyranoside) was prepared in the phosphate buffer. Samples of EFS and EQS were initially prepared at concentrations of 2 mg/mL and 1 mg/mL, respectively, by dissolving them in a mixture of 10% dimethyl sulfoxide (DMSO) and phosphate buffer. Following favorable outcomes, these samples were prepared at various concentrations for IC_50_ calculation. Additionally, a 0.1 M Na_2_CO_3_ solution was prepared using MilliQ water. Acarbose, serving as the positive control, was prepared in the phosphate buffer. Moreover, 10% DMSO was utilized as the vehicle control for the experiment. In the 96-well plate, 50 µL of 0.1 M phosphate buffer at pH 6.9 was dispensed. Subsequently, 10 µL of α-glucosidase was added. Following this, 20 µL of extracts were introduced at various concentrations, ranging from 0.6 mg/mL to 4 mg/mL for FS and from 5 µg/mL to 25 µg/mL for QS. Then, 20 µL of PNPG was added, and the incubation was continued for another 20 minutes at 37°C. The reaction was terminated by the addition of 100 µL of Na_2_CO_3_. Finally, the absorbance of the reaction mixture was measured at 405 nm using a microplate reader (SpectraMax^®^ M2e). Percentage inhibition was calculated using the following equation:


Percentage inhibition = 1−[As/Ac] * 100

whereas As signifies the absorbance of the sample and Ac signifies the absorbance of the control. The IC_50_ value was calculated for the endophytic fungal strain in both EFS and EQS.

### 2.8 Metabolite profiling of UHPLC-ESI-QTOF-MS

The EFS and EQS was subjected to metabolite profiling using UHPLC-ESI-QTOF-MS mass spectrometry purchased from Agilent model 1290 that is coupled to Agilent 6550 Q-TOF LC-MS with dual jet stream ionization source in negative mode. Agilent MassHunter version B.05.00 software (Agilent Technologies, USA) was employed for data acquisition.

Samples EFS and EQS were prepared by dissolving in methanol to achieve a final concentration of 0.1 mg/mL. Filtration of EFS and EQS was performed using an Agilent Econofilter column composed of polytetrafluoroethylene (PTFE) with a 13 mm diameter and a 0.22 µM pore size. Metabolite separation was carried out on an Agilent ZORBAX RRHD Eclipse Plus C18 column (3.0×100 mm, 1.8 µm) utilizing a gradient elution profile with mobile phase A (0.1% HCOOH in water) and mobile phase B (0.1% HCOOH in acetonitrile). The elution gradient consisted of the following time intervals: 0-2 min at 5% B, 2-21 min with a gradual transition from 5% to 20% B, 21-40 min with a progressive transition from 20% to 50% B, 40-45 min with a stepwise transition from 50% to 95% B, 45-49 min at a constant 95% B, and 49-51 min returning to 5% B. The injection volume was 10 µL, and the flow rate was maintained at 0.300 mL/min (Mohammed et al., 2021).

For the quadrupole time of flight analysis, specific parameters were set as follows: nozzle voltage of 1000 V, capillary voltage of 3.5 kV, nebulizer pressure of 35 psi, and drying gas nitrogen flow rates of 11 l/min with respective temperatures of 250°C and 350°C. Mass calibration was performed using the G1969-85,000 reference standard mix (Supelco, Inc.) in both positive and negative ionization modes, resulting in a minimal residual error value of 0.2 ppm (Biswal et al., 2022).

### 2.9 Library creation for metabolites database

A collection of metabolites derived from *Alternaria* sp., including known α-glucosidase inhibitors and well-known polyphenols, was gathered from existing literature. A comprehensive database comprising 850 small compounds was created using the Agilent Personal Compound Database and Library (PCDL) software (Agilent Technologies, USA). The software is used to manage the content of personal compound databases and libraries and is part of the Agilent MassHunter Workstation software suite. The structures of these metabolites were either drawn using ChemDraw software (ChemBioOffice Suite, PerkinElmer Informatics, USA) or retrieved from the PubChem Database.

Chromatograms of EFS and EQS extracts were analyzed using Agilent MassHunter Qualitative Analysis software (B.7.00). The uploaded data was screened using the “Find by formula” option, which enabled analysis within specified mass windows. Metabolite identification was conducted through precise mass measurement of precursor ions, comparing the experimental and theoretical isotopic patterns with those stored in the PCDL manager file, which contains a list of 850 metabolites.

Metabolites with an overall identification score of over 80% for the isotopic pattern were considered for further analysis. This score was calculated considering exact masses, relative abundance, and spacing, with a mass error of less than 5 ppm.

### 2.10 Molecular docking

Human Maltase-glucoamylase (MGAM) plays a crucial role in the final breakdown of starch into glucose within the small intestine. This enzyme is categorized under Glycosyl hydrolase family 31 (GH31) due to its α-glucosidase activity. MGAM comprises two subunits, NtMGAM located in the N-terminal region, and CtMGAM located in the C-terminal region. The primary function of human MGAM is to cleave α (1-4) linkages between glucosyl units, ultimately producing glucose as the final product. The goal is to assess the ligand’s inhibition capacity against NtMGAM through *in-silico* studies. For our docking studies, we focused on NtMGAM since it prefers cleaving α-1-4-bonds of shorter oligosaccharides and is involved in the final step of glucose formation. CtMGAM on the other hand prefers cleaving longer oligosaccharides (Brás et al., 2018; Elferink et al., 2020). Since standard drug acarbose weakly inhibits NtMGAM compared to CtMGAM, there is need for new drugs which target the latter (Laoud et al., 2018). NtMGAM is therefore selected as the representative of GH31 α-glucosidase family to which yeast α-glucosidase belongs to Kashtoh and Baek (2022). In the C-terminal region of NtMGAM, there exists a (β/α)8-barrel structure, which primarily constitutes the active site of the protein. Notable among these residues are catalytic nucleophiles like Asp443 and Asp542, which are shared across GH31 family members and play a vital role in acid-base catalysis. Additionally, residues such as His600, Asp327, and Arg526 contribute to hydrogen bonding and are also highly conserved in the GH31 family of α-glucosidase (Sim et al., 2008).

To analyze the docking studies of α-glucosidase in the GH31 family, we obtained the crystal structure of the NtMGAM protein with the PDB ID 2QMJ from the Protein Data Bank. The protein was prepared by removing heteroatoms and glycosylated residues. Any missing residues were modeled using a combination of Chimera and Modeller software. A comprehensive list of all metabolites identified in the LC-MS profiling was downloaded in 3D format as.sdf files from PUBCHEM. These metabolites were then subjected to energy minimization using HyperChem-7 and converted into PDF format. Subsequently, docking experiments were conducted for the modeled protein and ligands using AutoDock Vina, which incorporates PyMOL as a plugin (Seeliger and De Groot, 2010; Trott and Olson, 2010). To facilitate the docking procedure, we identified the active site of the protein based on literature findings. The grid dimensions were set to (x=29.184, y=29.795, z=25.403) for rigid docking. A default population size of 100 was chosen in the AutoDock program (Ghanta et al., 2022). The results of ligand binding to the protein were assessed using binding energy calculations, and their interactions were further analyzed using LigPlot+.

### 2.11 Ensemble docking

The limitation posed by protein flexibility stands out as a prominent challenge in the realm of structure-based drug development. When a protein is confined to a singular configuration, essential dynamic aspects of protein-ligand binding may go unnoticed. Despite the existence of a variety of docking techniques designed to accommodate ligand flexibility, studies on protein flexibility have received limited attention until recently (Al-Nema et al., 2023). To address this issue, methodologies such as Monte Carlo and molecular dynamics simulations have been employed. The optimal approach for introducing protein flexibility involves simultaneously exploring and optimizing the complete degree of freedom for both the protein and the ligand (Huang and Zou, 2007). In the context of predicting the binding mechanism of a selected molecule into the α-glucosidase active site, obtained through clustering simulation, an ensemble docking was carried out. This comprehensive approach considers the dynamic nature of both the protein and the ligand, providing a more realistic representation of the binding process (Ghorbani et al., 2023). The protein preparation process involved several key steps to ensure the reliability of subsequent molecular dynamics simulations. Heteroatoms, including glycosylated residues, were removed from the protein structure due to issues with parametrization, and their absence from the active site. To address missing residues, modeling was performed using Chimera and Modeller, with Model 6 selected based on the best Z-DOPE score (-2.14) from the minimization log file. The resulting structure underwent further minimization using Swiss PDB Viewer with the GROMOS force field. Hydrogens were added to the structure for pH 7.4 using PDBFixer, resulting in the file named “2QMJ_rec_pdbfixed.pdb.” To assess the quality of the structure, the Swiss Model Expasy server’s structure assessment tool was employed. Model refinement was conducted through the MODREFINER web server, and the refined model was submitted for assessment. As the refined model closely resembled the modeled structure, a 50 ns molecular dynamics simulation was initiated for the protein in a 1 nm^3^ periodic box, with TIP3P water and 0.15 M NaCl. Upon completion of the 50 ns simulation, clustering analysis based on the RMSD of the backbone of the pocket residues was performed using GROMACS. The trajectory was sampled from 25 ns to 50 ns, and representative structures from the top clusters were extracted. Nine representative structures were used for docking studies for the selected ligands.

### 2.12 Molecular dynamics simulation

The molecular dynamics (MD) simulations for the protein 2QMJ and its ligands (THF, AME, and acarbose) were conducted using GROMACS Version 5 (Abraham et al., 2015). Initially, the protein chain was extracted and the ligands isolated, before recombining them into a single complex file. The topology files for the protein were prepared using the Amber99SB-ILDN (Lindorff-Larsen et al., 2010) force field with gmx pdb2gmx (GROMACS Tutorials, n.d.), while ligand topologies were generated using Antechamber with GAFF parameters (Wang et al., 2004). The system was set up in a dodecahedral box (1.0nm), solvated with SPC/E water molecules, and neutralized with Na+ and Cl- ions to achieve a 0.15 M ionic concentration (Berendsen et al., 1987).

Energy minimization was performed using the steepest descent algorithm, ensuring the maximum force fell below a specified threshold. The system was equilibrated with 0.5 ns NVT (constant volume and temperature) and 0.5 ns NPT (constant pressure and temperature) simulations, applying position restraints to heavy atoms of the protein and ligands. Production MD was carried out over 100 ns with a 2-fs time step, maintaining 300 K temperature with the Berendsen thermostat and 1 bar pressure using the Parrinello-Rahman barostat (Parrinello and Rahman, 1981; Berendsen et al., 1984). Long-range electrostatic interactions were treated using the Particle-Mesh-Ewald method, and periodic boundary conditions were applied throughout (Darden et al., 1993).

Trajectory analysis involved calculating RMSD, RMSD per residue, RMSF, hydrogen bonds, and protein-ligand contacts using GROMACS tools (Hildebrand et al., 2019).Additionally, protein-ligand interactions were examined using LigPlot+, offering insights into the molecular interactions of the protein-ligand complex.

### 2.13 Binding energy calculation

The MM-PBSA (Molecular Mechanics-Poisson-Boltzmann Surface Area) (Kumari et al., 2014) method is a widely used computational approach for estimating binding free energies and modeling molecular recognition, particularly in protein-ligand interactions (Wang et al., 2018, 2019). This method calculates the binding energy by combining several energetic components: the molecular mechanics energy (ΔE_MM_), which includes bonded interactions (bond, angle, and dihedral), electrostatic forces, and van der Waals interactions; the polar solvation energy (ΔGpol), representing the electrostatic contribution from the solvent; the non-polar solvation energy (ΔGnp), accounting for non-polar solvation effects; and the entropy contribution (-TΔS), which captures the entropic effects on binding. The binding free energy (ΔG_bind_) is expressed using the equation:


$$\text{Δ}{\text{G}}_{\text{bind}}=\;\text{Δ}{\text{E}}_{\text{MM}}+\;\text{Δ}\text{Gpol }+\;\text{Δ}\text{Gnp }-\text{ T}\text{Δ}\text{S}$$

This method provides a comprehensive framework for understanding molecular interactions and is invaluable in drug discovery, as it reveals the energetic factors driving binding affinity and stability (Patil et al., 2022).

## 3 Results

### 3.1 Molecular identification

**Figure 1:**
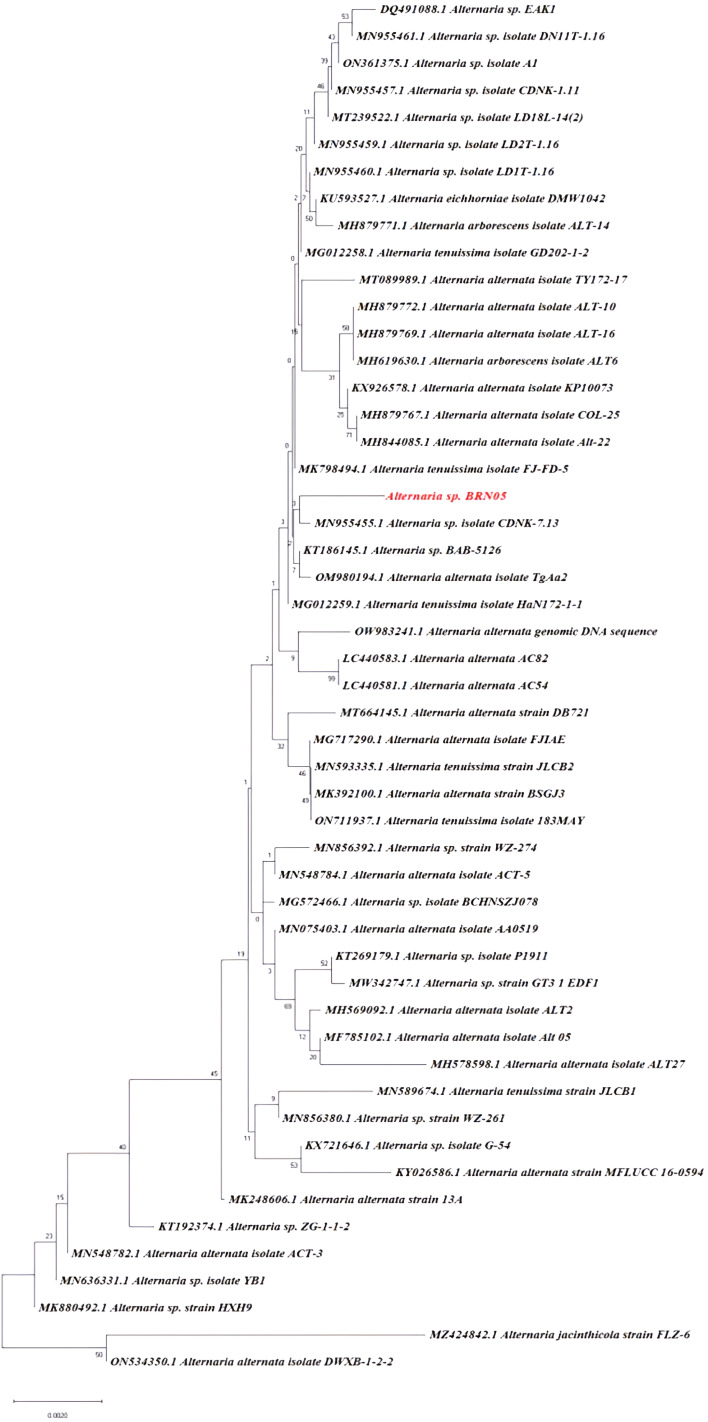
Phylogenetic tree identification of *Alternaria alternata* BRN05 using ITS sequencing.

*Alternaria alternata* BRN05 (Sequence ID: SUB11758282) was identified by employing Internal transcribed spacer (ITS) sequencing method. Phylogenetic analysis of *Alternaria alternata* BRN05 strain was carried out using Neighbour-joining tree method. The sum of branch lengths for the optimal trees was calculated as 0.04229034. To analyze the tree value accurately, the test was carried out a thousand times using bootstrapping technique to ensure that the results were robust and reliable. The phylogenetic tree is given in **Figure 1**. ITS sequences more than 400 bp, combined with ≥99% identity, are considered cutoff for confirming species identity (Romanelli et al., 2010). In our analysis, the ITS sequence obtained was 571 bp in length, with a percentage identity of 99.47%, which supports the identification of the fungus at species level as *Alternaria alternata* BRN05.

### 3.2 Total phenolic content

The total phenolic content in the samples were determined by preparing a calibration curve (**Supplementary File S1B**) generated by plotting different concentrations of gallic acid standards ranging from 1 µg/mL to 10 µg/mL on x-axis and corresponding absorbance (OD) on y-axis. The results were expressed based on linear regression analysis (y = 0.0752x + 0.0068, R^2^ = 0.99) and presented as gallic acid equivalents (GAE) per unit of dry extract weight. EQS sample exhibited a greater phenolic content (11.01 ± 0.06 mg GAE/g) as compared to EFS sample (9.22 ± 0.101 mg GAE/g) as shown in **Table 1**.

**Table 1:** IC_50_ values determined for EFS and EQS against α-glucosidase.

S. No	Sample	IC_50_
1	SMS6FS	1.16 ± 0.17 mg/mL
2	SMS6QS	0.01 ± 1.81 mg/mL

### 3.3 α-Glucosidase inhibition assay

**Figure 2:**
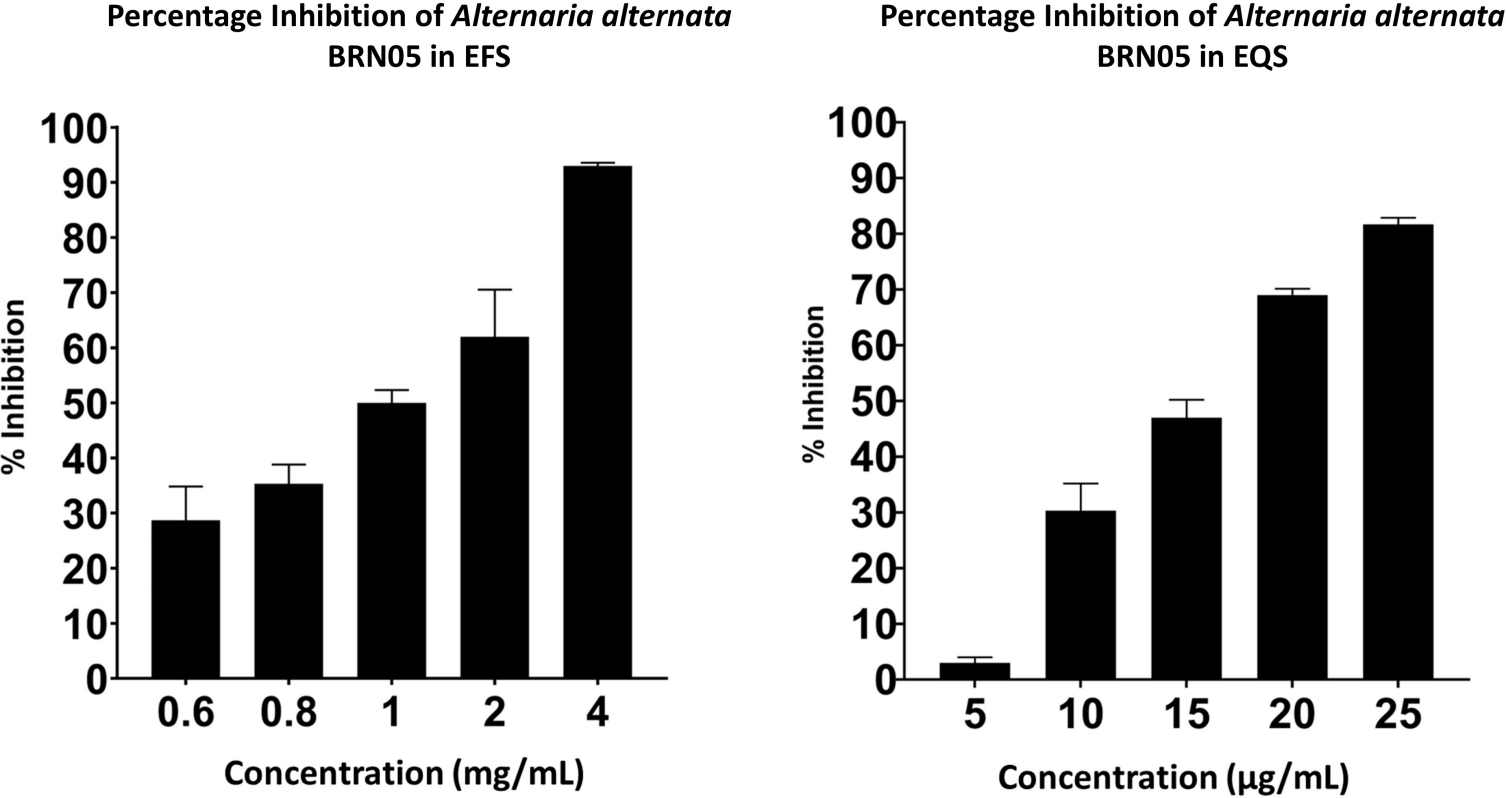
Percentage inhibition of α-glucosidase by EFS and EQS from *Alternaria alternata* BRN05.

The α-glucosidase inhibitory activity of EFS and EQS was carried out. EQS showed a significant IC_50_ value (0.01 ± 1.81 mg/mL), as compared to that of EFS (1.16 ± 0.17 mg/mL) and the positive control, acarbose (0.49 ± 0.01 mg/mL) as shown in **Figure 2**. EQS exhibited stronger α-glucosidase inhibitory activity than that of EFS and acarbose.

### 3.4 Analysis of LC-MS/MS data in negative mode

**Figure 3:**
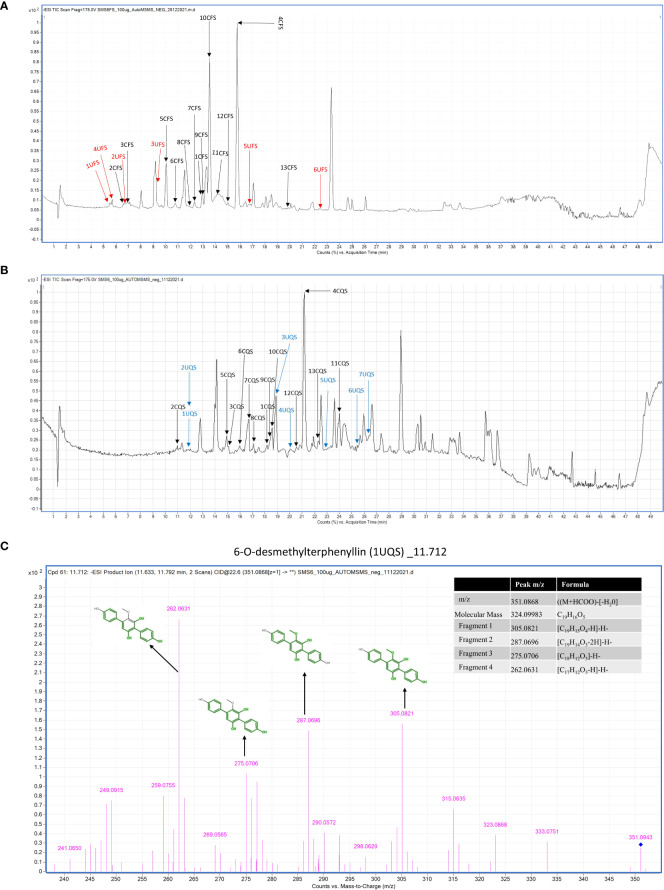
**(A)** Metabolic profiling of *Alternaria alternata* BRN05 was performed using UHPLC-ESI-QTOF-MS in negative ionization mode. The chromatogram revealed thirteen compounds present in the EFS extract. Among these, thirteen compounds were common to both EFS and EQS extracts (1CFS to 13CFS), depicted in black in the chromatogram. Additionally, six compounds were uniquely found in the EFS extract (1UFS to 6UFS), represented in red. **(B)** Metabolic profiling of *Alternaria alternata* BRN05 was performed using UHPLC-ESI-QTOF-MS in negative ionization mode. The chromatogram revealed twenty compounds present in the EQS extract. Among these, thirteen compounds were common to both EFS and EQS extracts (1CQS to 13CQS), depicted in black in the chromatogram. Additionally, seven compounds were uniquely found in the EQS extract (1UQS to 7UQS), represented in blue. **(C)** LC-MS/MS fragmentation of 6-O-desmethylterphenyllin (1UQS) produced ions at m/z 305.082, 287.069, 275.0706, and 262.0631, that matched predicted fragments from MetFrag.

Total Ion Chromatography (TIC) were generated for both samples and the resulting chromatograms are shown in **Figure 3**. A total of 19 and 20 metabolites were identified in EFS and EQS, respectively. 13 metabolites were commonly found in both EFS and EQS, whereas 6 were unique to EFS, and the other 7 were unique to EQS. These compounds have codes assigned in **Table 2**. Compounds from the chromatogram were categorized into “C” and “U” respectively. “C” represents metabolites common to both EFS and EQS, while “U” represents unique metabolites that are found only either in EFS or EQS. Mass spectrometry (MS/MS) fragmentation analysis further confirms the identity of the compounds. The identification process involved comparing observed fragment ions with those predicted by *in silico* fragmentation tool MetFrag, matching against literature-reported fragments, and manual deduction based on chemical knowledge for some compounds, that is shown in **Figure 3C** as one of the examples for one the compounds.

**Table 2A:** Thirteen compounds detected in the ethyl acetate fraction of *Alternaria alternata* BRN05 extract (EFS).

S. No	Retention time (Min)	Compounds	Molecular formula & theoretical molecular mass	*m/z*	Molecular ion form	Identified score	MS/MS fragments
1CFS	12.918	4-Hydroxymellein	C_10_H_10_ O_4_ & 194.0594	221.0456	(M+HCOO)- [-H_2_O]	98.25	177.0561, [C_9_H_5_O_4_]- 162.032, [C_9_H_6_O_3_]- 145.0292, [C_9_H_5_O_4_]- 134.0863, [C_8_H_7_O_2_]-
2CFS	12.318	5’-Epialtenuen	C_15_H_16_O_6_ & 294.0947	291.0874	(M-H)-	98.93	273.0776, [C_15_H_13_O_5_]- 247.0984, [C_14_H_15_O_4_]- 231.0665, [C_13_H_11_O_4_]- 186.0715, [C_11_H_6_O_3_]-
3CFS	6.832	Alternarienonic acid	C_14_H_14_O_6_ & 278.0792	278.0792	(M-H)-	98.67	277.0716, [C_14_H_12_O_6_]- 251.0563, [C_12_H_11_O_6_]- 246.9260, [C_13_H_10_O_5_]- 233.0813,[C_13_H_13_O_4_]-214.09257,[C_13_H_10_O_3_]-189.0542, [C_11_H_9_O_3_]-
4CFS	15.753	(+)-talaroflavone	C_14_H_12_O_6_ & 276.0634	257.0462	(M-H)- [-H_2_O]	96.01	257.0461, [C_14_H_10_O_5_]- 246.9222, [C_13_H_11_O_5_]- 229.0516, [C_13_H_11_O_4_]- 213.0548, [C_13_H_11_O_3_]-
5CFS	10.022	2,5-dimethyl-7-hydroxychromone	C_11_H_10_O_3_ & 190.0630	189.0567	(M-H)-	92.64	189.0567, [C_11_H_9_O_3_]- 174.0324,[C_10_H_6_O_3_]- 160.08424, [C_9_H_4_O_3_]- 119.0491, [C_7_H_3_O_2_]-
6CFS	10.756	Alternarian acid	C_15_H_12_O_8_& 320.0532	319.0464	(M-H)-	96.33	231.0659, [C_13_H_10_O_4_+H]- 203.0699, [C_12_H_10_O_3_+H]- 188.0467, [C_11_H_9_O_3_]-H- 160.8409, [C_10_H_9_O_2_]-H-
7CFS	6.618	1,8-dihydroxynaphthalene	C_10_H_8_O_2_& 160.0524	205.0204	(M+HCOO)-	97.77	149.0601, [C_9_H_8_O_2_]- 118.042, [C_8_H_6_O]- 75.0032, [C_6_H_3_O]- 105.0338, [C_7_H_5_O]-
8CFS	11.999	Aspergone Q	C_11_H_14_O_6_& 242.0790	223.0609	(M-H)- [-H_2_O]	85.48	223.0606, [C_11_H_13_O_5_]- 191.0347,[C_10_H_10_O_4_-2H]-H- 162.0319, [C_9_H_9_O_3_-2H]-H- 122.0383, [C_7_H_9_O_2_-2H]-H-
9CFS	12.967	6-Epi-stemphytriol	C_20_H_16_O_7_& 368.0896	349.0709	(M-H)- [-H_2_O]	96.12	349.0701, [C_20_H_15_O_6_-H]-H- 331.06, [C_20_H_14_O_5_-2H]-H- 145.029, [C_9_H_5_O_2_]- 117.0352, [C_8_H_4_O+H]-
10CFS	13.254	12-Methoxycitromycin	C_14_H_12_O_5_& 260.0685	287.0575	(M+HCOO)- [-H_2_O]	97.80	229.05, [C_13_H_9_O_4_]- 228.0428, [C_13_H_9_O_4_]- 211.0402, [C_13_H_8_O_3_]-H- 200.0476, [C_12_H_8_O_4_]- 243.0665, [C_14_H_11_O_4_]-
11CFS	14.251	4-Hydroxyalternariol 9-methyl ether	C_15_H_12_O_6_& 288.0634	287.0565	(M-H)-	84.00	271.0609, [C_15_H_11_O_5_]- 245.0826, [C_13_H_9_O_5_]- 230.0586, [C_13_H_10_O_4_]- 202.0631, [C_12_H_11_O_3_]- 160.0527, [C_10_H_9_O_2_]-
12CFS	15.056	Orthosporin	C_12_H_12_O_5_& 236.0685	235.0614	(M-H)-	86.45	191.071, [C_11_H_12_O_3_]-H- 189.0549, [C_11_H_11_O_3_-H]-H- 176.0475, [C_10_H_6_O_3_+2H]- 174.0316, [C_10_H_6_O_3_]-
13CFS	19.838	Altenusin	C_15_H_14_O_6_& 290.0790	317.0657	(M+HCOO)- [-H_2_O]	89.62	272.068, [C_15_H_12_O_5_]- 271.016, [C_15_H_11_O_5_]- 270.0149, [C_15_H_10_O_5_]- 242.0223, [C_14_H_10_O_4_]- 198.0313, [C_13_H_10_O_2_]-
1UFS	5.487	4-Ethylcatechol	C_8_H_10_O_2_& 138.0681	119.0501	(M-H)-[-H_2_O]	87.35	119.05, [C_8_H_9_O-H]-H- 93.0340, [C_6_H_4_+H]- 61.9881, [C_2_H_4_O_2_]-
2UFS	6.93	*p*-Coumaric acid	C_9_H_8_O_3_& 164.0473	209.0458	(M+HCOO)-	98.57	123.0453, [C_7_H_7_O_2_]- 81.0343, [C_5_H_6_O]-
3UFS	9.327	Diaportinol	C_13_H_14_O_6_& 266.0790	247.0613	(M-H)-[-H_2_O]	85.17	232.0363, C12H10O5-H]-H- 203.0703, [C12H13O3-H]-H- 188.0469, [C11H8O3]- 161.06, [C10H9O2]-
4UFS	5.677	Phenylacetic acid	C_8_H_8_O_2_& 136.0524	163.04	(M+HCOO)-[-H_2_O]	87.29	119.0504, [C_8_H_7_O]- 91.0549, [C_7_H_7_]- 63.9965, [C_4_H_3_O-2H]-H-
5UFS	16.764	Procyanidin dimer B1	C_30_H_26_O_12_& 578.1425	605.1305	(M+HCOO)-[-H_2_O]	98	486.168, [C_29_H_26_O_2_]- 485.125, [C_29_H_25_O_2_]- 275.0545, [C_14_H_12_O_6_]-H- 241.0491,[C_14_H_12_O_4_-2H]-H- 61.9884, [C_2_H_3_O_2_]-[+H]
6UFS	22.491	Theaflavin	C_29_H_24_O_12_& 564.1268	591.1139	(M+HCOO)-[-H_2_O]	96.34	515.0986, [C_28_H_21_O_10_-H]-H- 203.0709, [C_12_H_10_O_3_-H]- 188.0461[C_11_H_9_O_3_]-H- 92.9968, [C_5_H_4_O_2_-H]-

Among these, six compounds were uniquely present in the EFS extract (1UFS to 6UFS). The provided data includes retention time, molecular formula, observed score, identified score, MS/MS fragments, and theoretical molecular mass.

**Table 2B:** Thirteen compounds detected in the ethyl acetate fraction of *Alternaria alternata* BRN05 extract EQS.

S. No	Retention time (Min)	Compounds	Molecular formula & theoretical molecular mass	m/z	Molecular ion form	Identified score	MS/MS fragments
1CQS	18.181	4-Hydroxymellein	C_10_H_10_O_4_& 194.0579	221.0458	(M+HCOO)- [-H_2_O]	97.72	177.0553, [C_9_H_5_O_4_]- 162.034, [C_9_H_6_O_3_]- 145.0292, [C_9_H_7_O_2_-H]-H-
2CQS	16.664	5’-Epialtenuen	C_15_H_16_O_6_& 292.0947	291.0878	(M-H)-	95.43	291.087, [C_15_H_15_O_6_]- 274.8918, [C_15_H_14_O_5_]- 230.0569, [C_14_H_14_O_3_]- 203.0343, [C_12_H_11_O_3_]-
3CQS	15.025	Alternarienonic acid	C_14_H_14_O_6_& 278.0792	278.0792	(M-H)-	98.43	277.0716, [C_14_H_12_O_6_]- 233.082, [C_13_H_13_O_4_]- 218.0584, [C_12_H_10_O_4_]- 205.086, [C_12_H_13_O_3_]- 190.0639, [C_11_H_9_O_3_]-
4CQS	21.168	(+)-talaroflavone	C_14_H_12_O_6_& 276.0634	257.0465	(M-H)- [-H_2_O]	94.86	257.0457, [C_14_H_10_O_5_]- 228.9590, [C_13_H_8_O_4_]- 215.0355, [C_12_H_7_O_4_]- 202.063, [C_12_H_10_O_3_]-
5CQS	14.807	2,5-dimethyl-7-hydroxychromone	C_11_H_10_O_3_& 190.0630	189.0563	(M-H)-	95.19	189.055, [C_11_H_9_O_3_]- 174.0355, [C_10_H_6_O_3_]- 162.8384, [C_10_H_10_O_2_]- 105.0328, [C_11_H_7_O]- 146.0358, [C_9_H_7_O_2_]-H-
6CQS	15.882	Alternarian acid	C_15_H_12_O_8_& 320.0532	319.0461	(M-H)-	98.03	231.0656, [C_13_H_10_O_4_+H]- 216.0434, [C_12_H_9_O_4_]-H- 203.0712, [C_12_H_10_O_3_+H]- 188.0476, [C_11_H_9_O_3_]-H-
7CQS	10.951	1,8-dihydroxynaphthalene	C_10_H_8_O_2_& 160.0524	205.0509	(M+HCOO)-	96.46	143.0502, [C_10_H_7_O]- 118.9939, [C_8_H_6_O]- 105.0351, [C_7_H_5_O]-
8CQS	16.959	Aspergone Q	C_11_H_14_O_6_& 242.07908	223.0611	(M-H)- [-H_2_O]	84.46	191.0352,[C_10_H_10_O_4_-2H]-H- 149.0247, [C_8_H_7_O_3_-H]-H- 123.0462, [C_7_H_9_O_2_-H]-H-
9CQS	18.313	6-Epi-stemphytriol	C_20_H_16_O_7_& 368.0896	349.0716	(M-H)- [-H_2_O]	99.01	331.0592, [C_20_H_14_O_5_-2H]-H- 292.061, [C_14_H_12_O_7_]- 243.0665, [C_14_H_11_O_4_]-
10CQS	18.569	12-Methoxycitromycin	C_14_H_12_O_5_& 260.0685	305.0665	(M+HCOO)-	93.62	229.05, [C_13_H_9_O_4_]- 228.0424, [C_13_H_8_O_4_]- 211.0368, [C_13_H_8_O_3_]-H- 200.0467, [C_12_H_8_O_3_]-
11CQS	23.981	4-Hydroxyalternariol 9-methyl ether	C_15_H_12_O_6_& 288.0634	287.0571	(M-H)-	92.02	287.0571, [C_15_H_11_O_6_]- 272.0331, [C_14_H_8_O_6_]- 257.0181, [C_13_H_5_O_6_]- 228.0422, [C_14_H_12_O_3_]- 188.047, [C_11_H_9_O_3_]-H-
12CQS	20.357	Orthosporin	C_12_H_12_O_5_& 236.0685	235.061	(M-H)-	84.64	191.0711, [C_11_H_12_O_3_]-H- 189.0556, [C_11_H_11_O_3_-H]-H- 176.0472, [C_10_H_6_O_3_+2H]- 161.0248, [C_9_H_4_O_3_+H]- 148.0522, [C_9_H_7_O_2_+H]-
13CQS	22.856	Altenusin	C_15_H_14_O_6_& 290.0790	317.0666	(M+HCOO)- [-H_2_O]	98.27	271.024, [C_15_H_11_O_5_]- 270.169, [C_15_H_10_O_5_]- 242.0206, [C_14_H_10_O_4_]- 214.0267, [C_13_H_10_O_3_]- 198.0313, [C_13_H_10_O_2_]-
1UQS	11.821	6-O-desmethylterphenyllin	C_19_H_16_O_5_& 324.0998	351.0868	(M+HCOO)-[-H_2_0]	83.05	305.0821, [C_19_H_15_O_4_-H]-H- 287.0696, [C_19_H_14_O_3_-2H]-H- 275.0706, [C_18_H_12_O_3_]-H- 262.0631, [C_17_H_12_O_3_-H]-H-
2UQS	11821	Altertoxin I	C_20_H_16_O_6_& 352.0947	351.0868	(M-H)-	83.05	333.0751, [C_20_H_15_O_5_-H]-H- 305.0821, [C_19_H_13_O_4_]- 290.0572, [C_18_H_11_O_4_]-H- 262.0631, [C_17_H_11_O_3_]-H-
3UQS	18.881	Altechromone B	C_14_H_14_O_6_& 246.0892	245.0823	(M-H)-	96.66	230.0574, [C_13_H_11_O_4_]-H- 188.0488, [C_11_H_9_O_3_]-H- 160.0532, [C_14_H_14_O_4_] 146.0376, [C_9_H_7_O_2_]-H-
4UQS	20.123	Botryorhodine F	C_16_H_14_O_6_& 302.0790	347.0768	(M+HCOO)-	83.38	259.0955, [C_15_H_14_O_4_+H]- 241.0871, [C_15_H_13_O_3_]- 226.0638, [C_14_H_11_O_3_]-H- 271.0864, [C_13_H_13_O_3_]-
5UQS	22.856	3’,4’,7-Trihydroxyisoflavanone	C_15_H_12_O_5_& 272.0685	317.0666	(M+HCOO)-	98.27	271.053, [C_15_H_11_O_5_]- 270.053, [C_15_H_10_O_5_]- 242.0206, [C_14_H_10_O_4_]- 226.0271, [C_14_H_10_O_3_]- 214.0267, [C_12_H_7_O_4_-H]- 198.0313, [C_12_H_8_O_3_-H]- 256.038, [C_14_H_10_O_5_-H]-H-
6UQS	25.38	Alternariol 9-methyl ether	C_15_H_12_O_5_& 272.0685	317.0669	(M+HCOO)-	97.89	271.061, [C_15_H_11_O_5_]- 270.061, [C_15_H_10_O_5_]- 198.0338, [C_13_H_10_O_2_]- 242.0206, [C_13_H_6_O_5_]-
7UQS	26.28	Morin	C_15_H_10_O_7_& 302.0426	301.0349	(M-H)-	97.5	301.0349, [C_14_H_9_O_7_]- 273.0399, [C_14_H_9_O_5_]- 258.0159, [C_13_H_8_O_6_-H]-H- 230.0240, [C_12_H_7_O_5_]-H-

Among these, seven compounds were uniquely present in the EQS extract (1UQS to 7UQS). The provided data includes retention time, molecular formula, observed score, identified score, MS/MS fragments, and theoretical molecular mass.

**Table 1** contains the list of compounds with assigned code, retention time, tentative or putative compound proposed, molecular formula, *m/z*, molecular ion form, identified score, MS/MS fragments, and the total number of fragments identified using *in-silico* fragmentation tool MetFrag (Ruttkies et al., 2016). While the table provides a comprehensive overview, key details regarding MS-MS fragmentation are elucidated in the subsequent paragraphs.

The analysis revealed several compounds with common fragment ions observed in both CFS and CQS samples. 4-hydroxymellein (1CFS and 1CQS) showed fragment ions at m/z 177.0561 and 145.0292, with a characteristic ion at m/z 162.8385 corresponding to the loss of methanol [(M-H)-CH_3_OH]. 5’-Epialtenuen (2CFS and 2CQS) exhibited fragments at m/z 273, 247, 231, and 186, with the latter resulting from sequential losses of water, carbon dioxide, formaldehyde, methanol, and a methyl radical. Alternarienonic acid (3CFS and 3CQS) displayed characteristic ions at m/z 277 and 233, corresponding to the loss of a proton and carbon dioxide, respectively and is reported in literature (Kjer, 2009).

(+)-talaroflavones (4CFS and 4CQS) showed a fragment ion at m/z 257, representing the loss of water [(M-H)-H_2_O]-, which was consistent with a fragment reported by Zhao et al. (2021). 2,5-dimethyl-7-hydroxychromone (5CFS and 5CQS) exhibited common fragments at m/z 189 and 174, corresponding to the loss of a proton and a methyl group. A fragment at m/z 146, previously reported in the literature, was also observed. Alternarian acid (6CFS and 6CQS) showed characteristic ions at m/z 160.08, 188.04, 203.0712, and 213.06, with the fragment at m/z 188.04 also reported in the literature (Kjer, 2009).

Other compounds analyzed included p-Coumaric acid, which showed fragment ions at m/z 123.0453, 95.0129, 65.0396, and 81.0343, consistent with literature data (LC-MS/MS Spectrum - LC-ESI-QTOF (UPLC Q-Tof Premier, Waters), Negative (DB04066) | DrugBank Online, n.d.). Phenylacetic acid exhibited fragment ions at m/z 119.504 and 91.0504, as reported in the literature (Phenylacetic acid Mass Spectrum, n.d.). Procyanidin dimer B showed fragment ions at m/z 485.1245, 275.0545, and 241.049, matching those reported in the literature (Procyanidin B1 Mass Spectrum, n.d.). Theaflavin displayed a fragment ion at m/z 288.0622, consistent with literature reports (Verloop, 2016).

Altertoxin I showed a molecular ion at m/z 333.0751, reported as a key fragment ion by Puntscher et al. (2019), and another significant fragment ion at m/z 290.0572, described by Stack et al. (1986). 3’,4’,7-trihydroxyisoflavanone exhibited fragment ions at m/z 270.05 and 242.02, confirmed from the literature (Human Metabolome Database: LC-MS/MS Spectrum - ESI-TOF 30V, Negative (HMDB0041655), n.d.). Alternariol 9-methyl ether showed a characteristic fragment ion at m/z 242.0206, as reported in the literature (Burkhardt et al., 2011). Lastly, Morin displayed fragment ions at m/z 273.0399 and 258.0159, aligning with those reported by McNab et al. (2009). Additional information about the MS/MS spectrum of each molecule, as well as the corresponding fragmentation pattern, is provided in **Supplementary File S2**.

### 3.5 Molecular docking

Docking was performed for the twenty-six metabolites identified from EFS and EQS, and all resulting data are provided in **Supplementary File S3A**. Twelve compounds from EFS and EQS were selected which exhibited greater binding affinity for the α-glucosidase compared to acarbose (**Table 3**). All seven compounds (6’-O-Desmethylterphenyllin, Altertoxin I, Altechromone B, Botryorhodine F, THF, AM and Morin) unique to EQS demonstrated significantly higher binding energies than acarbose. Additionally, one compound from UFS (p-Coumaric acid) and three compounds common to both CFS and CQS (6-Epi-stemphytriol, 1,8-hydroxynaphthalene, and 5’-Epialtenuen) also exhibited better binding energy than acarbose. Based on higher binding energy and hydrogen bond interactions THF (-7.5 kcal/mol) and AME (-7.0 kcal/mol) were selected for further analysis. A 2D LigPlot+ image of all 12 compounds is presented in **Supplementary File S3B**.

**Table 3:** Twelve compounds identified from EFS and EQS exhibiting greater α-glucosidase inhibitory activity compared to acarbose.

Code	Compound name	Best pose binding energy (kcal/mol)	RMSD	H-bond interaction	No. of hydrogen bonds
2UQS	Altertoxin I	-7.8	4.518	Asp203	1
5UQS	3’,4’,7-Trihydroxyisoflavanone	-7.5	1.811	Arg526, Asp571, Gly541, Trp539, His600	5
7UQS	Morin	-7.5	2.805	Asp327, Asp542, Arg526, Asp203	4
9CFS & 9CQS	6-Epi-stemphytriol	-7.2	1.544	Arg334(2), Glu404	2
6UQS	Alternariol 9-methyl ether	-7	3.741	Arg526, Asp571, Trp539, His600	4
3UFS	Diaportinol	-7	1.569	Asp327, His600, Asp542, Arg526	4
1UQS	6’-O-Desmethylterphenyllin	-6.9	1.443	Asp327, His600	2
3UQS	Altechromone B	-6.8	2.083	Arg526	1
4UQS	Botryorhodine F	-6.7	1.521	Tyr605, Gln603, Asp203	3
2UFS	*p*-Coumaric acid	-6.7	0.563	Asn543, Ser553, Asp549	3
2CFS & 2CQS	1,8-dihydroxynaphthalene	-6.7	0.093	Asp443, Arg526	3
7CFS & 7CQS	5’-Epialtenuen	-6.7	1.757	Met444, Asp443, Arg526	3
Positive Ctrl	Acarbose	-6.6	4.668	Trp406, Asp443, Asp327, His600, Arg334	5

### 3.6 Ensemble docking

**Figure 4:**
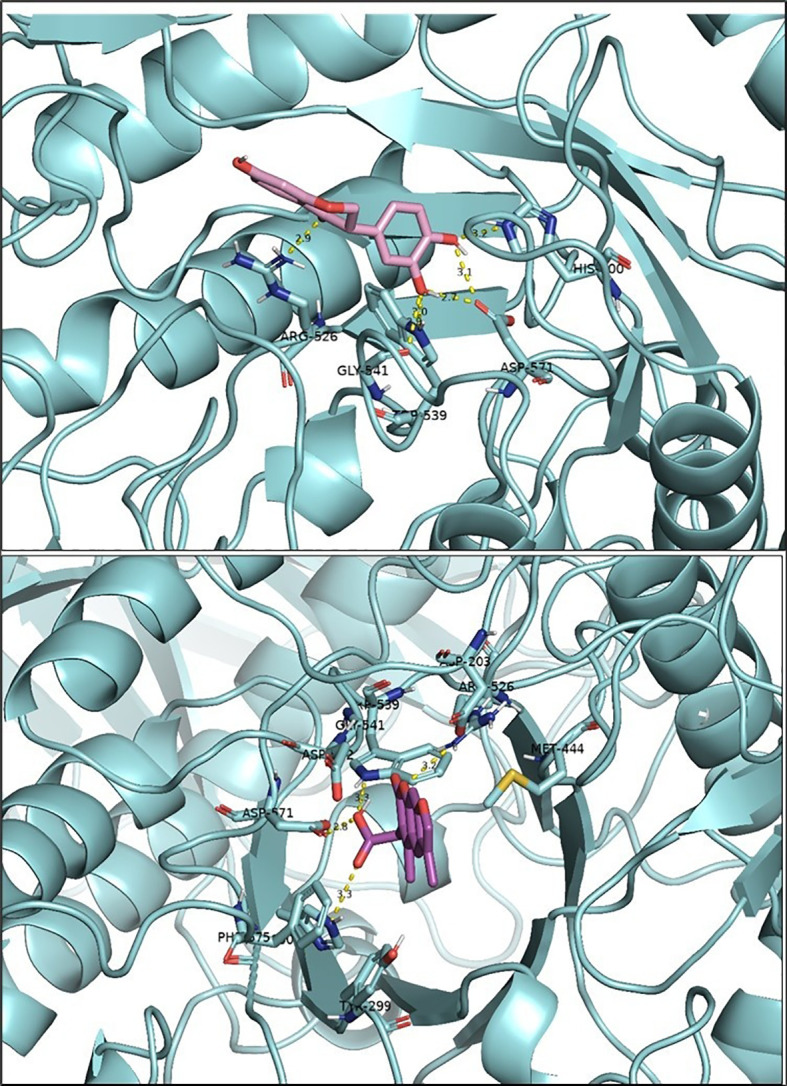
Docking results for two ligands: 3’,4’,7-trihydroxyisoflavanone (top) and Alternariol 9-methyl ether (bottom). The protein is represented in cyan, and hydrogen bonds between the ligands and the amino acid residues of the active site are shown in yellow. The figure was generated using PyMOL software.

Ensemble docking was carried out for the THF and AME with 2QMJ. Docking was carried out with nine different poses of 2QMJ obtained through simulation studies. In the case of THF, the first pose, labeled 2QMJ01_ THF, exhibited no hydrogen bonds. In the second pose, 2QMJ02_ THF, five hydrogen bonds were observed: Arg526, Asp571, Gly541, Trp539, and His600, as seen in **Figure 4**. The third pose, 2QMJ03_ THF, exhibited a single hydrogen bond with Tyr 299. The fourth pose, 2QMJ04_ THF, displayed three hydrogen bonds: Trp406 and Asp203 (2). In the fifth pose, 2QMJ05_ THF, three hydrogen bonds were observed with Arg334, Asp366, and Trp406. The sixth pose, 2QMJ06_ THF, demonstrated a total of three hydrogen bonds involving Asp571 (2) and His600. The seventh pose, 2QMJ07_ THF, exhibited one hydrogen bond with His600. The eighth pose, 2QMJ08_ THF, displayed one hydrogen bond: Asp542. The ninth pose, 2QMJ09_ THF, exhibited three hydrogen bonds involving Trp441 and Arg598 (2). All nine poses are displayed in **Supplementary File S3C**. A few amino acids like Asp443 and Met444, and Trp539 were observed across different poses.

Ensemble docking was performed for the nine different poses of 2QMJ with AME as the ligand. In the first pose, labeled 2QMJ01_ AME, no hydrogen bonds were observed. In the second pose, 2QMJ02_ AME, three hydrogen bonds were observed: Trp539, His600 (2). The third pose, 2QMJ03_ AME, exhibited one hydrogen bond with Asp329. The fourth pose, 2QMJ04_ AME, displayed four hydrogen bonds: Arg526, Asp571, Trp539, His600, as depicted in **Figure 4**. In the fifth pose, 2QMJ05_ AME, four hydrogen bonds were observed: Tyr299, Trp406, Arg335, Arg334. The sixth pose, 2QMJ06_ AME, demonstrated a total of two hydrogen bonds involving His600 (2). The seventh pose, 2QMJ07_ AME, exhibited two hydrogen bonds with His600 (2). The eighth pose, 2QMJ08_ AME, displayed two hydrogen bonds: Asp327, Trp406. The ninth pose, 2QMJ09_ AME, exhibited two hydrogen bonds involving Asp327, His600. Amnio acid like Asp 327 was observed across different poses. All the nine poses 2D images are shown in **Supplementary File S3D**.

### 3.7 Molecular dynamics simulation

The molecular dynamics simulation results provided valuable insights into the behavior of THF and AME interacting with the human α-glucosidase (2QMJ). The analysis focused on RMSD, RMSF, number of hydrogen bonding, LigPlot+ interactions, and MM-PBSA calculations.

### 3.8 RMSD analysis

**Figure 5:**
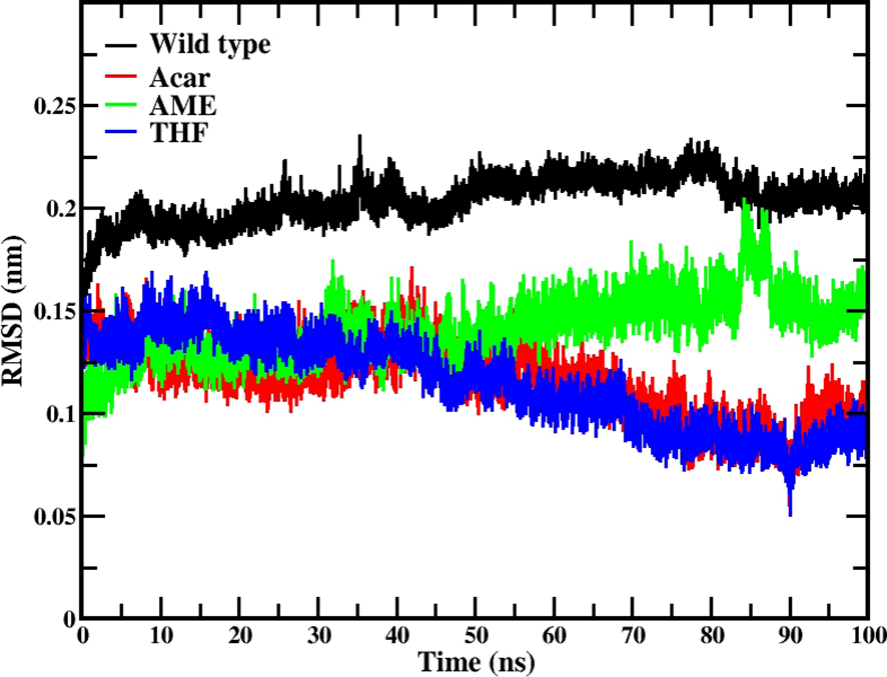
RMSD analysis for protein-ligand complexes during 100 ns molecular dynamics simulations. Data is shown for the native protein (black), Acarbose (positive control, red), 3’,4’,7-trihydroxyisoflavanone (THF, dark blue), and alternariol 9-methyl ether (AME, green).

The RMSD analysis revealed that all three ligand complexes demonstrated better structural stability compared to the native type. The native type (black line) maintained higher RMSD values around 0.2 nm throughout the simulation. THF (blue line) showed initial fluctuations around 0.15 nm and gradually decreased over time, reaching its lowest RMSD values of approximately 0.08-0.09 nm between 80-90 ns, indicating a very stable binding configuration. Acarbose exhibited a similar decreasing trend in RMSD over time, suggesting stabilization by the end of the simulation. The AME trajectory (green line) demonstrated an increasing trend in RMSD values, starting from around 0.1 nm and reaching approximately 0.15-0.17 nm by the end of the simulation. By the end of the 100 ns simulation, all protein-ligand complexes had reached a stable state, and it is shown in **Figure 5**.

### 3.9 RMSF analysis

**Figure 6:**
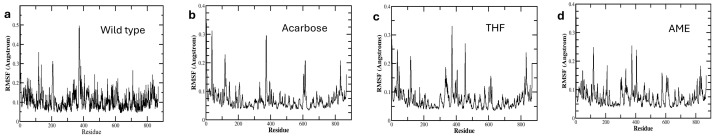
Root Mean Square Fluctuation (RMSF) Analysis of amino acid residues in Complex with Different Ligands During 100 ns Molecular Dynamics Simulation. The plots show residue flexibility of **(A)** native protein **(B)** protein complexed with Acarbose, **(C)** 3’,4’,7-trihydroxyisoflavanone (THF), and **(D)** alternariol 9-methyl ether (AME).

The RMSF analysis highlighted differences in protein flexibility among the complexes. All complexes, including the native protein, showed high flexibility in residue 374, part of the inserted loop 1 domain. The native protein exhibited the most extensive fluctuations, indicating greater overall flexibility compared to the protein-ligand complexes. THF, AME, and Acarbose systems showed more localized high fluctuations, suggesting stabilization of certain regions compared to the native protein. Residue 119 consistently showed high fluctuations across all complexes, indicating a naturally flexible region independent of ligand binding. The region around residues 605-615 showed medium to high fluctuations in all protein-ligand complexes, which may be important for protein function or ligand binding. Notably, while residue 405 exhibited high flexibility in the native protein, it showed less pronounced fluctuations in the THF, AME, and Acarbose complexes, potentially preventing the widening of the active site (Zhang et al., 2021; Kannakazhi Kantari et al., 2024) illustrated in **Figure 6**.

### 3.10 Number of hydrogen bonding

**Figure 7:**
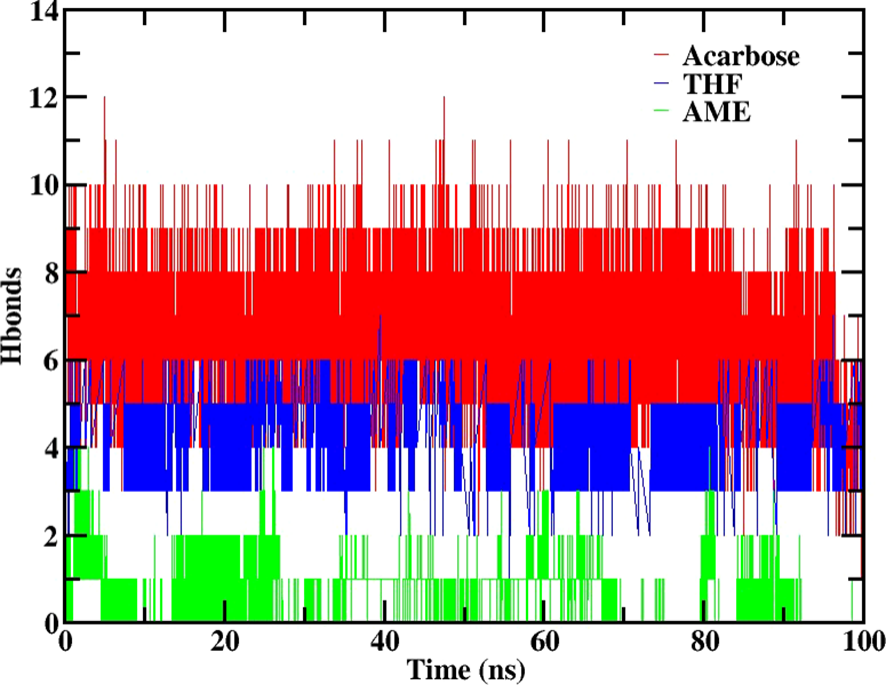
Number of hydrogen bonds formed between 2QMJ and ligands during 100 ns molecular dynamics simulation. Data is shown for the native protein (black), Acarbose (positive control, red), 3’,4’,7-trihydroxyisoflavanone (THF, dark blue), and alternariol 9-methyl ether (AME, green).

The hydrogen bonding analysis at 100 ns revealed distinct patterns for each ligand. THF maintained a moderate level of hydrogen bonding, with 3 to 4 hydrogen bonds observed. AME showed no hydrogen bonding, suggesting a different mode of interaction with the protein. Acarbose demonstrated a reduction in hydrogen bonding, decreasing from 7 to 5 hydrogen bonds over the course of the simulation and it is depicted in **Figure 7**.

### 3.11 LigPlot+ analysis

**Figure 8:**
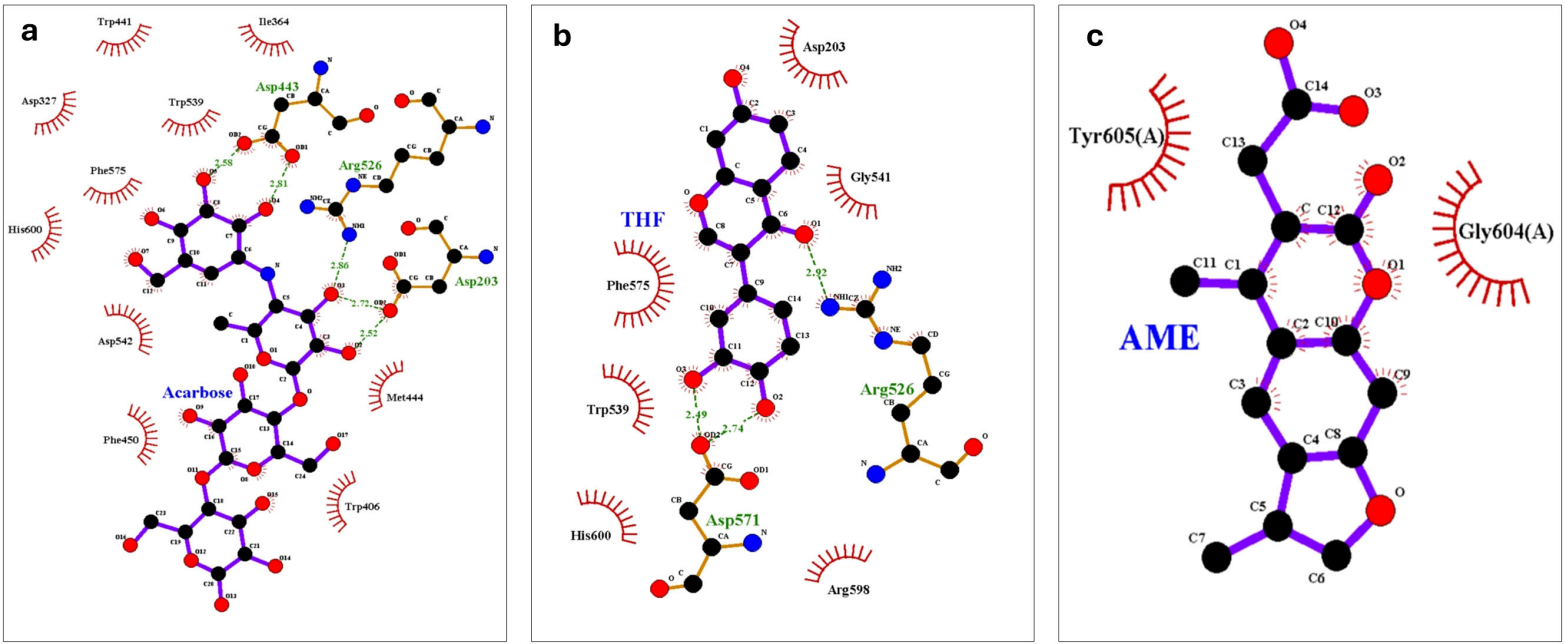
LigPlot+ analysis of protein-ligand interactions at the end of 100 ns molecular dynamics simulations of **(A)** Acarbose, **(B)** 3’,4’,7-trihydroxyisoflavanone (THF), and **(C)** alternariol 9-methyl ether (AME).

The LigPlot+ analysis provided insights into specific interactions between the ligands and protein residues. THF interacted with key residues including Asp203, Gly541, Arg526, Asp571, and Arg598, with hydrophobic interactions observed with Phe575, Trp539, and His600. AME showed a simpler interaction profile, primarily engaging with Tyr605(A) and Gly604(A) through hydrophobic interactions. Acarbose formed hydrogen bonds with Asp203 and Arg526, interacted with the catalytic residue Asp443, and engaged with Trp406 in the inserted loop region, which is significant for inhibitor stability (Zhang et al., 2021) and seen in **Figure 8**.

### 3.12 MMPBSA analysis

The MM-PBSA analysis revealed significant variations in binding energetics among the ligands. Acarbose and THF demonstrated the strongest binding affinity with total binding energies of -144.94 ± 32.22 kJ/mol and -87.07 ± 12.927 kJ/mol, respectively. AME showed a weaker binding affinity with a total binding energy of -55.42 ± 21.42 kJ/mol. Electrostatic forces were the primary drivers of favorable interactions, with Acarbose and THF exhibiting strong electrostatic energies of -169.34 ± 31.44 kJ/mol and -124.30 ± 12.29 kJ/mol, respectively. Van der Waals contributions followed a similar trend, with Acarbose and THF showing strong interactions of -137.30 ± 18.31 kJ/mol and -103.48 ± 13.17 kJ/mol, respectively. Acarbose exhibited a higher polar solvation energy (181.06 ± 38.17 kJ/mol) compared to THF (153.87 ± 11.78 kJ/mol) and AME (28.13 ± 32.15 kJ/mol). Overall, THF, with a binding energy of -87.08 ± 12.92 kJ/mol, showed potential as an alpha-glucosidase inhibitor, warranting further experimental studies for confirmation and it is presented in **Table 4**.

**Table 4:** MM-PBSA energy components and binding free energy for protein-ligand complexes [Acarbose, 3’,4’,7-trihydroxyisoflavanone (THF), and alternariol 9-methyl ether (AME)].

Energy components (kJ/mol)	2qmj_Acarbose	2qmj_THF	2qmj_AME
van der Waal energy	-137.30	-103.48	-54.91
Electrostatic energy	-169.34	-124.29	-22.02
Polar solvation energy	181.06	153.87	28.13
SASA energy	-19.36	-13.17	-6.46
Binding energy	-144.94	-87.08	-55.42

## 4 Discussion

Enzyme α-glucosidase breaks down complex carbohydrates into simple sugars in the intestine, which are readily absorbed into the blood stream resulting in a sudden spike in blood glucose levels after a meal. Therefore, inhibiting α-glucosidase activity is a key strategy for managing diabetes. While α-glucosidase inhibitor drugs currently available in the market control sugar levels, they often produce some side effects as well. Bioactive metabolites from endophytic fungi hold promise as effective α-glucosidase inhibitors with minimal or no side effects for lowering postprandial blood glucose levels in diabetic patients (Naveen and Baskaran, 2018). Phenolic compounds produced by endophytic fungi through metabolic pathways exhibit both antioxidant properties as well as α-glucosidase enzyme inhibitory effects. Because of their unique chemical and structural properties, these phenolic compounds present themselves as potential metabolites with α-glucosidase inhibiting activity (Akhtar et al., 2018).

Nutrient availability in the culture medium in which endophytic fungi are grown has a profound impact on their growth and metabolism. Alteration in media composition brings about differential activation of BGCs leading to synthesis of certain set of secondary metabolites. Partial starvation or limited nutrient availability occasionally leads to activation of specific BGCs resulting in synthesis of a set of metabolites with potential therapeutic value (Pan et al., 2019). In our study, metabolites from EQS exhibited significantly higher IC_50_ values for α-glucosidase inhibition as compared to those from EFS. Further, EQS also exhibited higher phenolic content as compared to EFS.

Bioactivity-guided fractionation is a vital method for isolating and studying bioactive compounds from fungal extracts. While fractionation is lengthy, time-consuming and challenging due to low concentrations of bioactive compounds in the extracts, dereplication offers a quicker alternative for tentative identification of compounds based on those already reported, without having to isolate them. Our study employed a dereplication approach involving UHPLC-ESI-QTOF-MS for tentative identification of compounds. LC-MS, being highly sensitive can tentatively identify metabolites during the first fractionation step itself and there by facilitate targeted fractionation of the desired compounds (Ito and Masubuchi, 2014). Several compounds identified in LC-MS are known to exhibit α-glucosidase inhibitory activity. 4-hydroxymellein exhibited IC_50_ value around 549 µM (Mata et al., 2018), Alternarienonic acid showed 7.95± 1.2 µM (Elbermawi et al., 2022), (+)-talaroflavone exhibited IC_50_ more than 300 µM (Elbermawi et al., 2022), and Altenusin showed IC_50_ value at 46.14± 0.84 µM (Elbermawi et al., 2022) with inhibitory effects. p-Coumaric acid demonstrated significant inhibition with IC_50_ values ranging from 99.8 ± 0.2 µM (Takahashi and Miyazawa, 2012), 6.20 ± 0.04 mM (Aleixandre et al., 2022) and > 150 uM (Jeong et al., 2012) across different studies. Diaportinol showed 43% inhibition at 200 µM (Cai et al., 2018). Procyanidin dimer B1 exhibited IC50 values between 68.75 µg/ml (Zhang et al., 2015), 0.29 mg/ml (Bellesia and Tagliazucchi, 2014) and 68.75 µg/ml (Wang et al., 2021). Theaflavin displayed potent inhibition with an IC50 of 0.036 mg/ml (Sun et al., 2021). Other potent inhibitors include 6-O-desmethylterphenyllin (IC_50_ of 0.9 μM) (Huang et al., 2011; Debbab et al., 2013), Altertoxin I (IC_50_ 9.8 ± 0.3 µM) (Chen et al., 2015), and Botryorhodine F (IC_50_ 12.01± 1.2 µg/ml (Ali et al., 2023), Morin (IC_50_ 4.48 ± 0.04 µM) (Zeng et al., 2016). These compounds likely contributed to the observed α-glucosidase activity in the study.

To further confirm the observed α-glucosidase activity, molecular docking analyses were performed with the identified molecules. Several reported molecules, including Morin, Altertoxin I and 6-Epi-stemphytriol, exhibited high binding energies in docking studies. Notably, THF and AME demonstrated strong binding affinities with binding energies of -7.5 kcal/mol and -7.0 kcal/mol, respectively, and formed multiple hydrogen bonds. These compounds, along with other molecules from EQS displaying high binding energies, could synergistically contribute to the observed α-glucosidase inhibitory activity.

MD simulation provided insight into the interaction of THF and AME with 2QMJ. THF proved to be a potential molecule with a high total binding energy of -87 kJ/mol and key interactions with Phe575, as well as an H bond with Arg526, as observed in Acarbose (Zhang et al., 2021) AME, with a total binding energy of -55 kJ/mol and very limited interaction with the active site, was found to be a less potent AGI. This is the first time we are reporting THF as a potential candidate as an antidiabetic drug.

The key finding of this study is that *Alternaria alternata* BRN05 grown in EQS exhibited greater potential for producing bioactive secondary metabolites compared to those grown in EFS, highlighting the importance of nutrient concentration in modulating fungal metabolite production. Secondly, THF obtained from EQS is found to be a potential AGI based on docking and MD simulation analysis. Thirdly, this study unveils several previously unknown metabolites from *Alternaria* species, expanding our knowledge and understanding of fungal metabolism and opening more avenues for bioactive compound discovery. Moving forward, we aim to undertake large-scale cultivation of the fungi, purification of the desired metabolites, and NMR-based characterization.

## 5 Conclusion

In conclusion, this study highlights the potential of *Alternaria alternata* BRN05, isolated from *Swietenia macrophylla* King, is a source of novel bioactive metabolites with α-glucosidase inhibitory activity. Cultivation in quarter-strength media significantly enhances α-glucosidase inhibitory activity compared to full-strength media. Metabolic profiling identified 19 compounds from EFS and 20 compounds from EQS. Molecular docking reveals that 12 compounds exhibited superior binding energies compared to acarbose. Notably, MD simulation of THF demonstrated stable interactions with the active site with high binding energy of - 87.08 ± 12.93 kJ/mol and key interactions with residues Arg526 and Phe575 confirming its potential as α-glucosidase inhibitor. Thus, compounds from *Alternaria* extract can be potential therapeutic agents that are more effective in the treatment of diabetes.

## Data Availability

The original contributions presented in the study are included in the article/**Supplementary Material**. Further inquiries can be directed to the corresponding author.
