# Supplementary material for: Elucidation of α-glucosidase inhibitory activity and UHPLC-ESI-QTOF-MS based metabolic profiling of endophytic fungi Alternaria alternata BRN05 isolated from seeds of Swietenia macrophylla king
Source: Front Fungal Biol. 2025 Jan 28;6:1447609. doi: 10.3389/ffunb.2025.1447609 (PMC11811940; doi:10.3389/ffunb.2025.1447609)
Supplement: Supplementary file 1 [file DataSheet1.pdf]

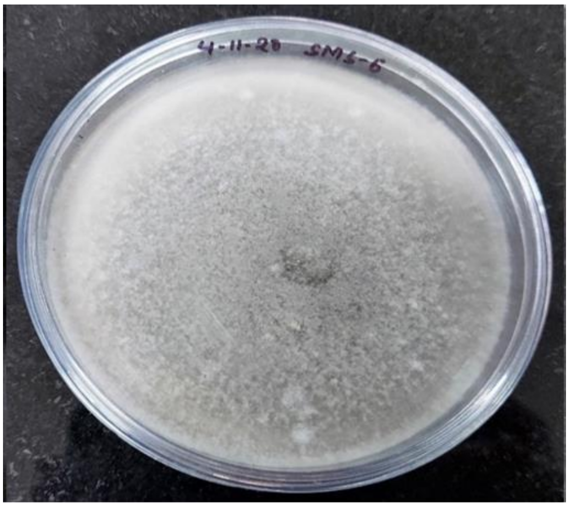

Supplementary file 1 (A). Endophytic fungi isolated from the seeds of *Swietenia macrophylla* cultured on PDA.

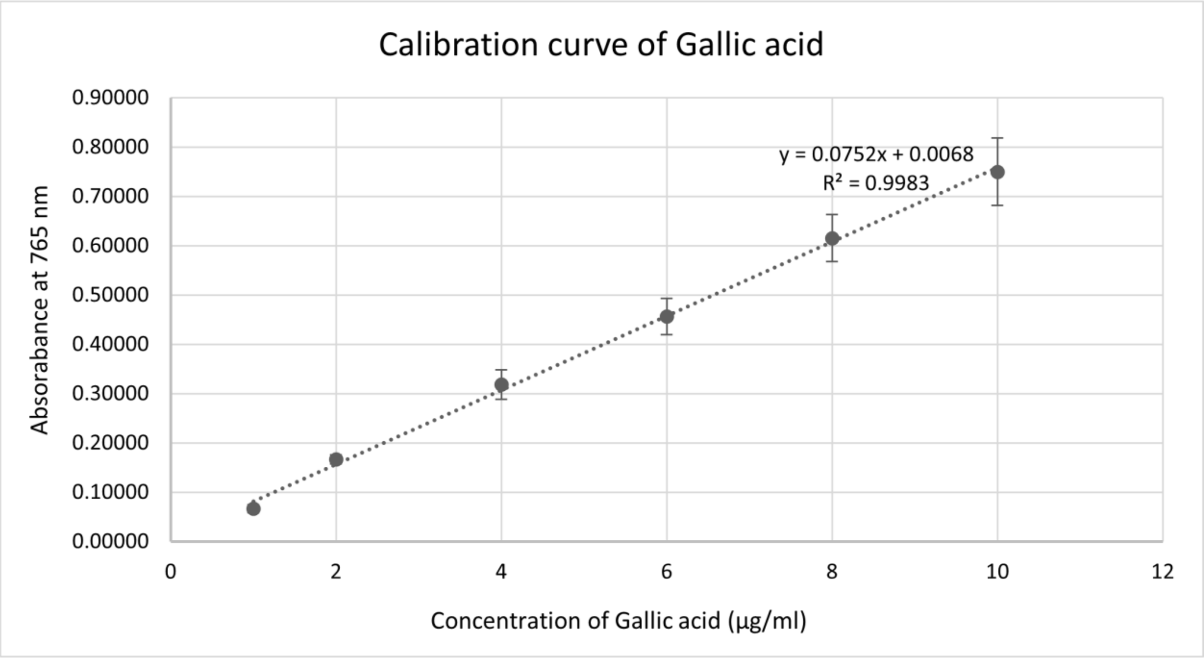

Supplementary file 1 (B). Calibration curve for Gallic acid expressed in gallic acid equivalents per gram of dry extract weight
