# Supplementary material for: Elucidation of α-glucosidase inhibitory activity and UHPLC-ESI-QTOF-MS based metabolic profiling of endophytic fungi Alternaria alternata BRN05 isolated from seeds of Swietenia macrophylla king
Source: Front Fungal Biol. 2025 Jan 28;6:1447609. doi: 10.3389/ffunb.2025.1447609 (PMC11811940; doi:10.3389/ffunb.2025.1447609)
Supplement: Supplementary file 2 [file DataSheet2.pdf]

S. No	Compound Name	Molecular Formula	Pose-1 Binding energy (kcal/mol)	RMSD
	**Molecules common to The EFS and EQS**			
1CFS & 1CQS	4-hydroxymellein	$C_{10}H_{10}O_4$	-6	1.168
2CFS & 2CQS	1,8-dihydroxynaphthalene	$C_{15}H_{16}O_6$	-6.7	0.093
3CFS & 3CQS	Alternarienonic acid	$C_{14}H_{14}O_6$	-6.1	1.953
4CFS & 4CQS	(+)-talaroflavone	$C_{14}H_{12}O_6$	-6.5	2.833
5CFS & 5CQS	2,5-dimethyl-7-hydroxychromone	$C_{11}H_{10}O_3$	-5.5	1.581
6CFS & 6CQS	Alternarian acid	$C_{15}H_{12}O_8$	-6.5	1.403
7CFS & 7CQS	5'-Epialtenuen	$C_{10}H_8O_2$	-6.7	1.757
8CFS & 8CQS	Aspergone Q	$C_{11}H_{14}O_6$	-6.4	1.134
9CFS & 9CQS	6-Epi-stemphytriol	$C_{20}H_{16}O_7$	-7.2	1.544
10CFS & 10CQS	12-Methoxycitromycin	$C_{14}H_{12}O_5$	-6.5	2.267
11CFS & 11CQS	4-Hydroxyalternariol 9-methyl ether	$C_{15}H_{12}O_6$	-6.5	1.62
12CFS & 12CQS	Orthosporin	$C_{12}H_{12}O_5$	-6.5	1.25
13CFS & 13CQS	Altenusin	$C_{15}H_{14}O_6$	-6.4	2.356
	**Molecules Unique to the EFS**			
1UFS	4-Ethylcatechol	$C_8H_{10}O_2$	-6.4	13.571
2UFS	*p*-Coumaric acid	$C_9H_8O_3$	-6.7	0.563
3UFS	Diaportinol	$C_{13}H_{14}O_6$	-7	1.569
4UFS	Phenylacetic acid	$C_8H_8O_2$	-6.4	2.721
5UFS	Procyanidin dimer B1	$C_{30}H_{26}O_{12}$	-4.5	2.661
6UFS	Theaflavin	$C_{29}H_{24}O_{12}$	-4.23	4.48
	**Molecules Unique to the EFS**			

1UQS	6-O-desmethylterphenyllin	$C_{19}H_{16}O_5$	-6.9	1.443
2UQS	Altertoxin I	$C_{20}H_{16}O_6$	-7.8	4.518
3UQS	Altechromone B	$C_{14}H_{14}O_6$	-6.8	2.083
4UQS	Botryorhodine F	$C_{16}H_{14}O_6$	-6.7	1.521
5UQS	3',4',7-Trihydroxyisoflavanone	$C_{15}H_{12}O_5$	-7.5	1.811
6UQS	alternariol 9-methyl ether	$C_{15}H_{12}O_5$	-7	3.741
7UQS	Morin	$C_{15}H_{10}O_7$	-7.5	2.805

Supplementary file 3 (A): Docking studies were performed for twenty-six tentatively identified molecules from EFS and EQS using AutoDock Vina software with α-glucosidase (2QMJ).

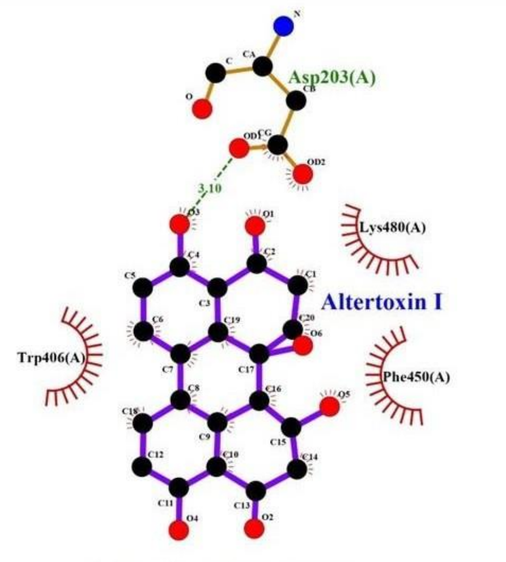

2QMJ_Altertoxin I

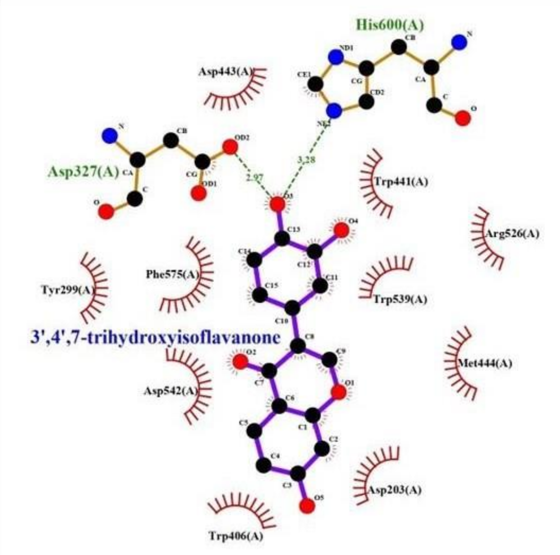

2QMJ_3',4',7-trihydroxyisoflavanone

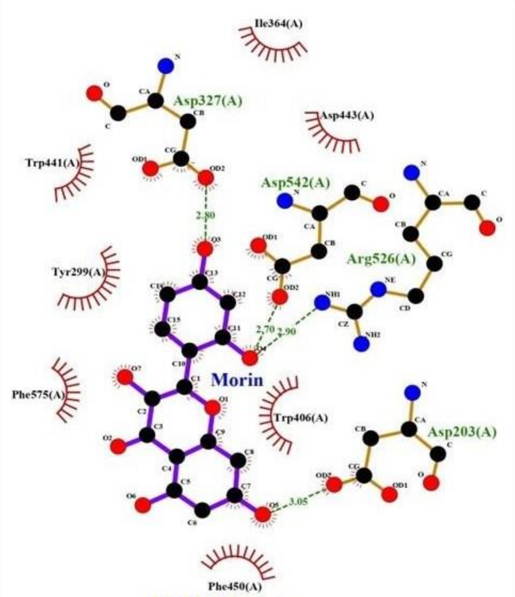

2QMJ_Morin

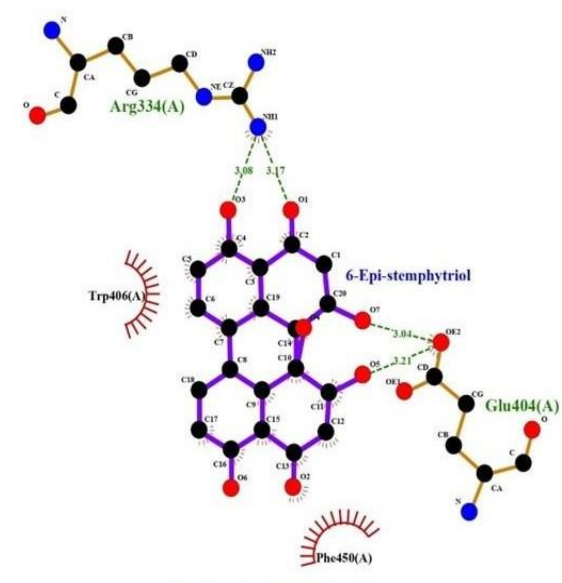

2QMJ_6-Epi-stemphytriol

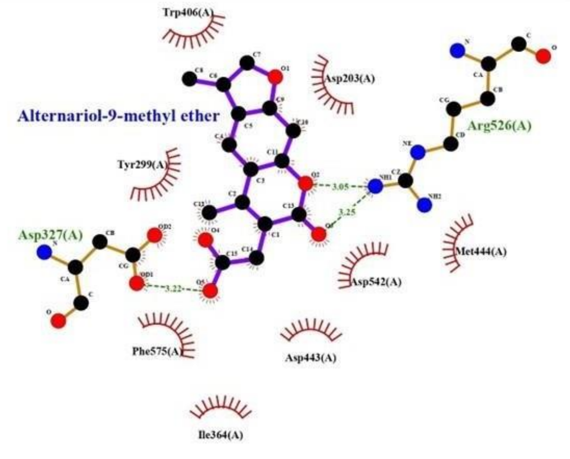

2QMJ_Alternariol-9-methyl ether

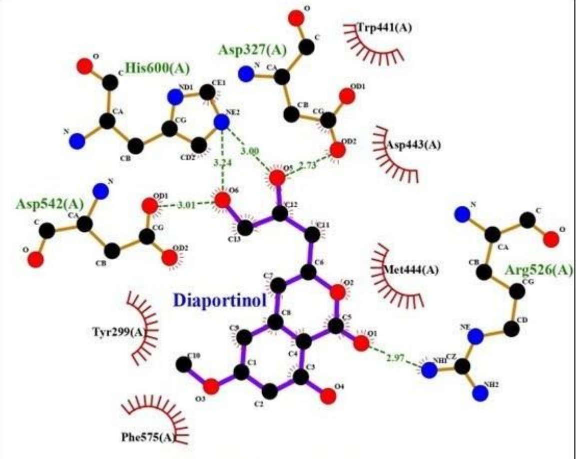

2QMJ_Diaportinol

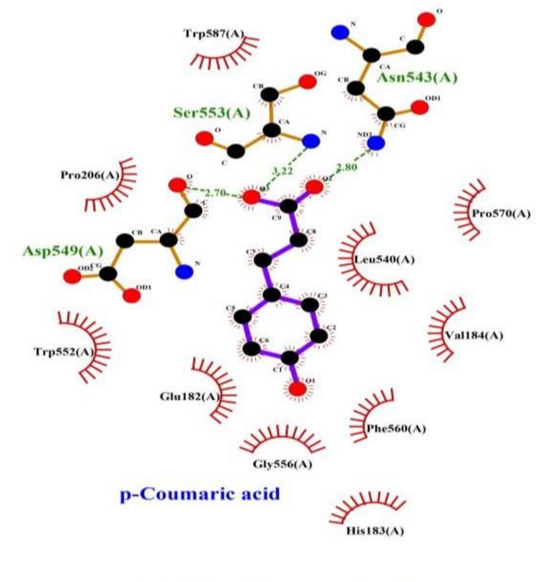

2QMJ_p-Coumaric acid

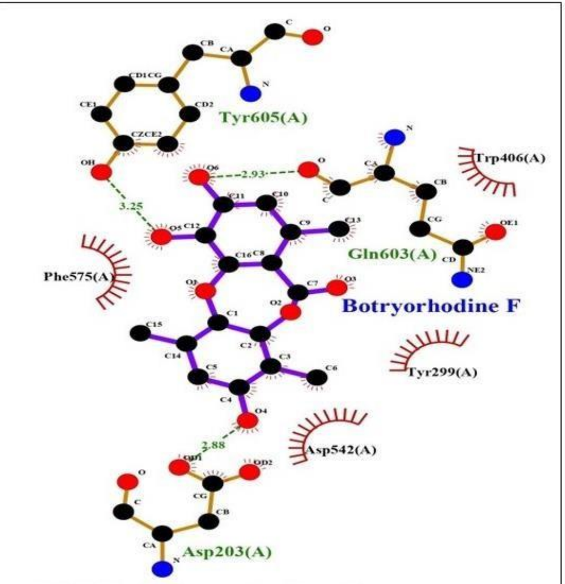

2QMJ_Botryorhodine F

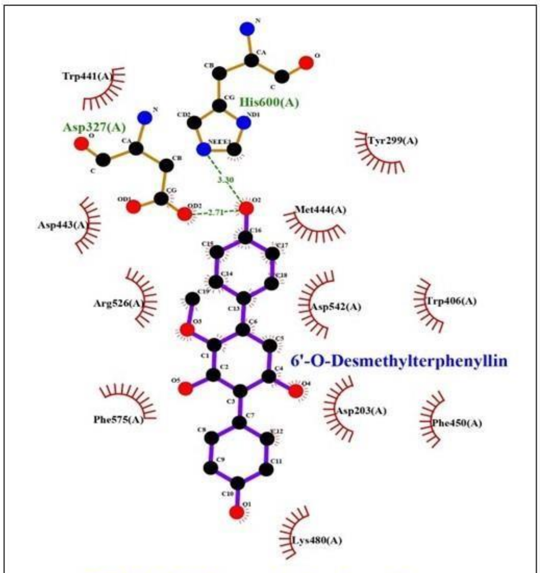

2QMJ_6'-O-Desmethylterphenyllin

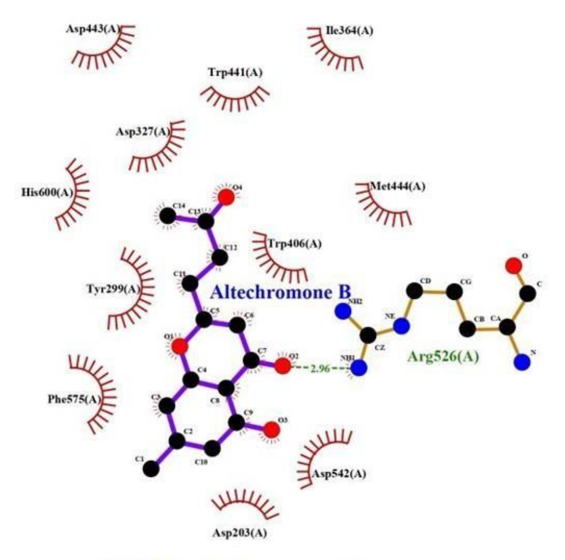

2QMJ_Altechromone B

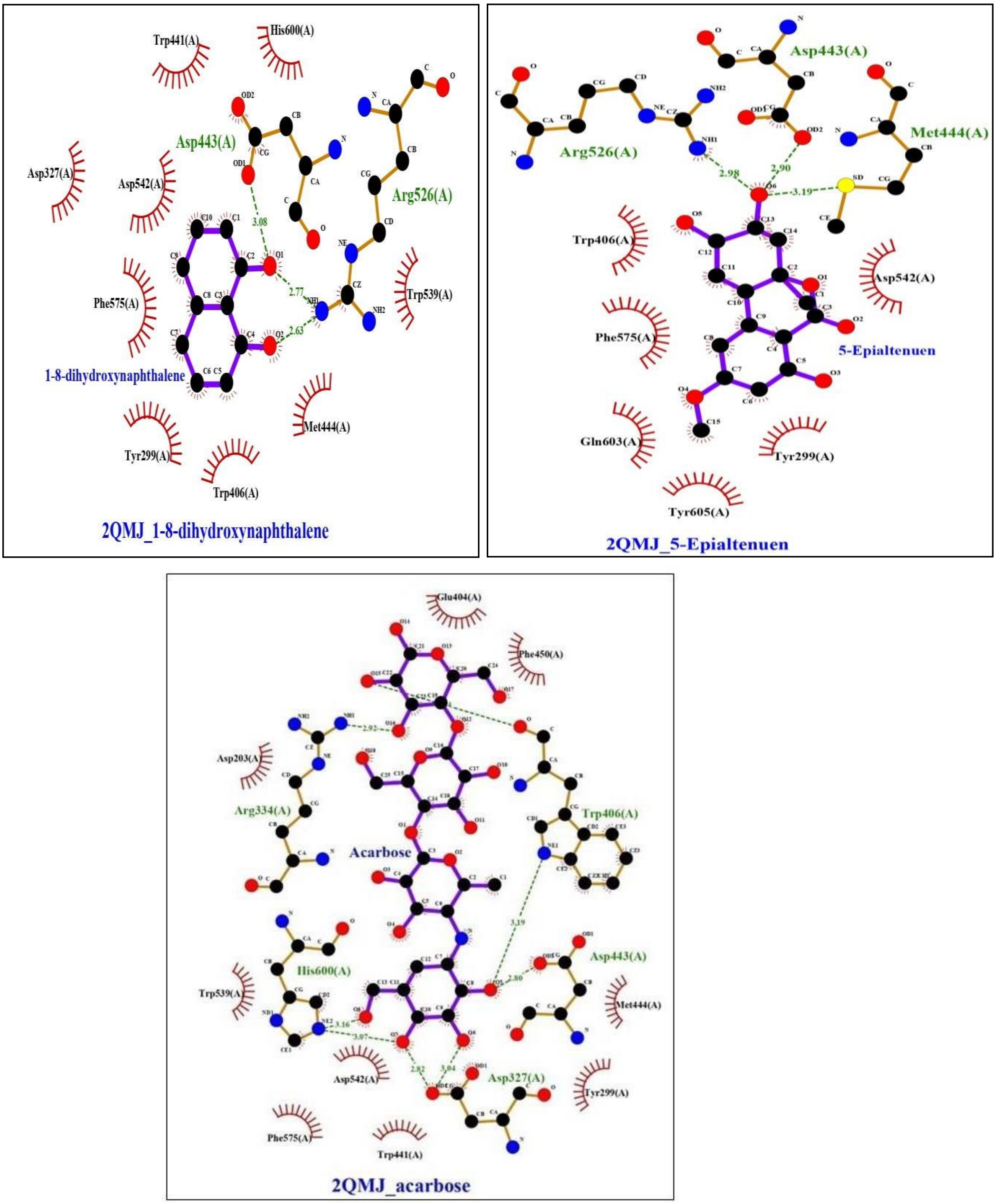

Supplementary file 3 (B). Twelve molecules identified from EFS and EQS exhibited greater binding affinities for the active site of α-glucosidase compared to acarbose.

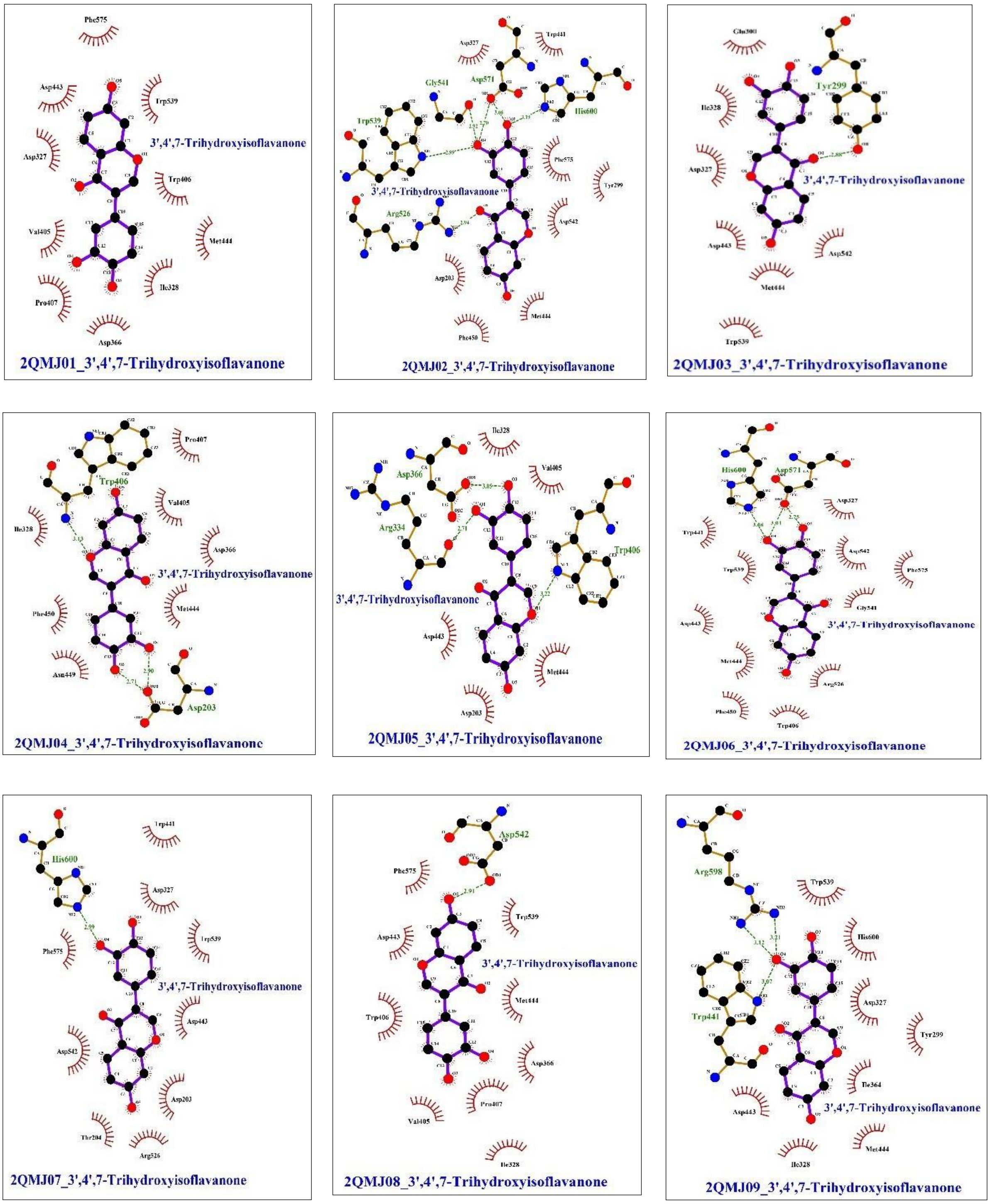

Supplementary file 3 (C). Ensemble docking of 3',4',7-Trihydroxyisoflavanone was plotted using the ligplot plus for the nine poses of a ligands bound to its 2QMJ protein.

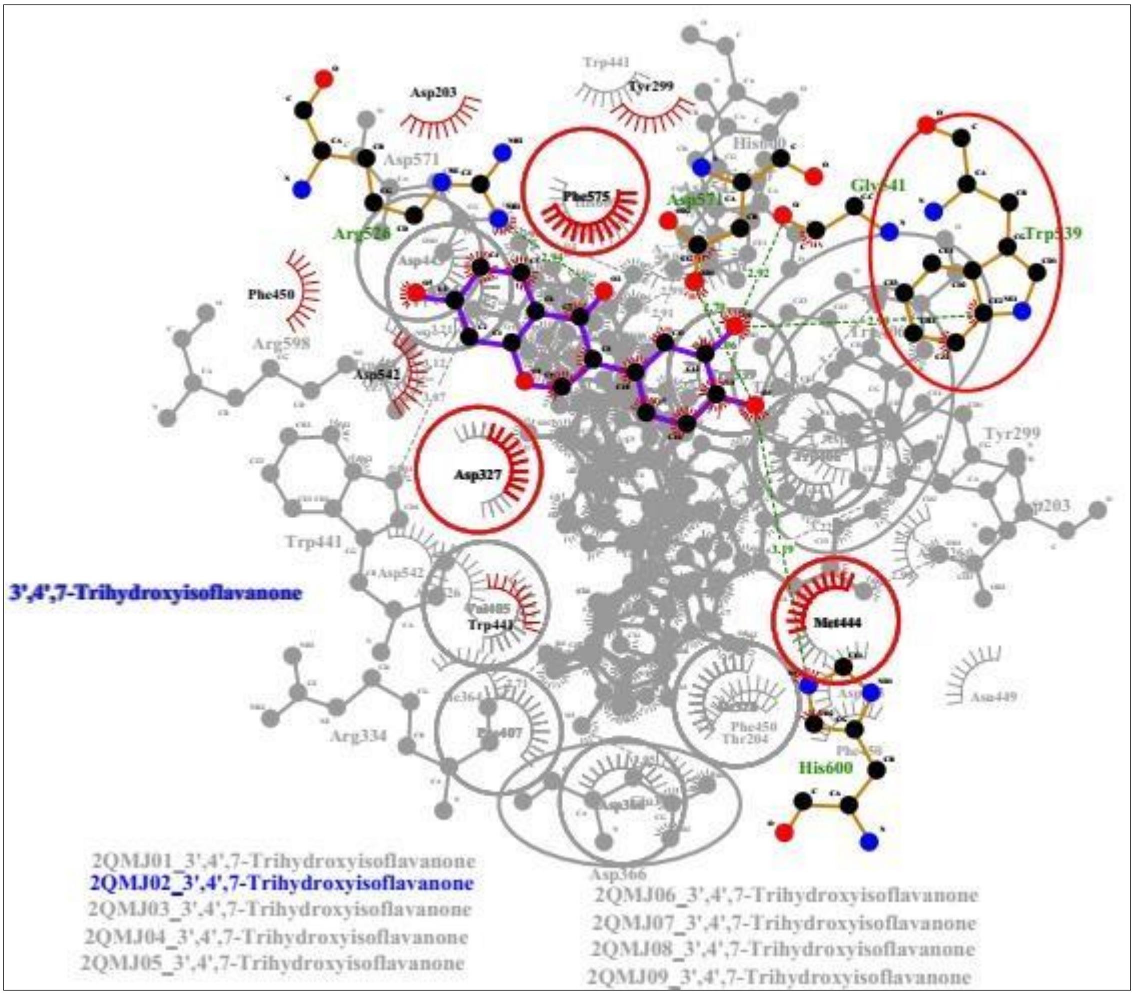

Supplementary file 3 (C). The overlapping of the docked complex of 3',4',7-Trihydroxyisoflavanone with nine poses of 2QMJ protein obtained from simulation.

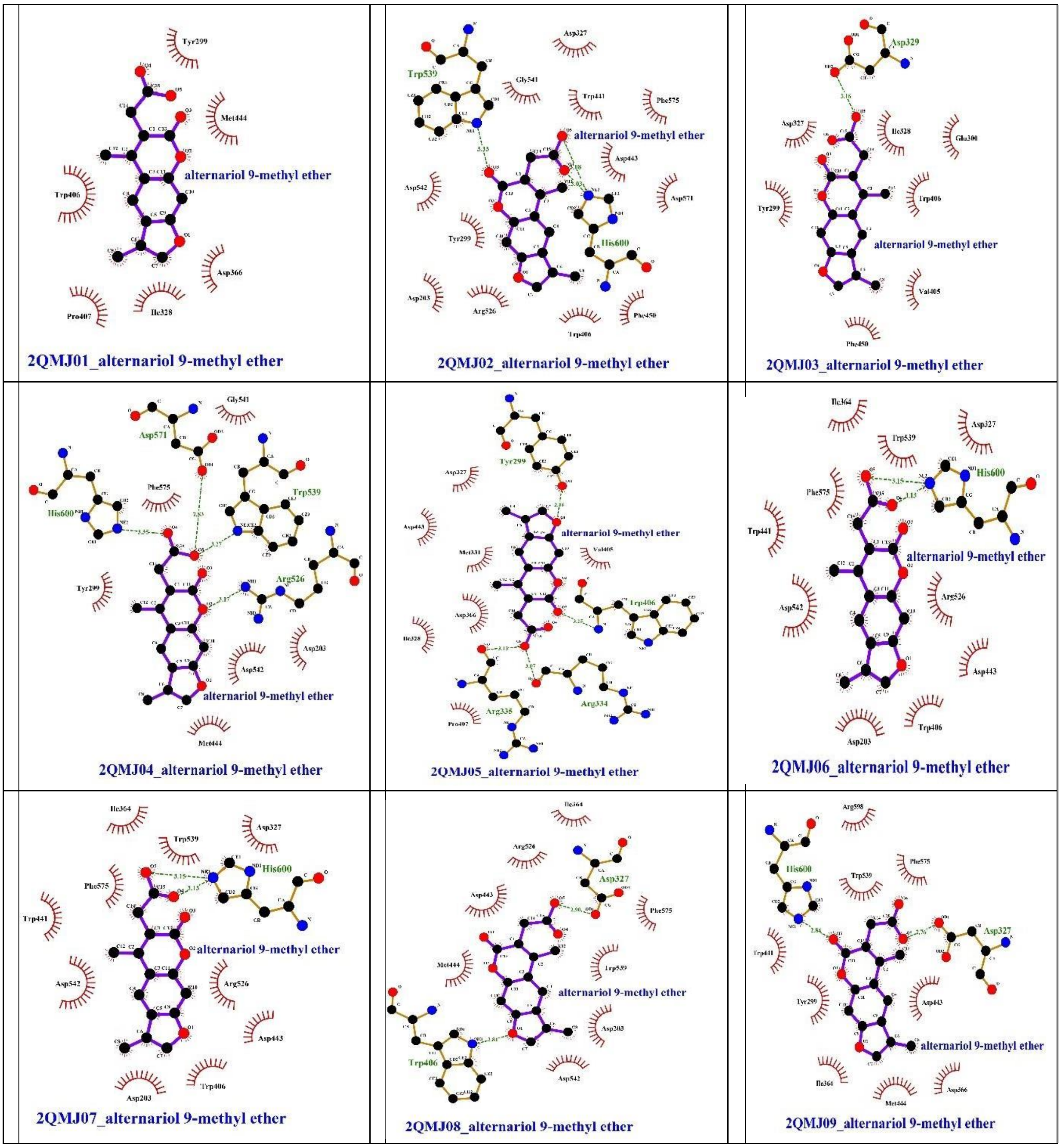

Supplementary file 3 (D). Ensemble docking of alternariol 9-methyl ether was plotted using ligplot plus for nine poses of a ligands bound to its 2QMJ protein.

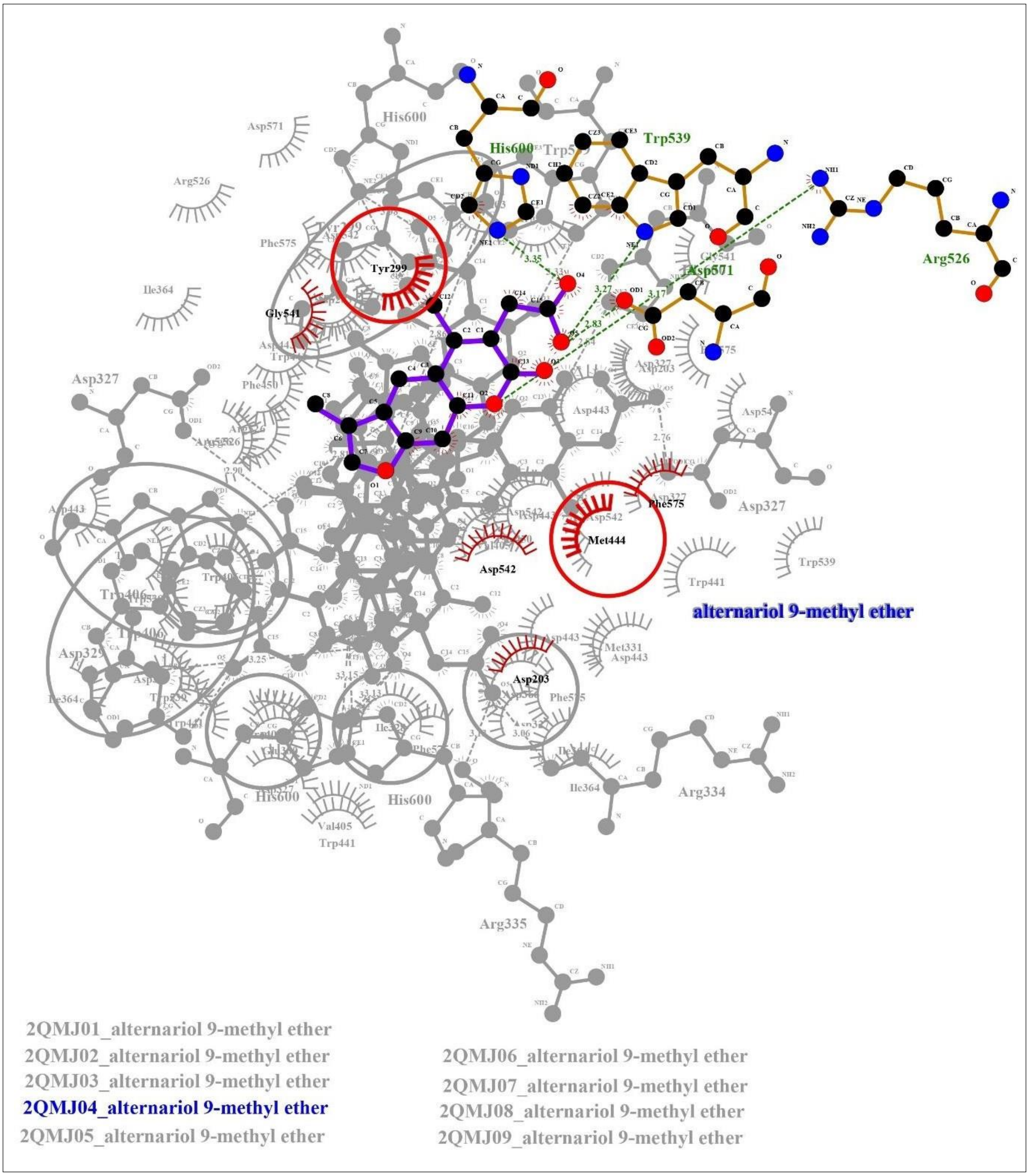

Supplementary file 3 (D). The overlapping of docked complex of alternariol 9-methyl ether with nine poses of protein obtained from simulation.

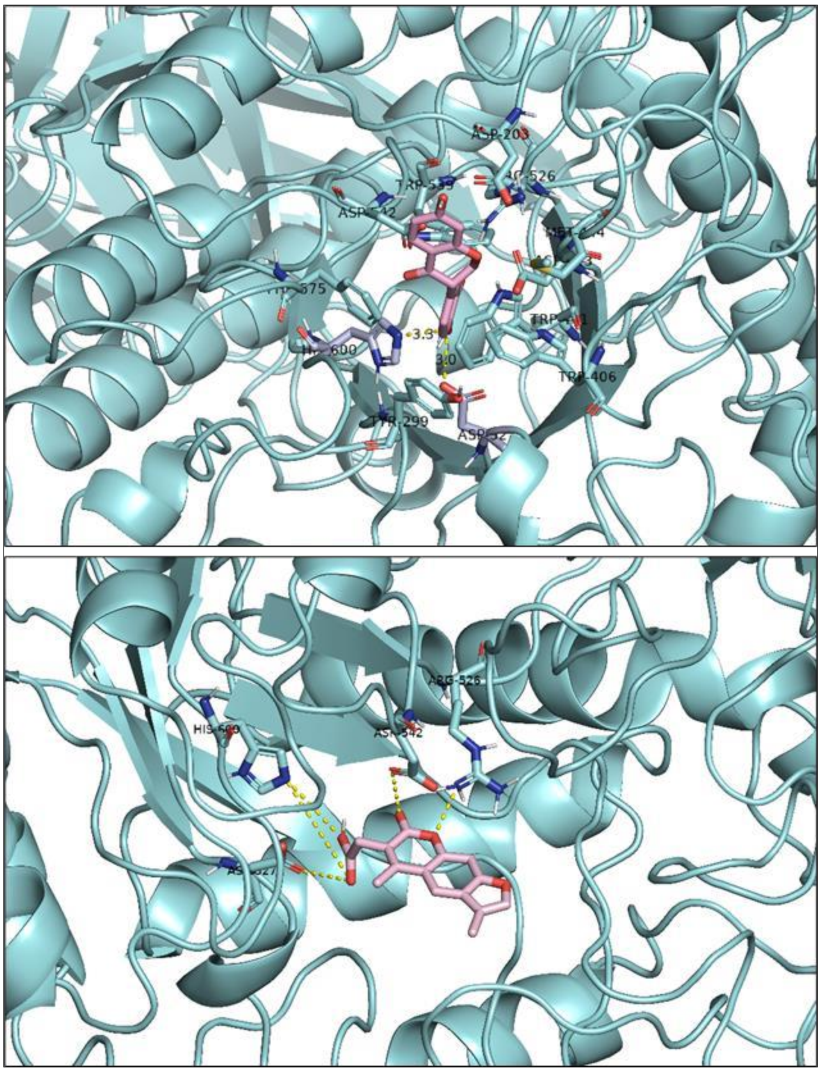

Supplementary file 3 (E). Docking of the 3',4',7-Trihydroxyisoflavanone (top) and alternariol 9-methyl ether (bottom). The figure was generated in the pymol software.
