# Supplementary material for: Elucidation of α-glucosidase inhibitory activity and UHPLC-ESI-QTOF-MS based metabolic profiling of endophytic fungi Alternaria alternata BRN05 isolated from seeds of Swietenia macrophylla king
Source: Front Fungal Biol. 2025 Jan 28;6:1447609. doi: 10.3389/ffunb.2025.1447609 (PMC11811940; doi:10.3389/ffunb.2025.1447609)

Supplementary File

- MS/MS spectra for the 39 compounds, along with fragments generated from Metfrag, as well as those analysed using ChemDraw Pro 8.0 software.

- In the Common Full-strength mode (CFS), MS/MS spectra of 13 molecules were generated

4-Hydroxymellein(1CFS) _12.953

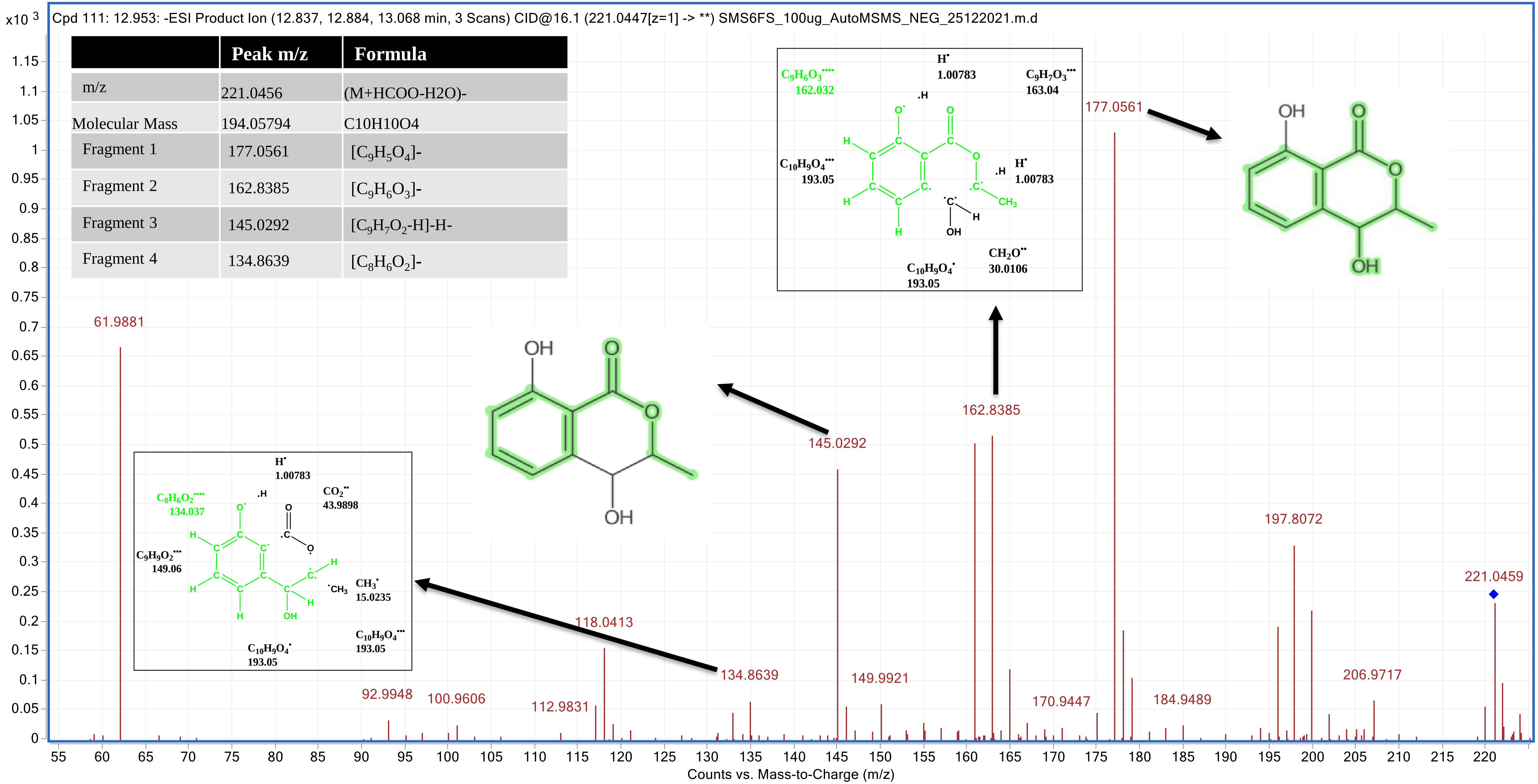

	Peak m/z	Formula
m/z	221.0456	(M+HCOO-H2O)-
Molecular Mass	194.05794	C10H10O4
Fragment 1	177.0561	$[C_9H_5O_4]^-$
Fragment 2	162.8385	$[C_9H_6O_3]^-$
Fragment 3	145.0292	$[C_9H_7O_2\text{-}H]\text{-}H^-$
Fragment 4	134.8639	$[C_8H_6O_2]^-$

5'-Epialtenuene (2CFS) _12.337

	Peak m/z	Formula
m/z	291.0874	(M-H)-
Molecular Mass	292.09474	$C_{15}H_{16}O_6$
Fragment 1	273.0776	$[C_{15}H_{13}O_5]^-$
Fragment 2	247.0984	$[C_{14}H_{15}O_4]^-$
Fragment 3	231.0665	$[C_{13}H_{11}O_4]^-$
Fragment 4	186.0715	$[C_{11}H_6O_3]^-$

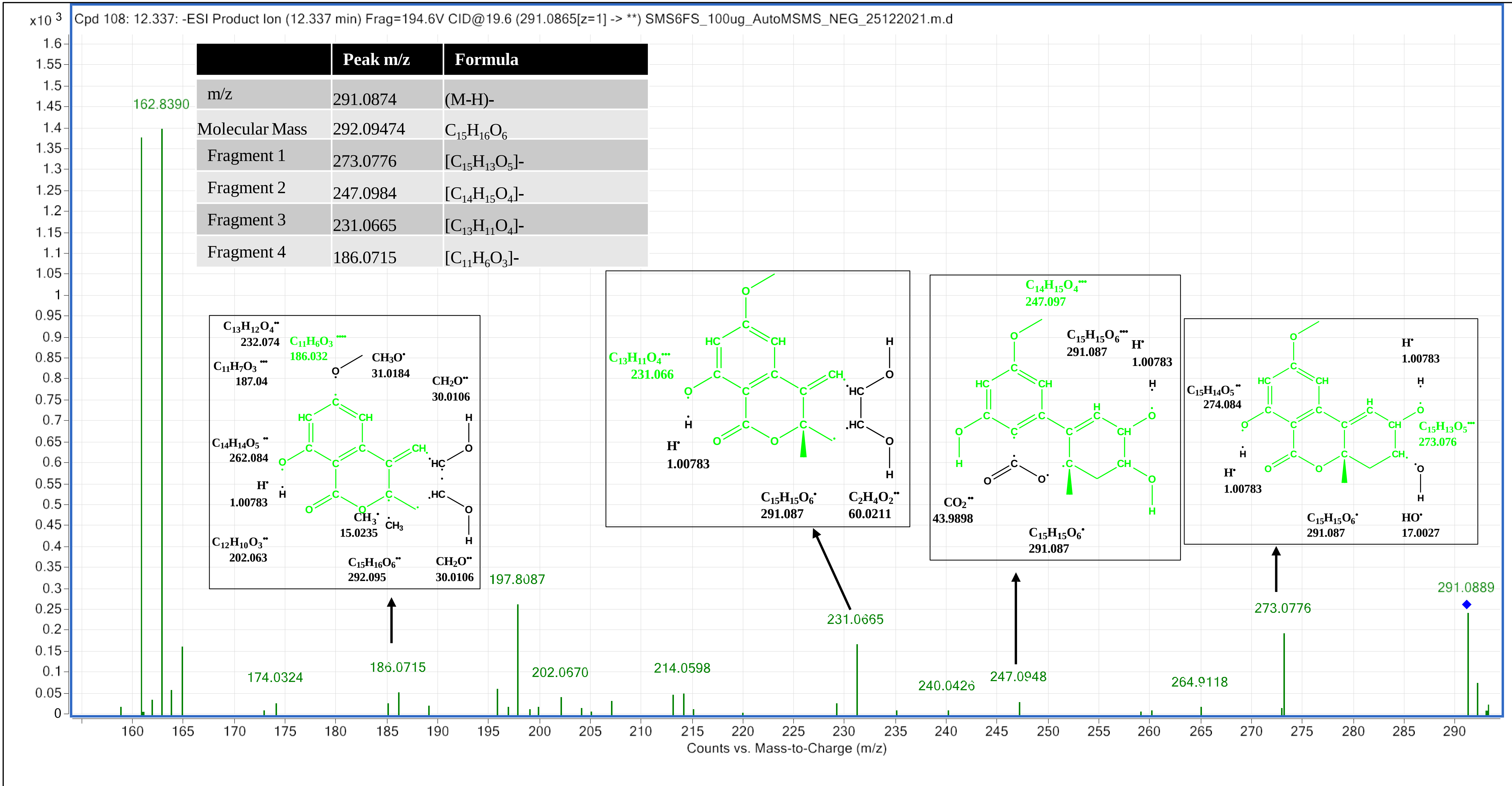

Alternarienonic acid (3CFS) _7.41

	Peak m/z	Formula
m/z	277.07908	(M-H)-
Molecular Mass	278.0792	$C_{14}H_{14}O_6$
Fragment 1	277.0716	$[C_{14}H_{12}O_6]^-$
Fragment 2	246.9260	$[C_{13}H_{10}O_5]^-$
Fragment 3	233.0813	$[C_{13}H_{13}O_4]^-$
Fragment 4	214.9257	$[C_{13}H_{10}O_3]^-$
Fragment 5	189.0542	$[C_{11}H_9O_3]^-$
Fragment 6	251.0563	$[C_{12}H_{11}O_6]^-$

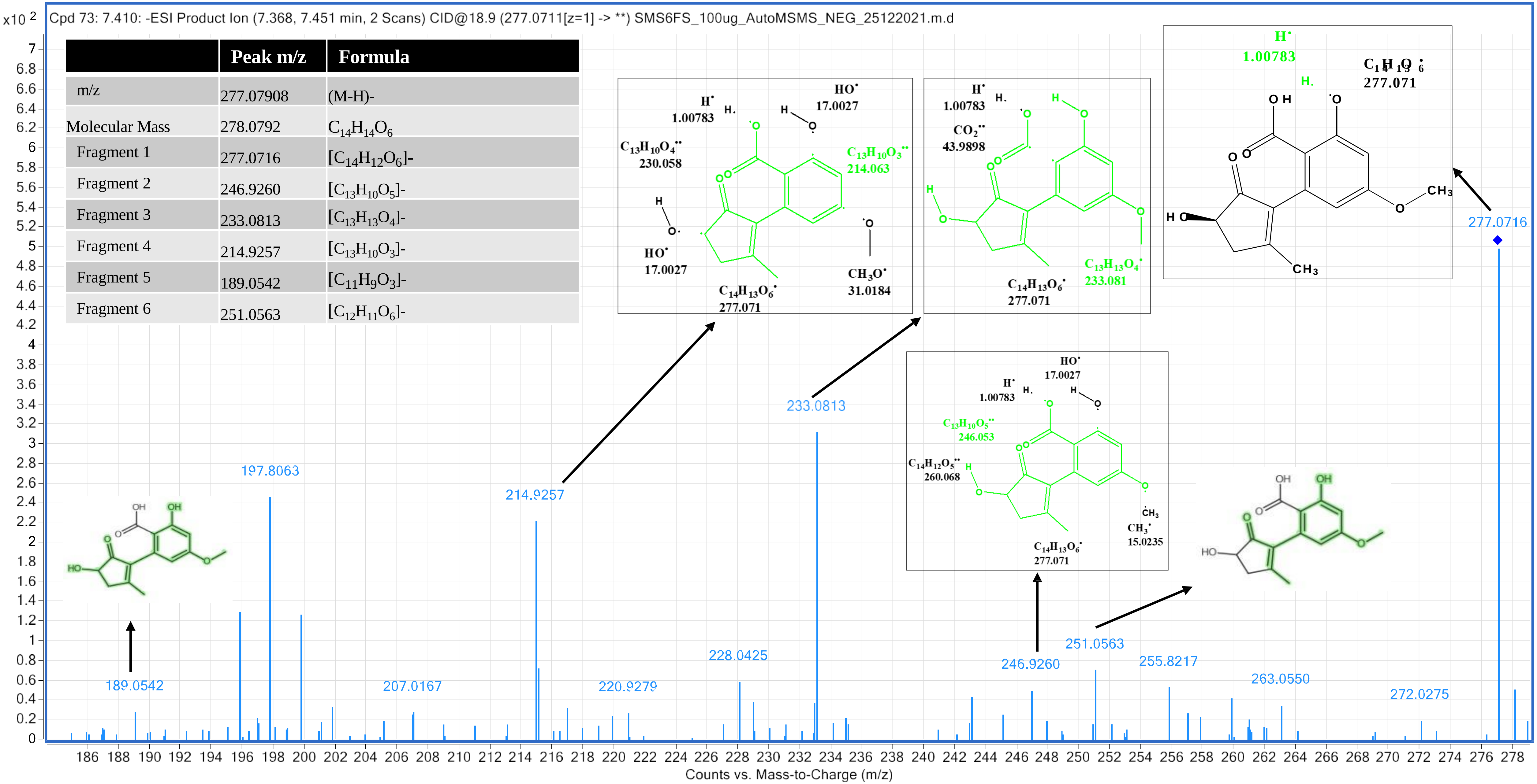

(+)-talaroflavone (4CFS) _15.758

	Peak m/z	Formula
m/z	257.0462	(M-H)- [-H_2O]
Molecular Mass	276.06342	$C_{14}H_{12}O_6$
Fragment 1	257.0461	[$C_{14}H_{10}O_5$]-
Fragment 2	246.9222	[$C_{13}H_{11}O_5$]-
Fragment 3	229.0516	[$C_{13}H_{11}O_4$]-
Fragment 4	213.0548	[$C_{13}H_{11}O_3$]-

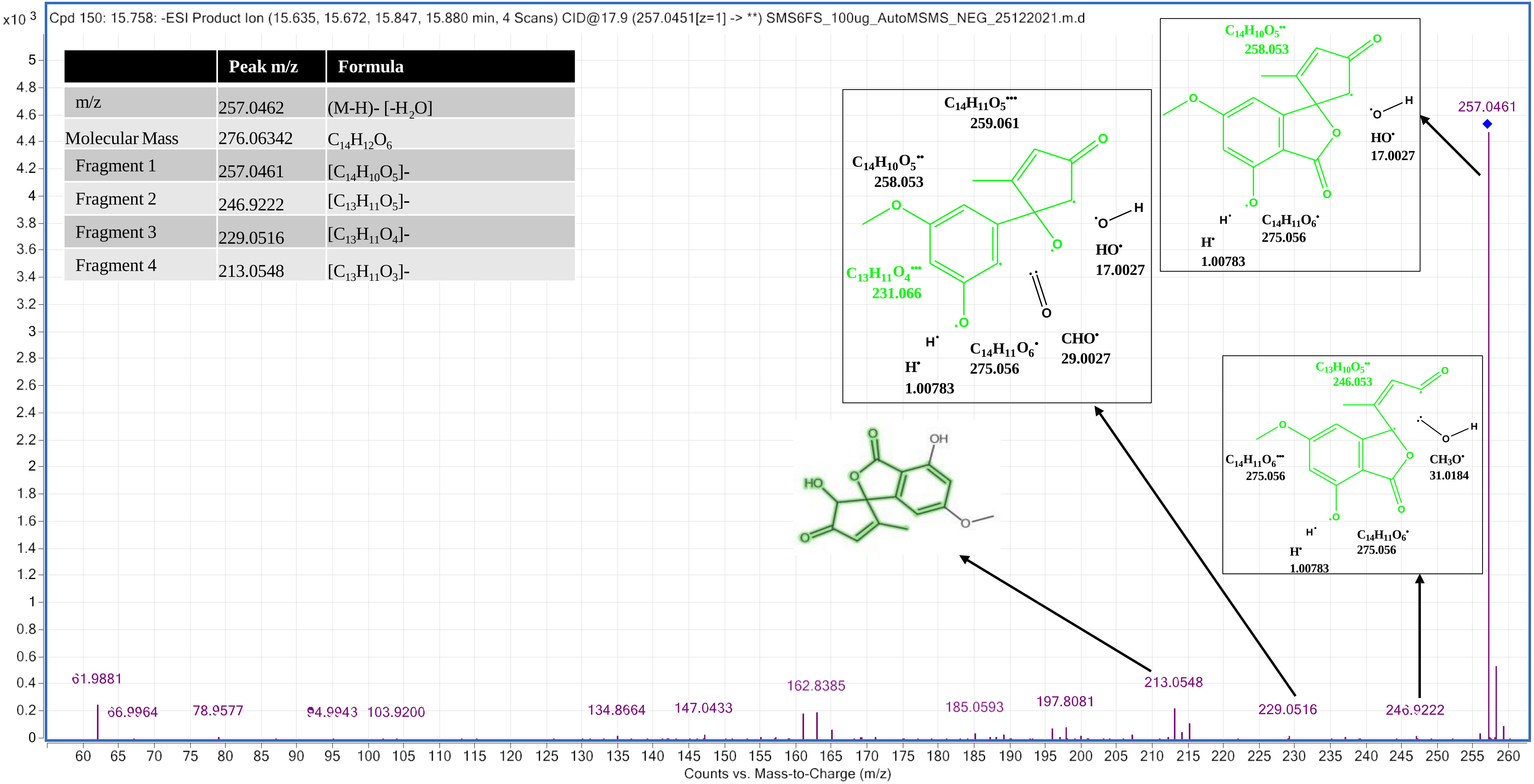

2,5-dimethyl-7-hydroxychromone (5CFS) _10.019

	Peak m/z	Formula
m/z	189.0567	(M-H)-
Molecular Mass	190.06303	$C_{11}H_{10}O_3$
Fragment 1	189.0567	$[C_{11}H_9O_3]$-
Fragment 2	174.0324	$[C_{10}H_6O_3]$-
Fragment 3	160.08424	$[C_9H_4O_3]$-
Fragment 4	119.0491	$[C_7H_3O_2]$-
Fragment 5	133.0646	$[C_9H_8O_2$ +H]-

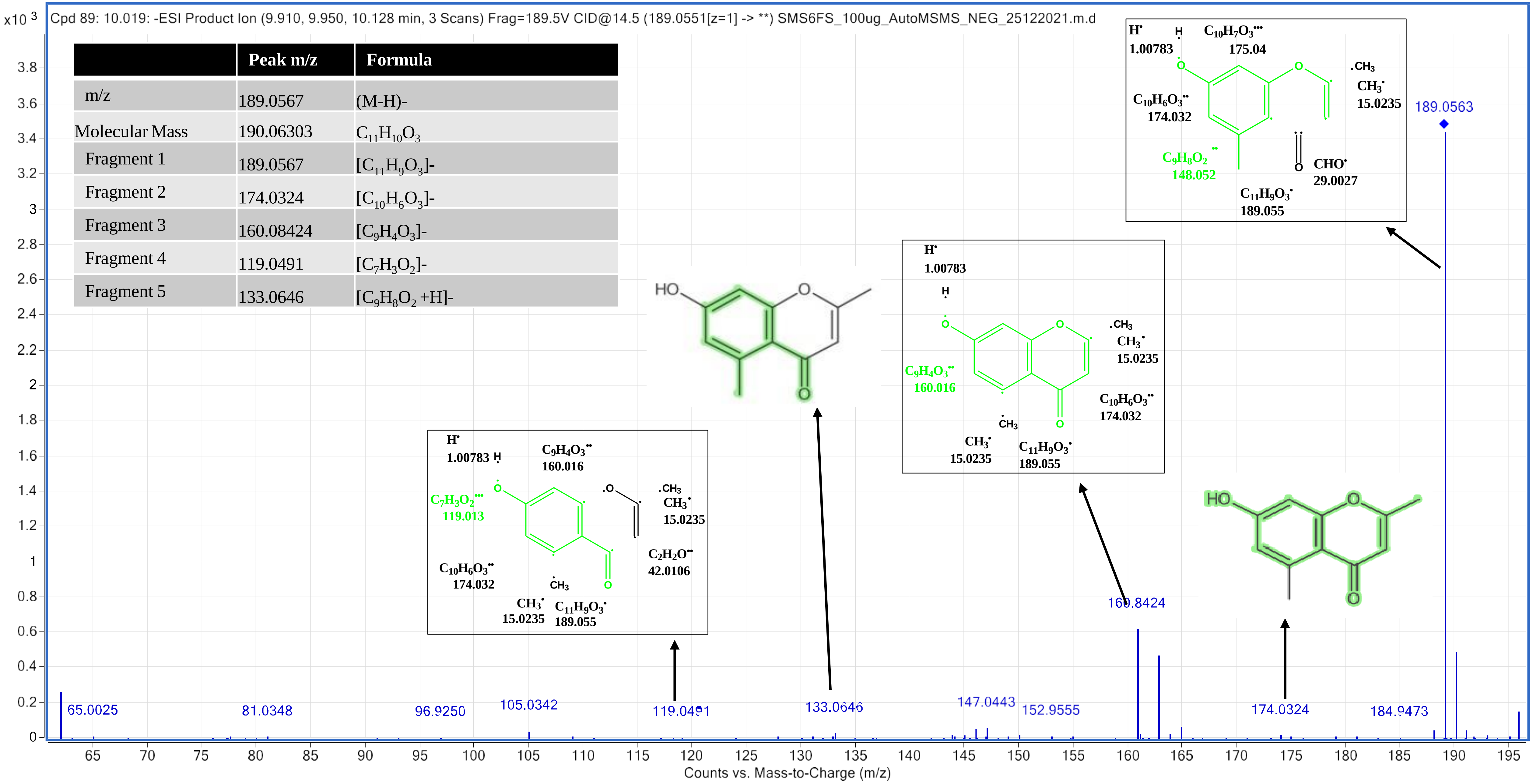

Alternarian acid (6CFS) _10.752

	Peak m/z	Formula
m/z	319.0464	(M-H)-
Molecular Mass	320.05324	$C_{15}H_{12}O_8$
Fragment 1	231.0659	$[C_{13}H_{10}O_4+H]^-$
Fragment 2	203.0699	$[C_{12}H_{10}O_3+H]^-$
Fragment 3	188.0467	$[C_{11}H_9O_3]$-H-
Fragment 4	160.8409	$[C_{10}H_9O_2]$-H-

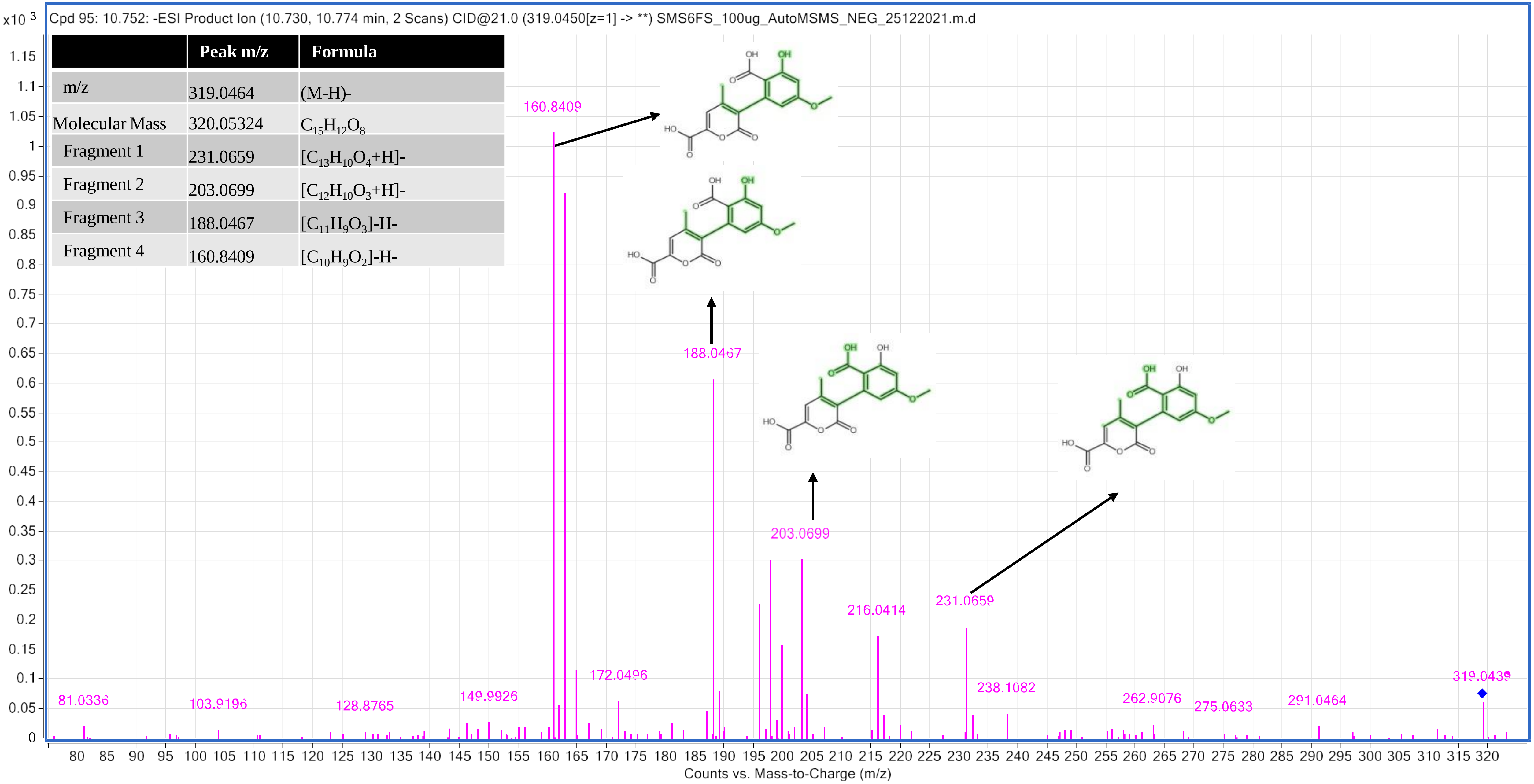

1,8-dihydroxynaphthalene (7CFS) _6.536

	Peak m/z	Formula
m/z	205.504	(M+HCOO)-
Molecular Mass	160.05246	$C_{10}H_8O_2$
Fragment 1	118.9931	$[C_8H_6O]^-$
Fragment 2	75.0032	$[C_6H_3O]^-$
Fragment 3	149.0256	$[C_9H_8O_2]^-$
Fragment 4	105.0338	$[C_7H_5O]^-$

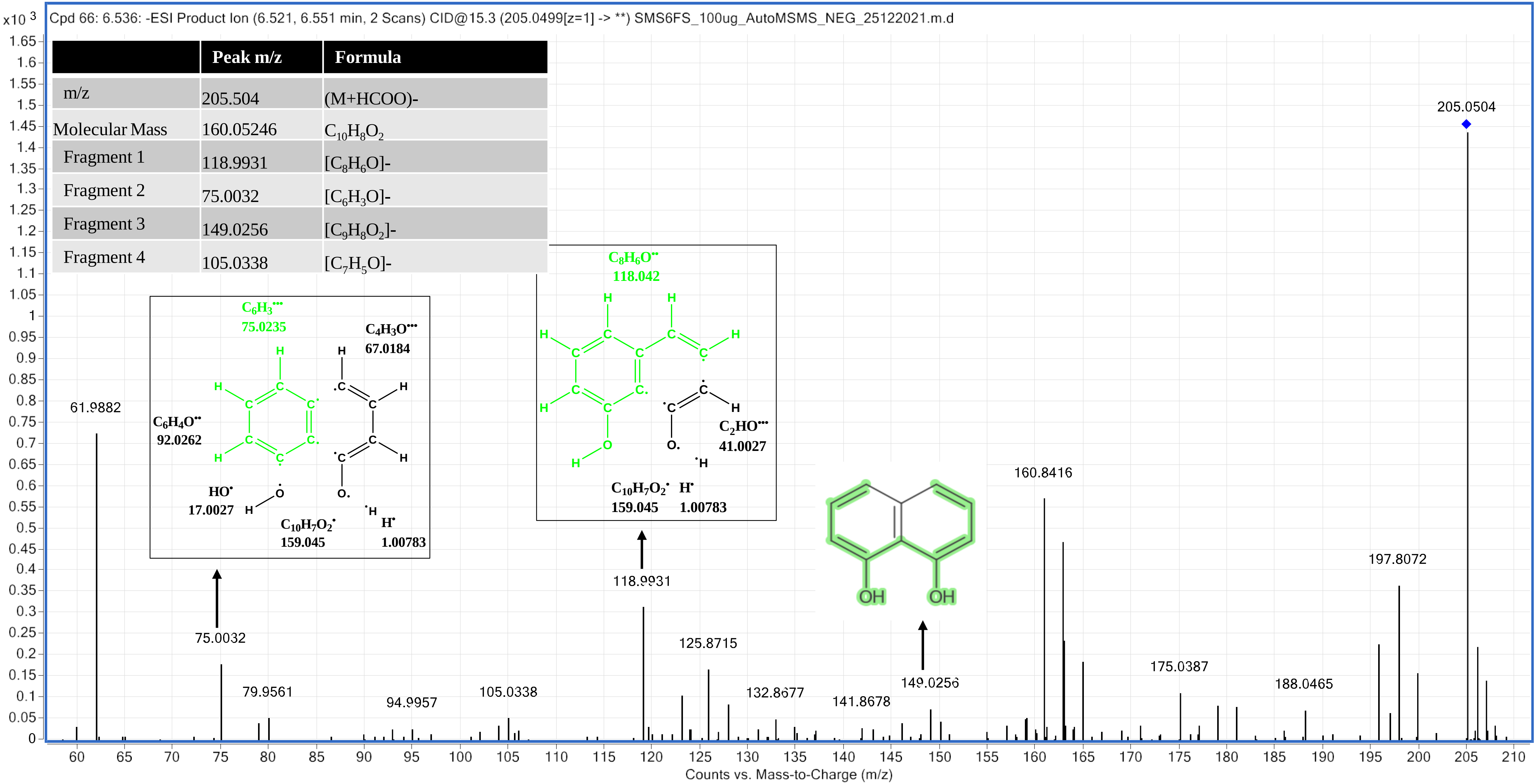

Aspergone Q (8CFS) _11.941

	Peak m/z	Formula
m/z	223.0609	(M-H)- [-H_2O]
Molecular Mass	242.07908	$C_{11}H_{14}O_6$
Fragment 1	223.0606	[$C_{11}H_{13}O_5$]-
Fragment 2	191.0347	[$C_{10}H_{10}O_4$-2H]-H-
Fragment 3	162.0319	[$C_9H_9O_3$-2H]-H-
Fragment 4	122.0383	[$C_7H_9O_2$-2H]-H-

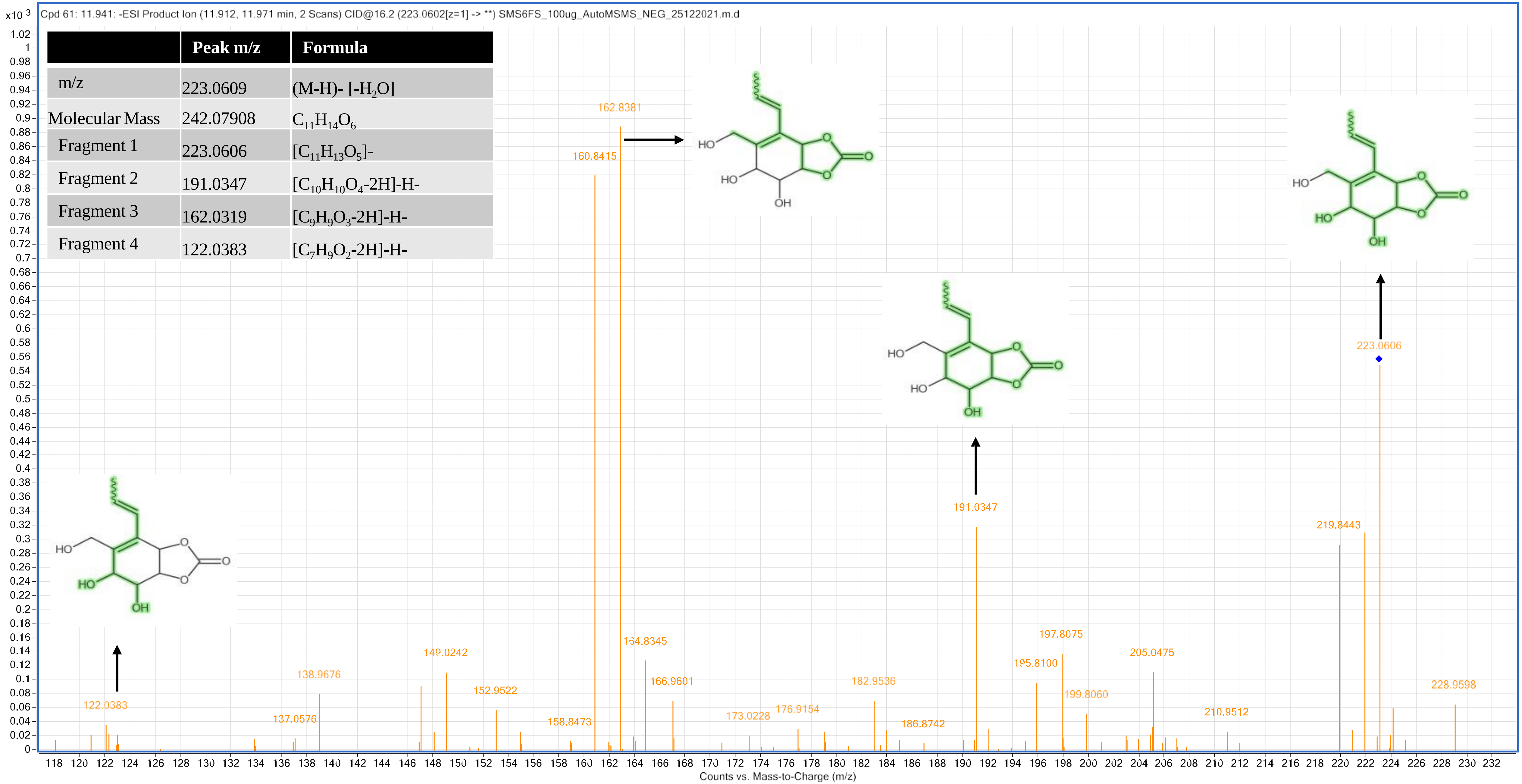

6-Epi-stemphytriol (9CFS) _12.968

	Peak m/z	Formula
m/z	349.0709	(M-H)- [-H_2O]
Molecular Mass	368.08965	$C_{20}H_{16}O_7$
Fragment 1	349.0701	[$C_{20}H_{15}O_6$-H]-H-
Fragment 2	331.06	[$C_{20}H_{14}O_5$-2H]-H-
Fragment 3	145.029	[$C_9H_5O_2$]-
Fragment 4	117.0352	[C_8H_4O+H]-

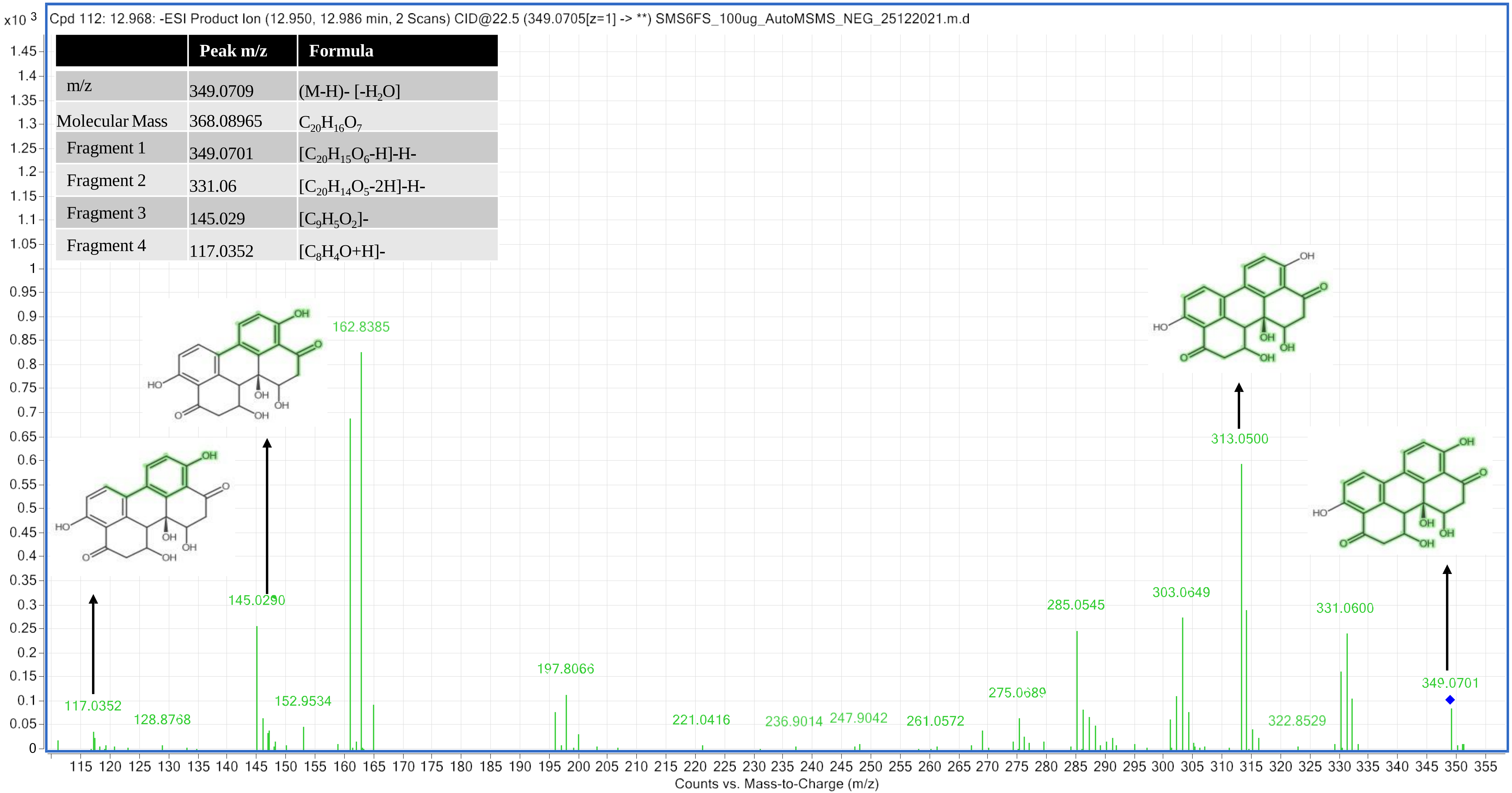

12-Methoxycitromycin (10CFS) _13.204

	Peak m/z	Formula
m/z	287.0568	(M+HCOO)-
Molecular Mass	260.06851	$C_{14}H_{12}O_5$
Fragment 1	229.05	$[[C_{13}H_9O_4]-$
Fragment 2	228.0424	$[C_{13}H_9O_4]-$
Fragment 3	200.0476	$[C_{12}H_8O_4]-$
Fragment 4	211.0402	$[C_{13}H_8O_3]-H-$
Fragment 5	243.06632	$[C_{14}H_{11}O_4]-$

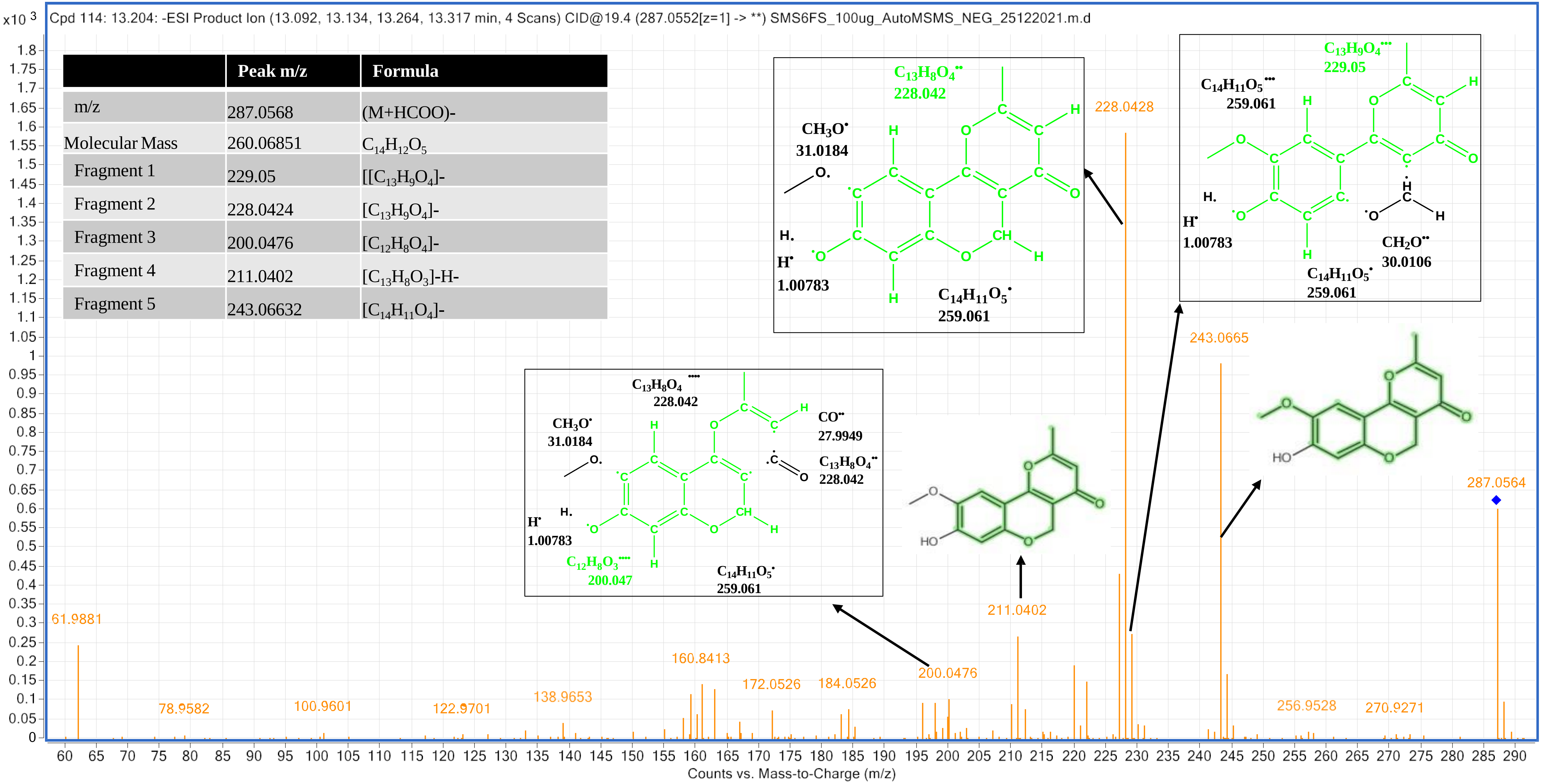

4-Hydroxyalternariol 9-methyl ether (11CFS) _13.532

	Peak m/z	Formula
m/z	287.0565	(M-H)-
Molecular Mass	288.06342	$C_{15}H_{12}O_6$
Fragment 1	271.0609	$[C_{15}H_{11}O_5]^-$
Fragment 2	245.0826	$[C_{13}H_9O_5]^-$
Fragment 3	230.0586	$[C_{13}H_{10}O_4]^-$
Fragment 4	160.0527	$[C_{10}H_9O_2]^-$
Fragment 5	202.0631	$[C_{12}H_{11}O_3]^-$

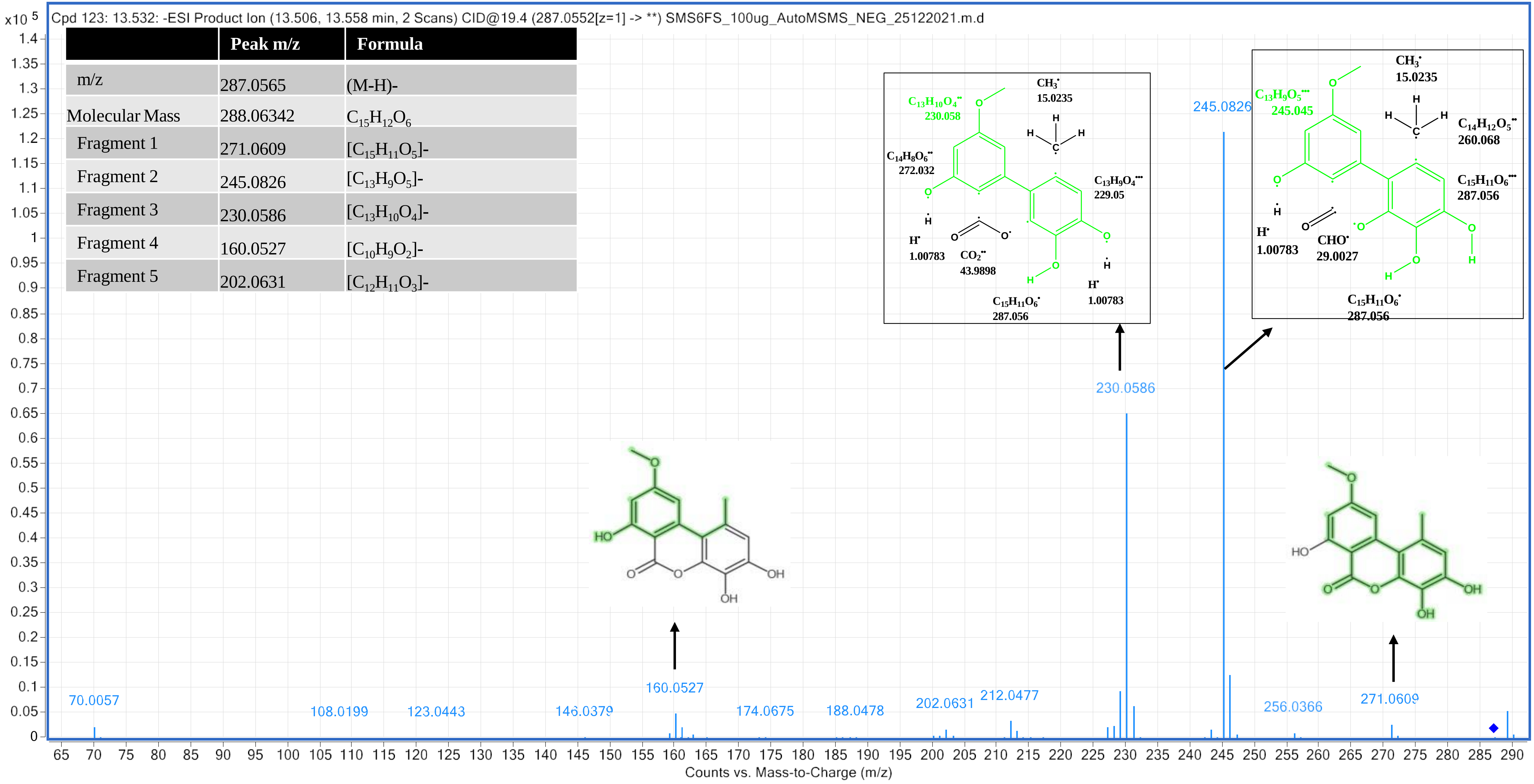

Orthosporin (12CFS) _15.00

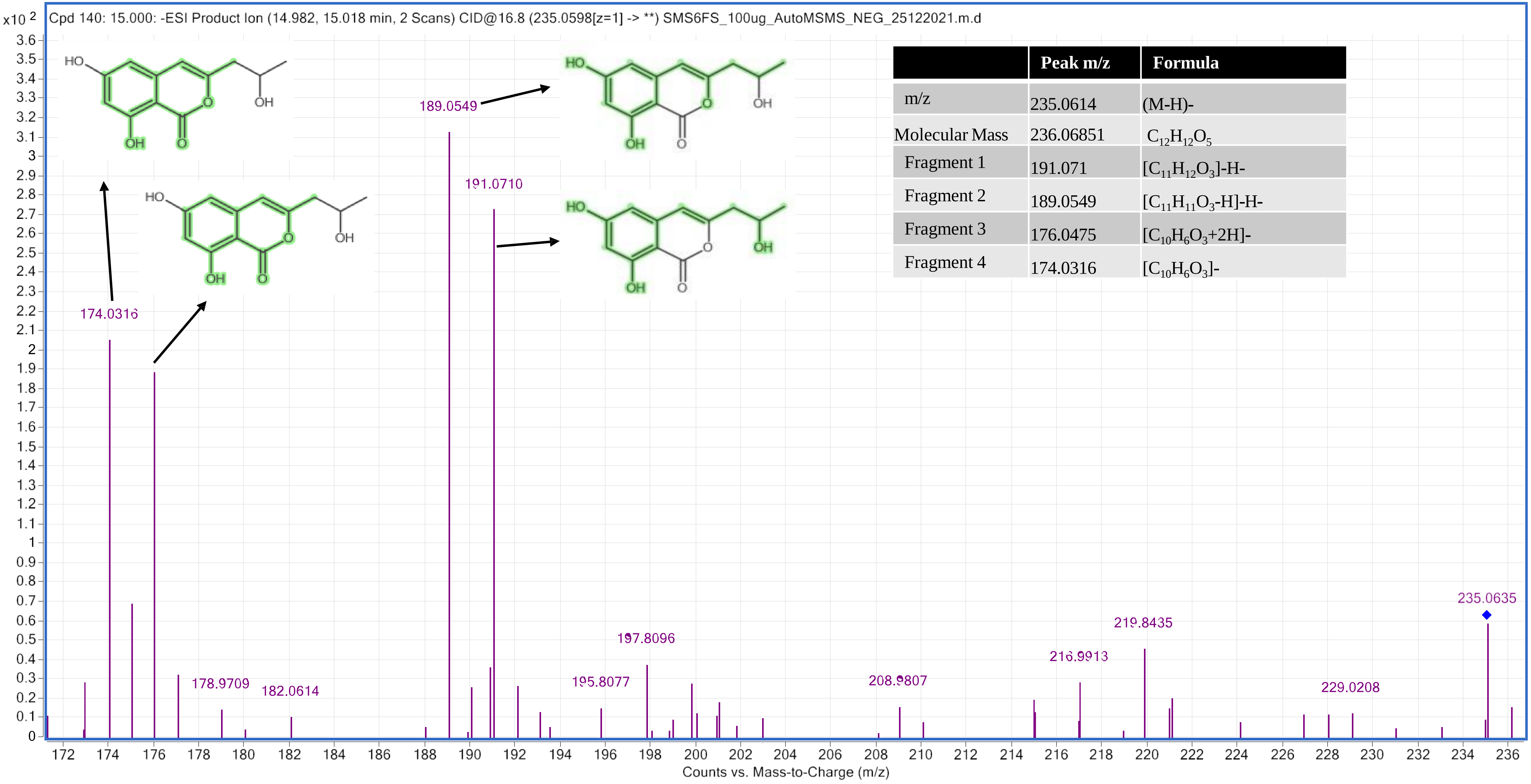

	Peak m/z	Formula
m/z	235.0614	(M-H)-
Molecular Mass	236.06851	$C_{12}H_{12}O_5$
Fragment 1	191.071	$[C_{11}H_{12}O_3]$-H-
Fragment 2	189.0549	$[C_{11}H_{11}O_3$-H]-H-
Fragment 3	176.0475	$[C_{10}H_6O_3$+2H]-
Fragment 4	174.0316	$[C_{10}H_6O_3]$-

Altenusin (13CFS) _19.829

	Peak m/z	Formula
m/z	317.0657	(M+HCOO)- [-H_2O]
Molecular Mass	290.07908	$C_{15}H_{14}O_6$
Fragment 1	272.068	[$C_{15}H_{12}O_5$]-
Fragment 2	271.016	[$C_{15}H_{11}O_5$]-
Fragment 3	270.0149	[$C_{15}H_{10}O_5$]-
Fragment 4	242.0223	[$C_{14}H_{10}O_4$]-
Fragment 5	198.0313	[$C_{13}H_{10}O_2$]-

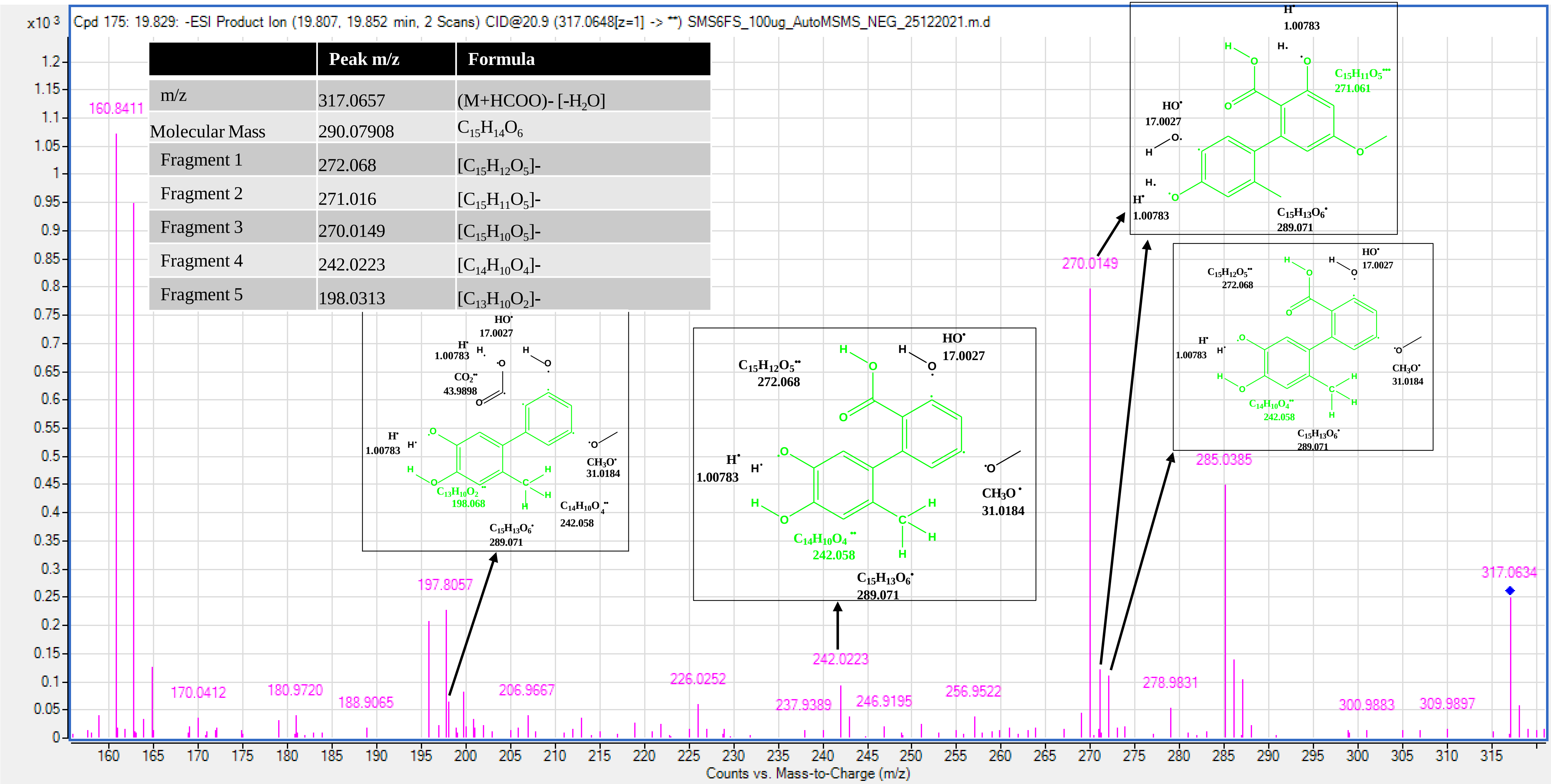

In the Unique Full-strength mode (UFS), MS/MS spectra of 6 molecules were generated

4-Ethylcatechol (1UFS) _5.595

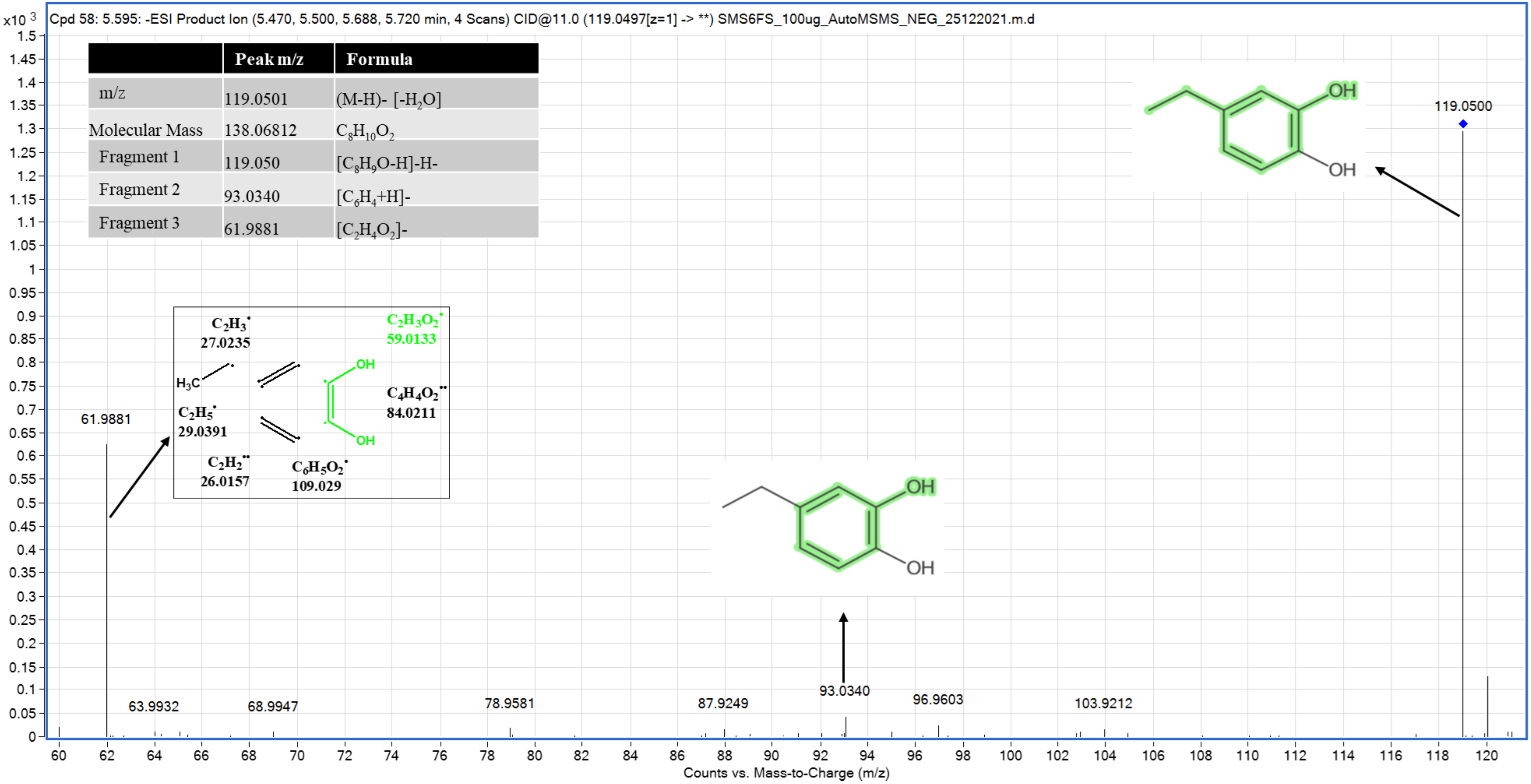

	Peak m/z	Formula
m/z	119.0501	(M-H)- [-H_2O]
Molecular Mass	138.06812	$C_8H_{10}O_2$
Fragment 1	119.050	[C_8H_9O-H]-H-
Fragment 2	93.0340	[C_6H_4+H]-
Fragment 3	61.9881	[$C_2H_4O_2$]-

p-Coumaric acid (2UFS) _7.023

	Peak m/z	Formula
m/z	209.0458	(M+HCOO)-
Molecular Mass	164.04737	$C_9H_8O_3$
Fragment 1	123.0453	$[C_7H_7O_2]^-$
Fragment 2	118.9625	$[C_5H_6O]^-$

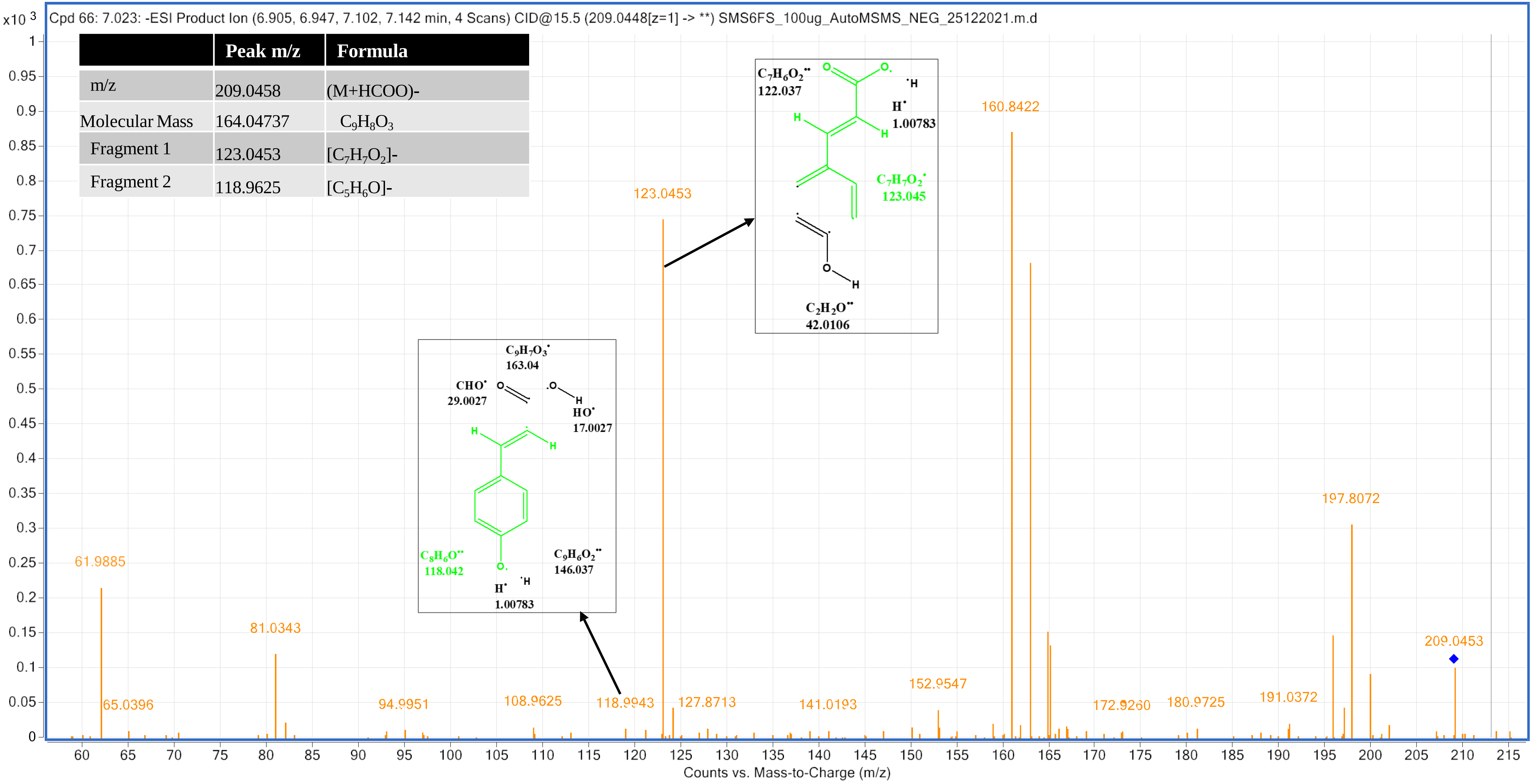

Diaportinol (3UFS) _9.382

	Peak m/z	Formula
m/z	247.0613	((M-H)- [-H_2O]
Molecular Mass	266.07908	$C_{13}H_{14}O_6$
Fragment 1	219.8431	$[C_{12}H_{11}O_4]^-$
Fragment 2	210.9479	$[C_{10}H_{10}O_4]^-$
Fragment 3	203.0703	$[C_{12}H_{11}O_3]^-$
Fragment 4	166.9587	$[C_9H_{10}O_3]^-$

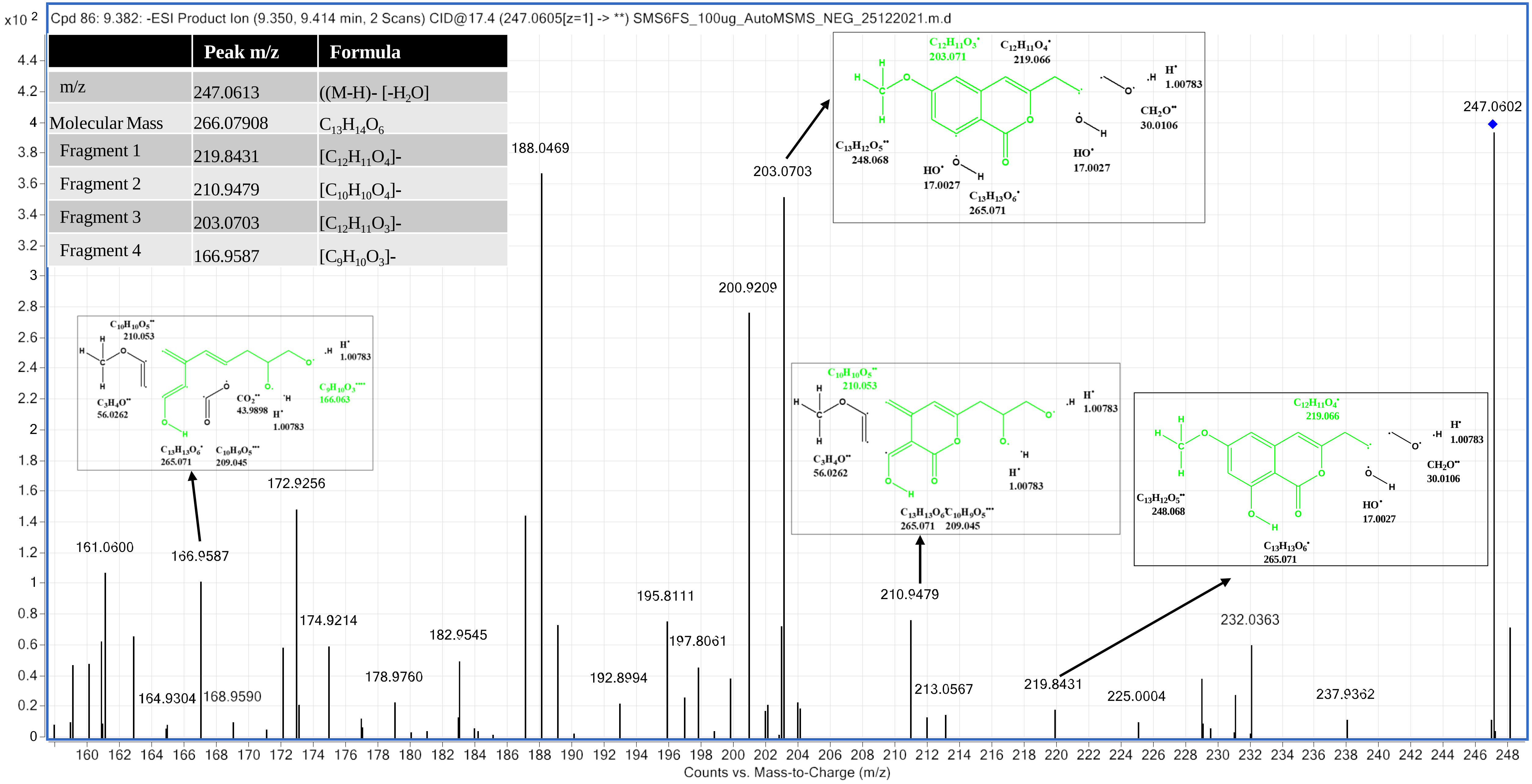

Phenylacetic acid (4UFS) _5.707

	Peak m/z	Formula
m/z	163.04	(M+HCOO)-[-H_2O]
Molecular Mass	136.05246	$C_8H_8O_2$
Fragment 1	119.0504	[C_8H_7O]-
Fragment 2	91.0549	[C_7H_7]-
Fragment 3	63.9965	[C_4H_3O-2H]-H-

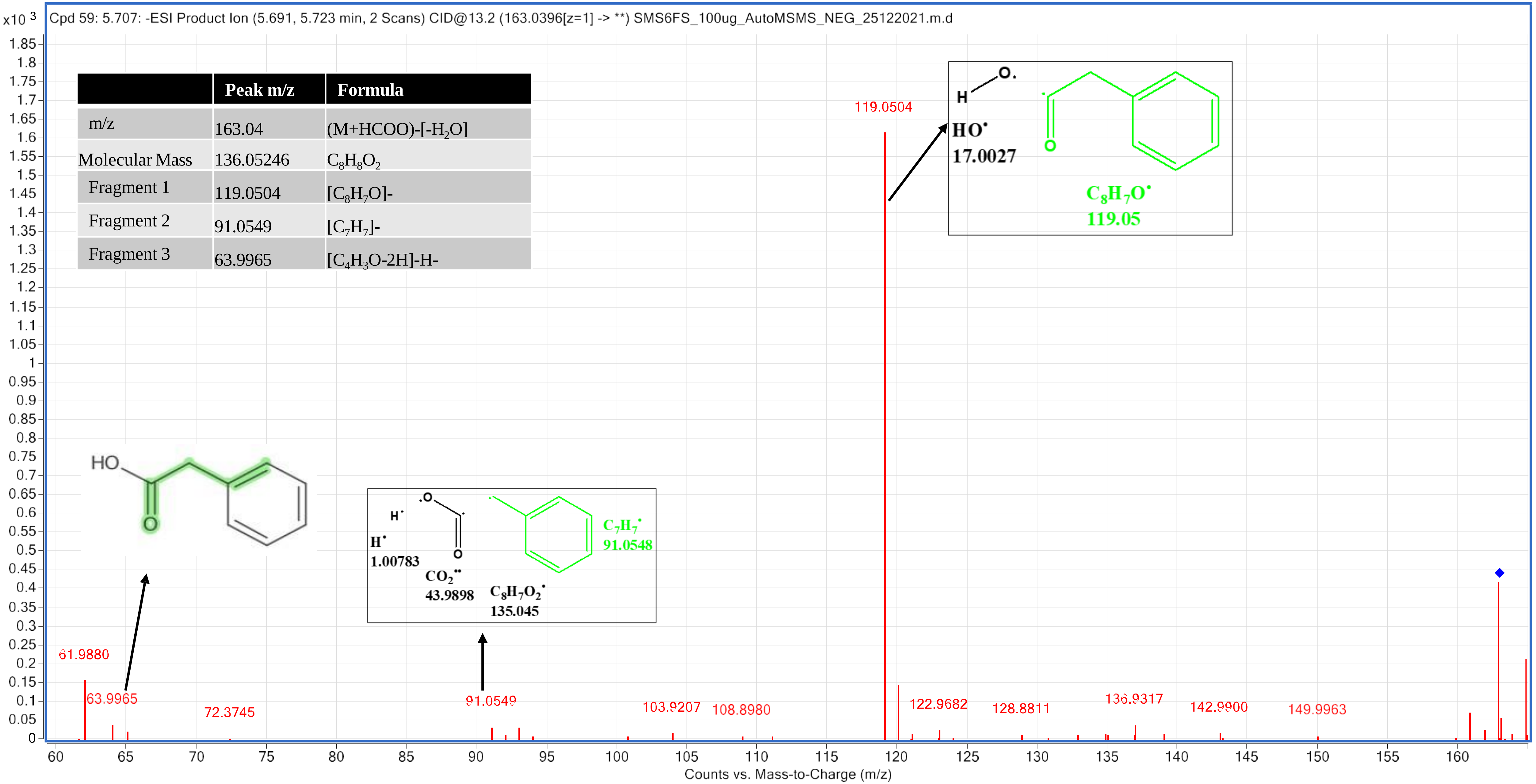

Procyanidin dimer B1 (5UFS) _16.753

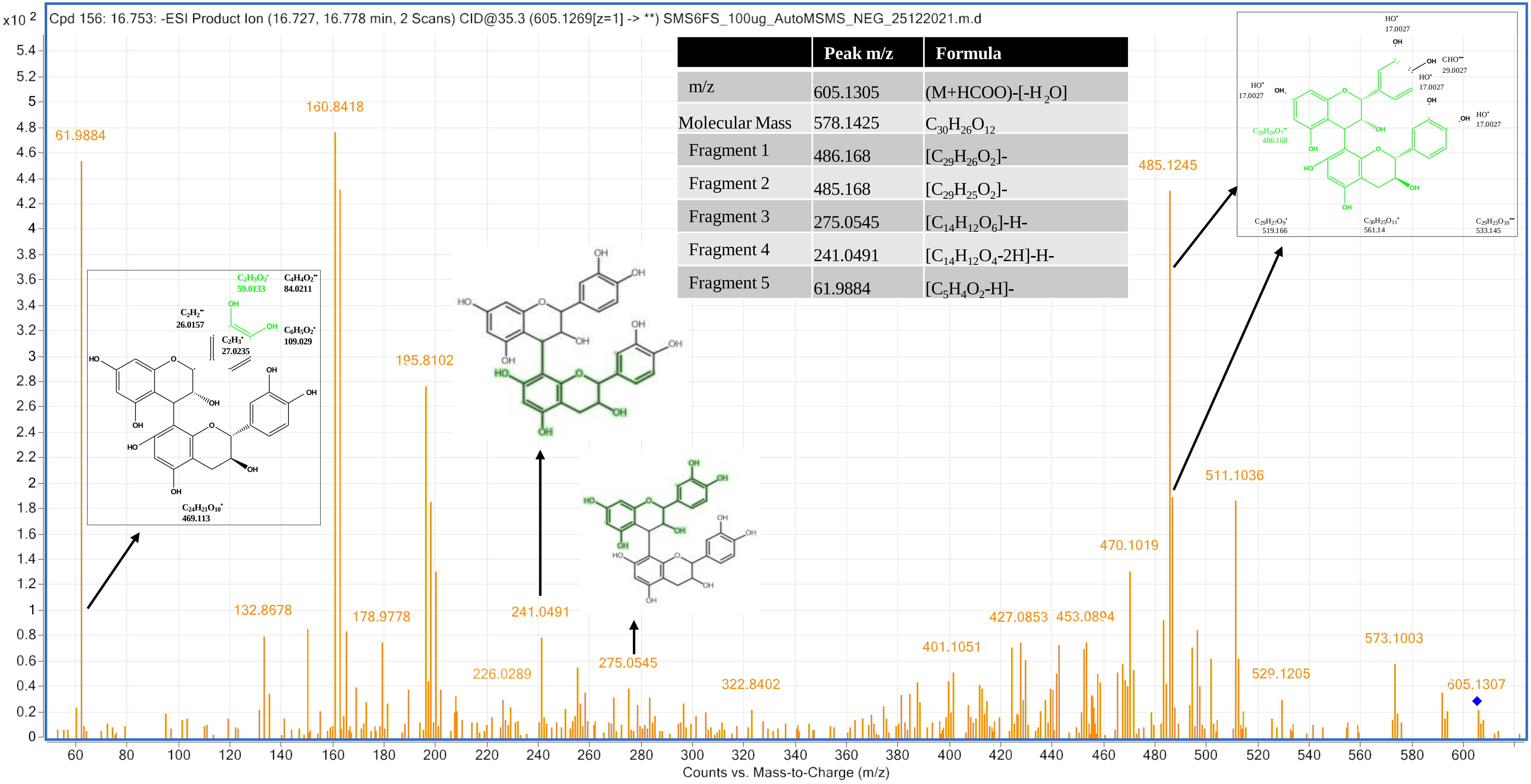

	Peak m/z	Formula
m/z	605.1305	(M+HCOO)-[-H_2O]
Molecular Mass	578.1425	$C_{30}H_{26}O_{12}$
Fragment 1	486.168	[$C_{29}H_{26}O_2$]-
Fragment 2	485.168	[$C_{29}H_{25}O_2$]-
Fragment 3	275.0545	[$C_{14}H_{12}O_6$]-H-
Fragment 4	241.0491	[$C_{14}H_{12}O_4$-2H]-H-
Fragment 5	61.9884	[$C_5H_4O_2$-H]-

Theaflavin (6UFS) _22.497

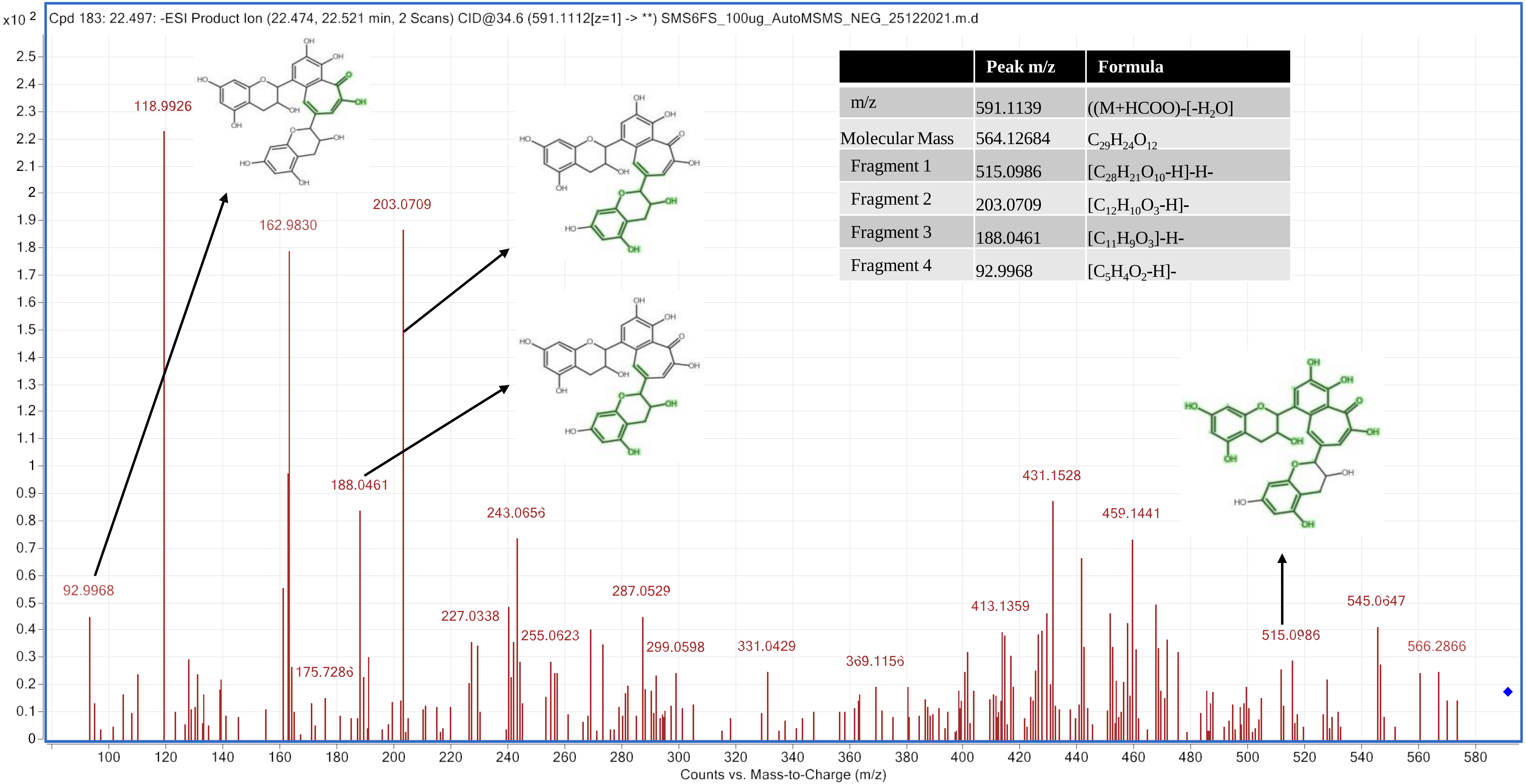

	Peak m/z	Formula
m/z	591.1139	((M+HCOO)-[-H_2O]
Molecular Mass	564.12684	$C_{29}H_{24}O_{12}$
Fragment 1	515.0986	[$C_{28}H_{21}O_{10}$-H]-H-
Fragment 2	203.0709	[$C_{12}H_{10}O_3$-H]-
Fragment 3	188.0461	[$C_{11}H_9O_3$]-H-
Fragment 4	92.9968	[$C_5H_4O_2$-H]-

In the Unique quarter-strength mode (UQS), MS/MS spectra of 7 molecules were generated

6-O-desmethylterphenyllin (1UQS) _11.712

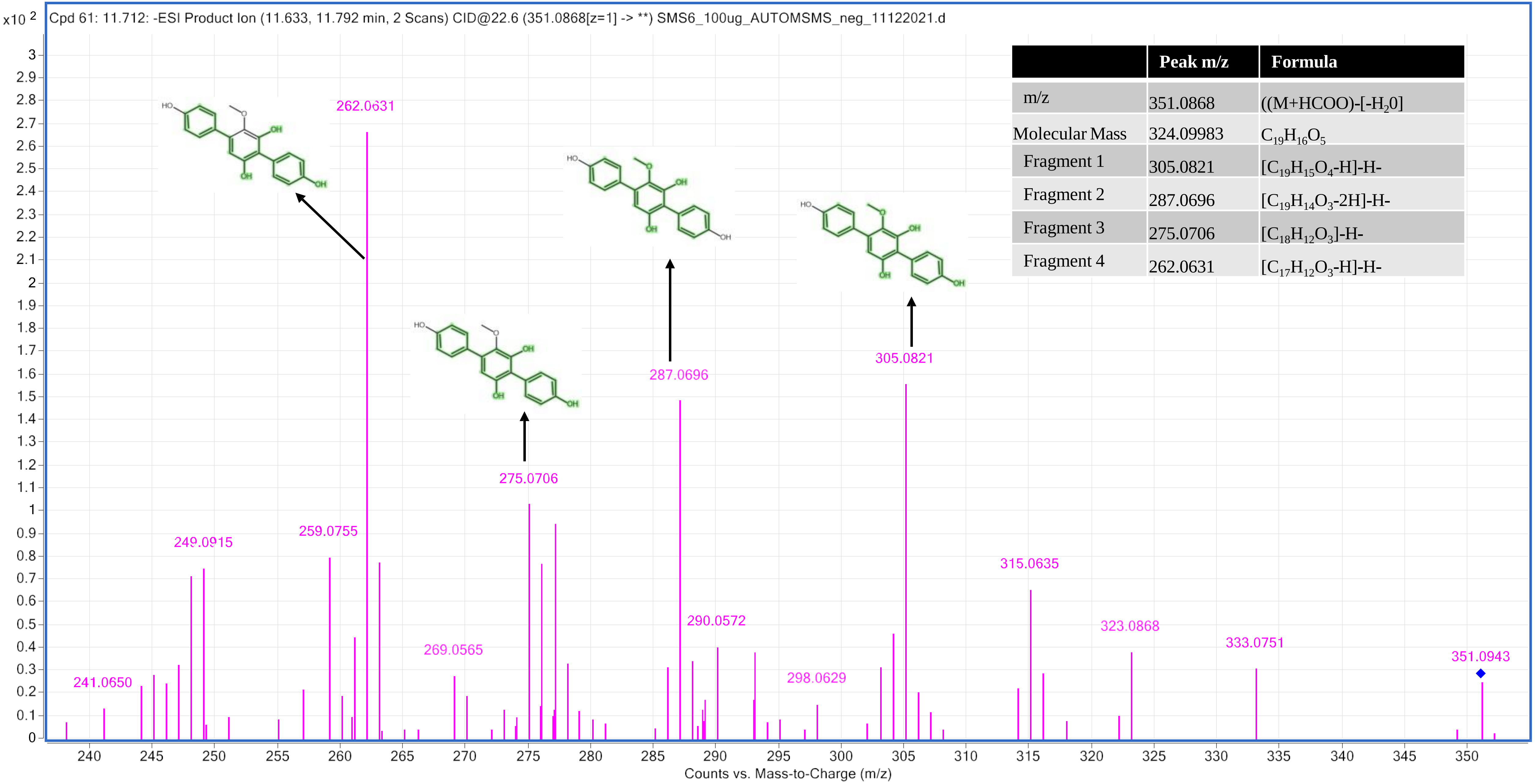

	Peak m/z	Formula
m/z	351.0868	((M+HCOO)-[-$H_2$0]
Molecular Mass	324.09983	$C_{19}H_{16}O_5$
Fragment 1	305.0821	[$C_{19}H_{15}O_4$-H]-H-
Fragment 2	287.0696	[$C_{19}H_{14}O_3$-2H]-H-
Fragment 3	275.0706	[$C_{18}H_{12}O_3$]-H-
Fragment 4	262.0631	[$C_{17}H_{12}O_3$-H]-H-

Altertoxin I (2UQS) _11.712

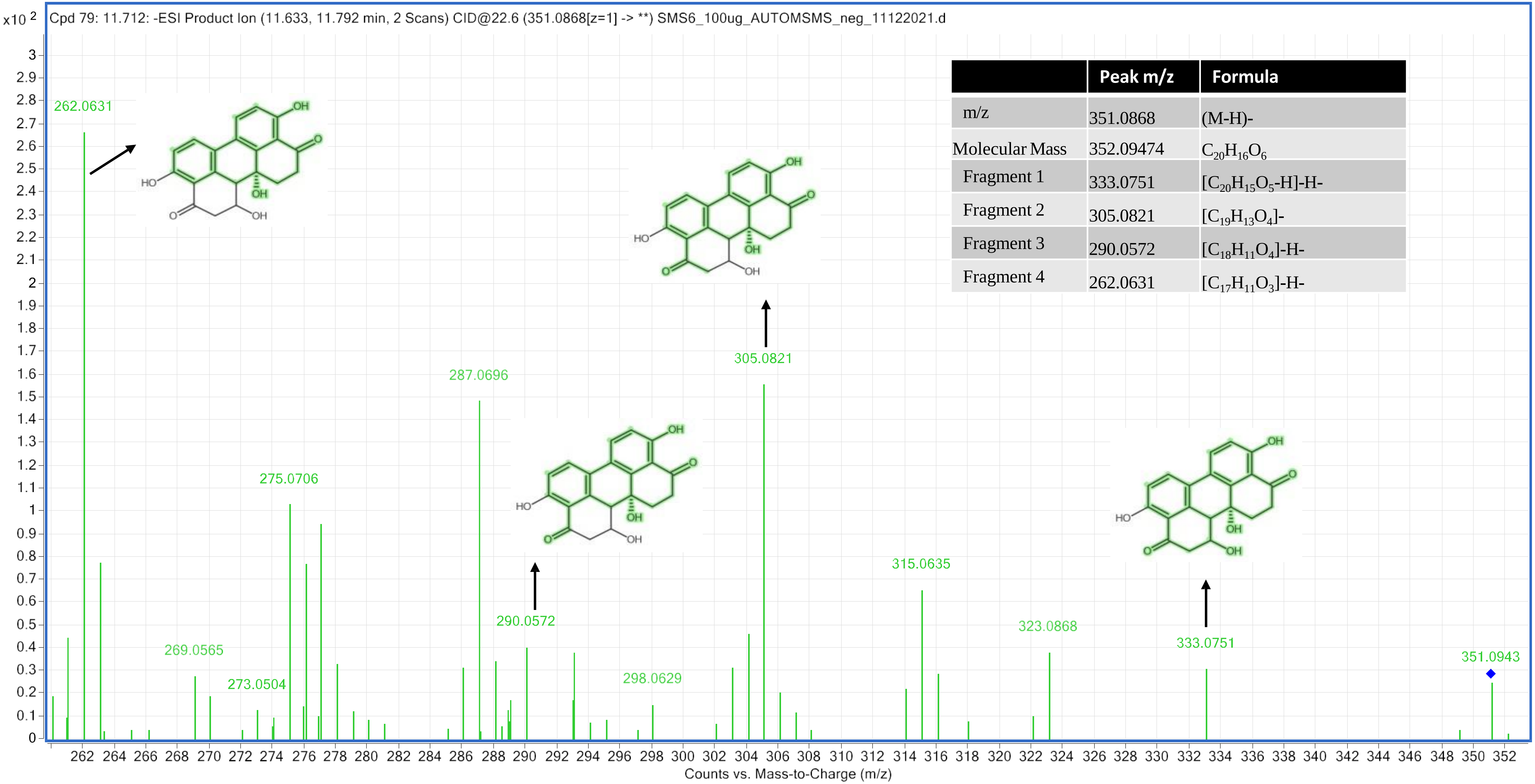

	Peak m/z	Formula
m/z	351.0868	(M-H)-
Molecular Mass	352.09474	$C_{20}H_{16}O_6$
Fragment 1	333.0751	$[C_{20}H_{15}O_5$-H]-H-
Fragment 2	305.0821	$[C_{19}H_{13}O_4]$-
Fragment 3	290.0572	$[C_{18}H_{11}O_4]$-H-
Fragment 4	262.0631	$[C_{17}H_{11}O_3]$-H-

Altechromone B (3UQS) _18.764

	Peak m/z	Formula
m/z	245.0823	(M-H)-
Molecular Mass	246.08926	$C_{14}H_{14}O_6$
Fragment 1	230.0574	$[C_{13}H_{11}O_4]$-H-
Fragment 2	188.0488	$[C_{11}H_9O_3]$-H-
Fragment 3	160.0532	$[C_{14}H_{14}O_4]$
Fragment 4	146.0376	$[C_9H_7O_2]$-H-

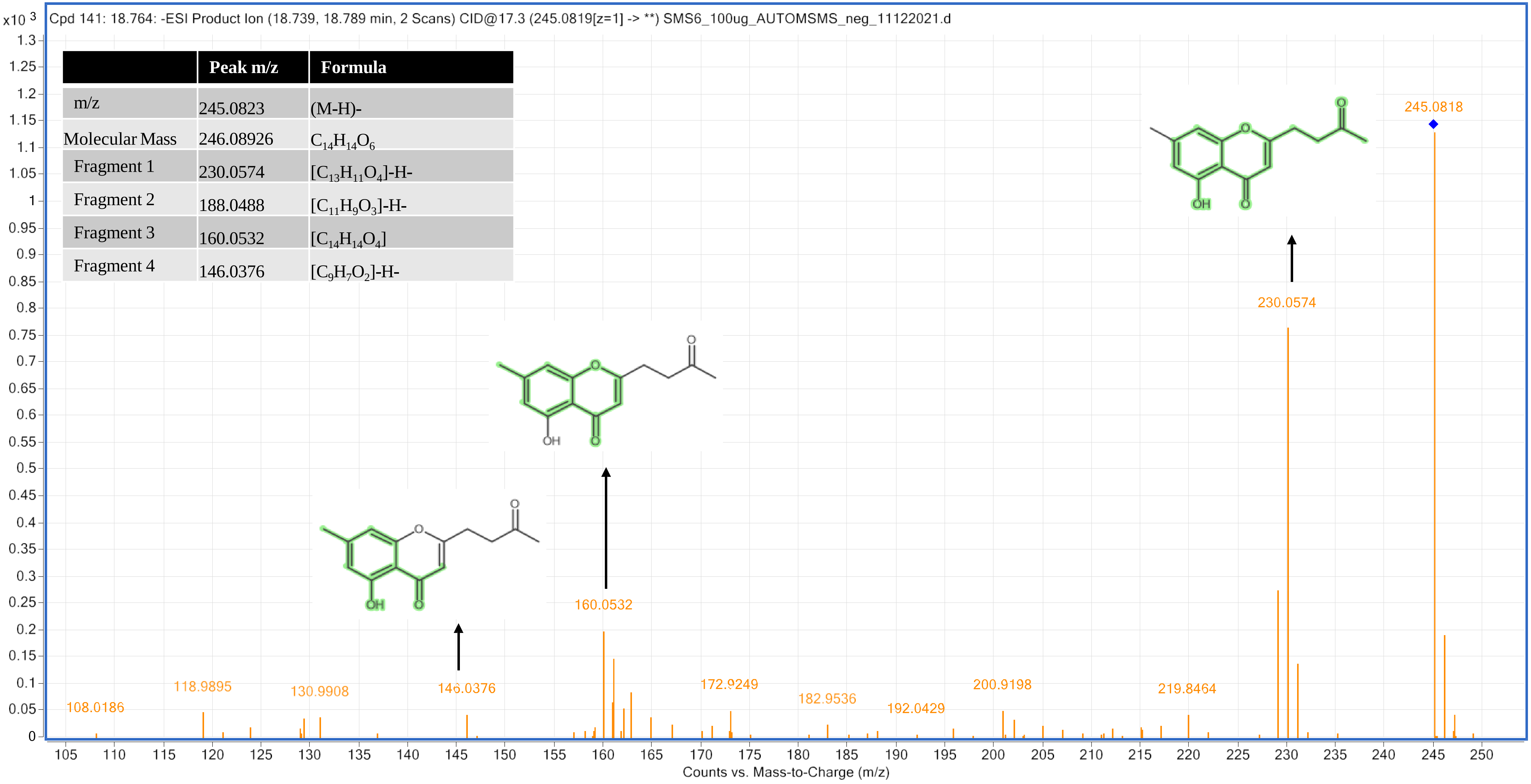

Botryorhodine F (4UQS) _20.017

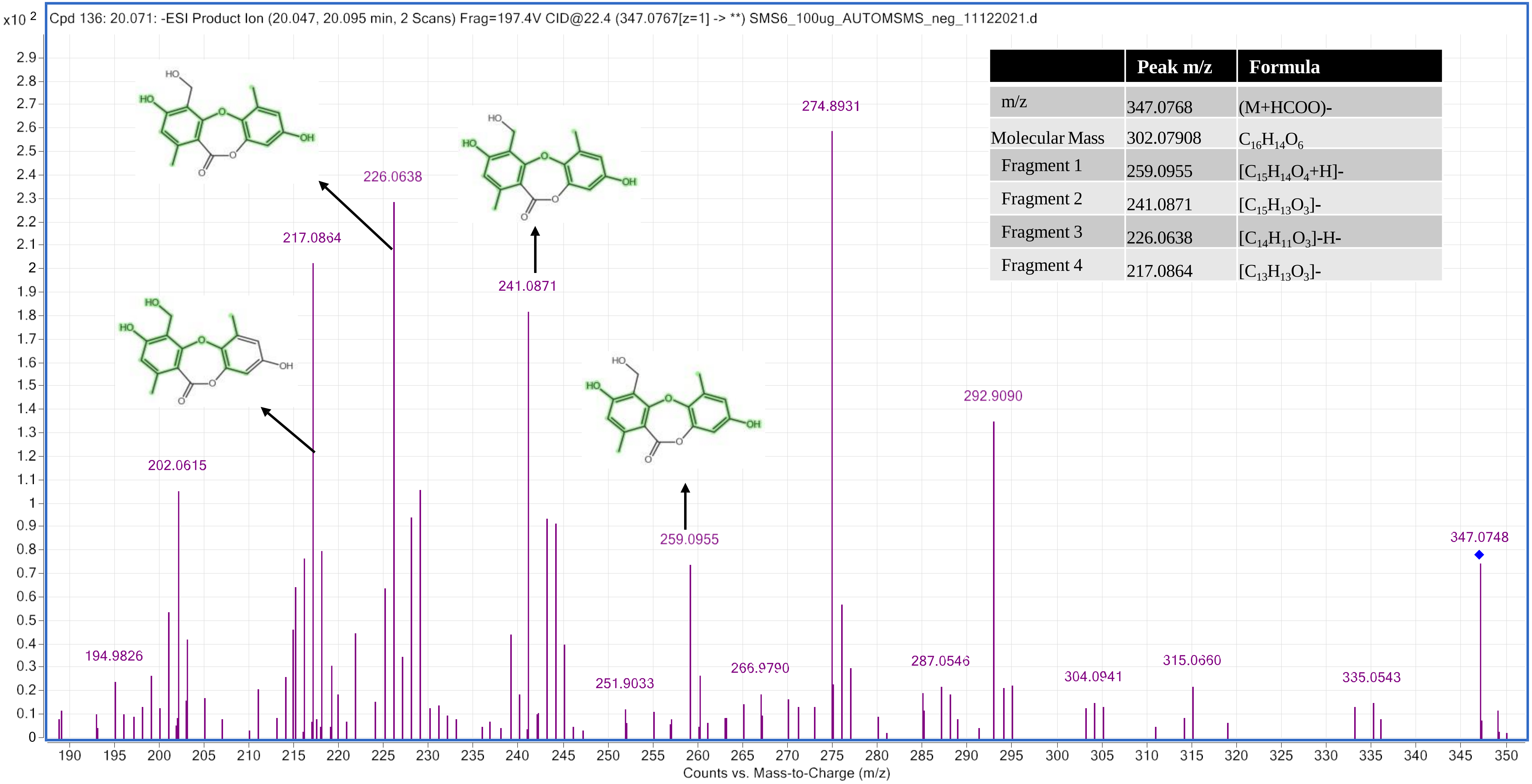

	Peak m/z	Formula
m/z	347.0768	(M+HCOO)-
Molecular Mass	302.07908	$C_{16}H_{14}O_6$
Fragment 1	259.0955	$[C_{15}H_{14}O_4+H]^-$
Fragment 2	241.0871	$[C_{15}H_{13}O_3]^-$
Fragment 3	226.0638	$[C_{14}H_{11}O_3]$-H-
Fragment 4	217.0864	$[C_{13}H_{13}O_3]^-$

3',4',7-Trihydroxyisoflavanone (5UQS) _22.848

	Peak m/z	Formula
m/z	317.0666	(M+HCOO)-
Molecular Mass	272.06851	$C_{15}H_{12}O_5$
Fragment 1	271.053	$[C_{15}H_{11}O_5]^-$
Fragment 2	270.0169	$[C_{15}H_{10}O_5]^-$
Fragment 3	242.0206	$[C_{14}H_{10}O_4]^-$
Fragment 4	226.0275	$[C_{14}H_{10}O_3]^-$
Fragment 5	198.0313	$[C_{12}H_8O_3\text{-}H]^-$
Fragment 6	214.0267	$[C_{12}H_7O_4\text{-}H]^-$
Fragment 7	256.0358	$[C_{14}H_{10}O_5\text{-}H]\text{-}H^-$

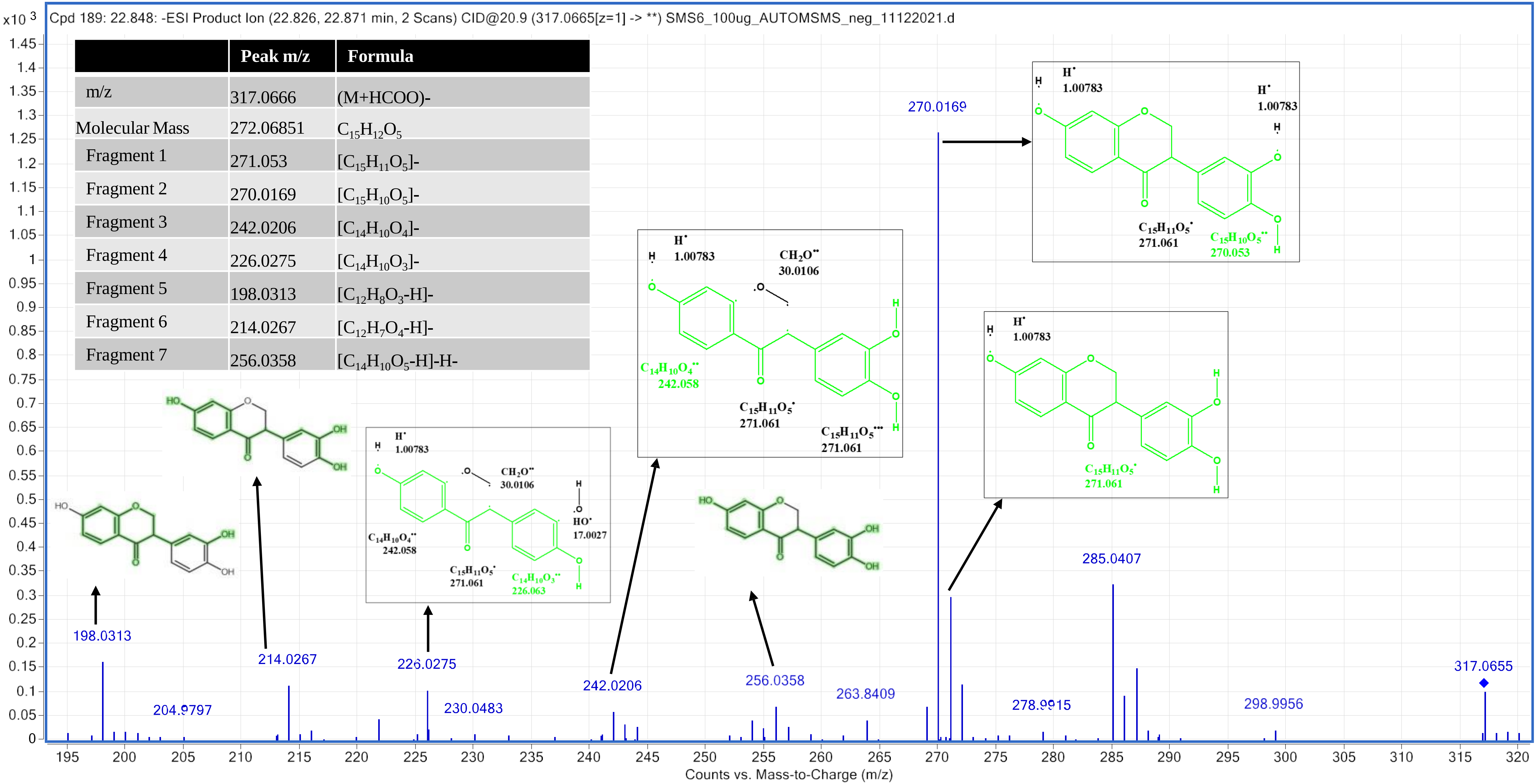

Alternariol 9-methyl ether (6UQS) _25.377

	Peak m/z	Formula
m/z	317.0669	(M+HCOO)-
Molecular Mass	272.06851	$C_{15}H_{12}O_5$
Fragment 1	271.061	$[C_{15}H_{11}O_5]^-$
Fragment 2	270.061	$[C_{15}H_{10}O_5]^-$
Fragment 3	198.0338	$[C_{13}H_{10}O_2]^-$
Fragment 4	242.0206	$[C_{13}H_6O_5]^-$

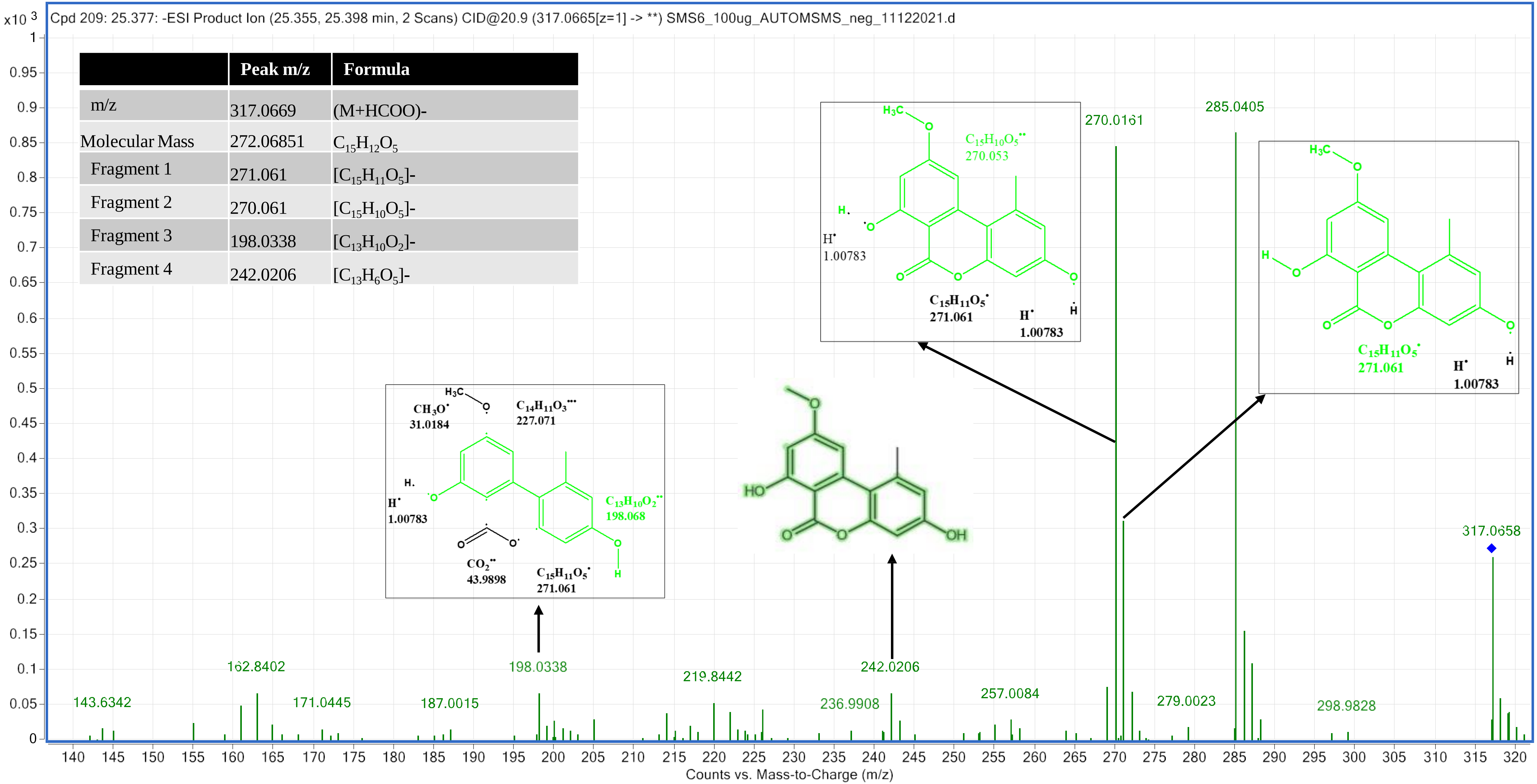

Morin (7UQS) 26.284

	Peak m/z	Formula
m/z	301.0349	(M-H)-
Molecular Mass	302.04267	$C_{15}H_{10}O_7$
Fragment 1	301.0349	$[C_{14}H_9O_7]$-
Fragment 2	273.0399	$[C_{14}H_9O_5]$-
Fragment 3	258.0159	$[C_{13}H_8O_6$-H]-H-
Fragment 4	230.0240	$[C_{12}H_7O_5]$-H-

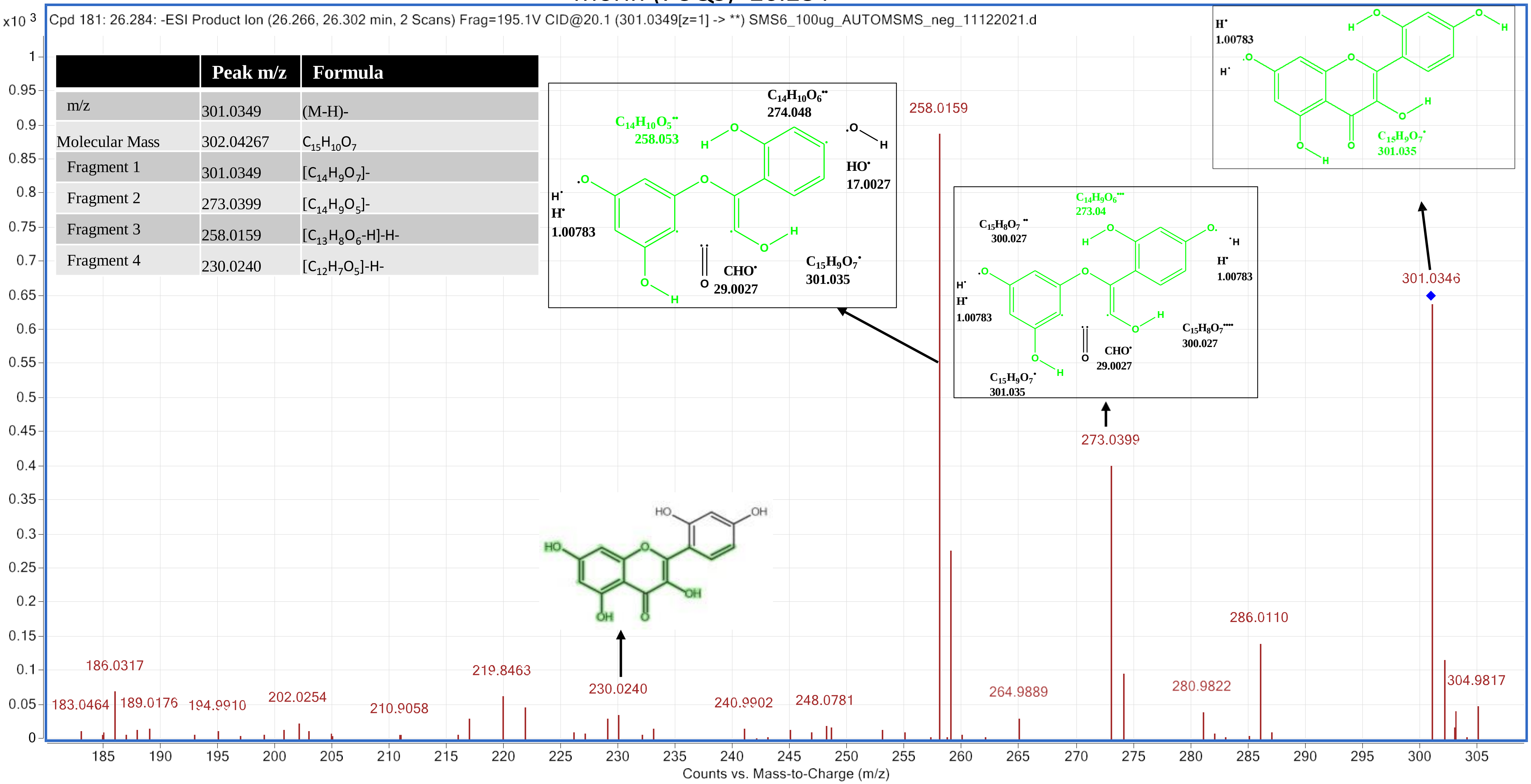

- In the Common quarter-strength mode (CQS), MS/MS spectra of 13 molecules were generated

4-Hydroxymellein(1CQS) _18.157

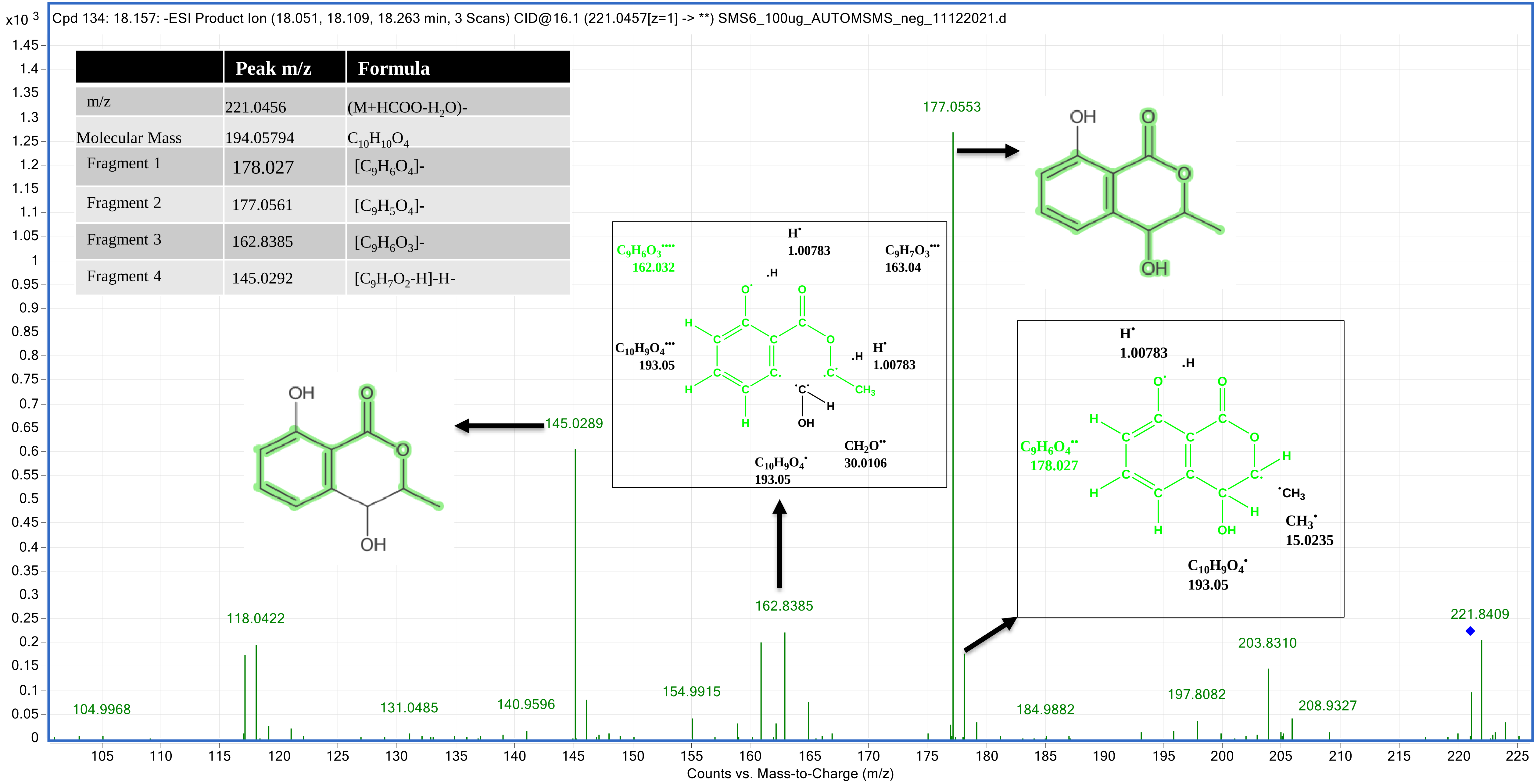

	Peak m/z	Formula
m/z	221.0456	$(M+HCOO-H_2O)-$
Molecular Mass	194.05794	$C_{10}H_{10}O_4$
Fragment 1	178.027	$[C_9H_6O_4]-$
Fragment 2	177.0561	$[C_9H_5O_4]-$
Fragment 3	162.8385	$[C_9H_6O_3]-$
Fragment 4	145.0292	$[C_9H_7O_2-H]-H-$

5'-Epialtenuene (2CQS) _16.419

	Peak m/z	Formula
m/z	291.0878	(M-H)-
Molecular Mass	292.09474	$C_{15}H_{16}O_6$
Fragment 1	291.0866	$[C_{15}H_{15}O_6]^-$
Fragment 2	274.8918	$[C_{15}H_{14}O_5]^-$
Fragment 3	230.0569	$[C_{14}H_{14}O_3]^-$
Fragment 4	203.0343	$[C_{12}H_{11}O_3]^-$

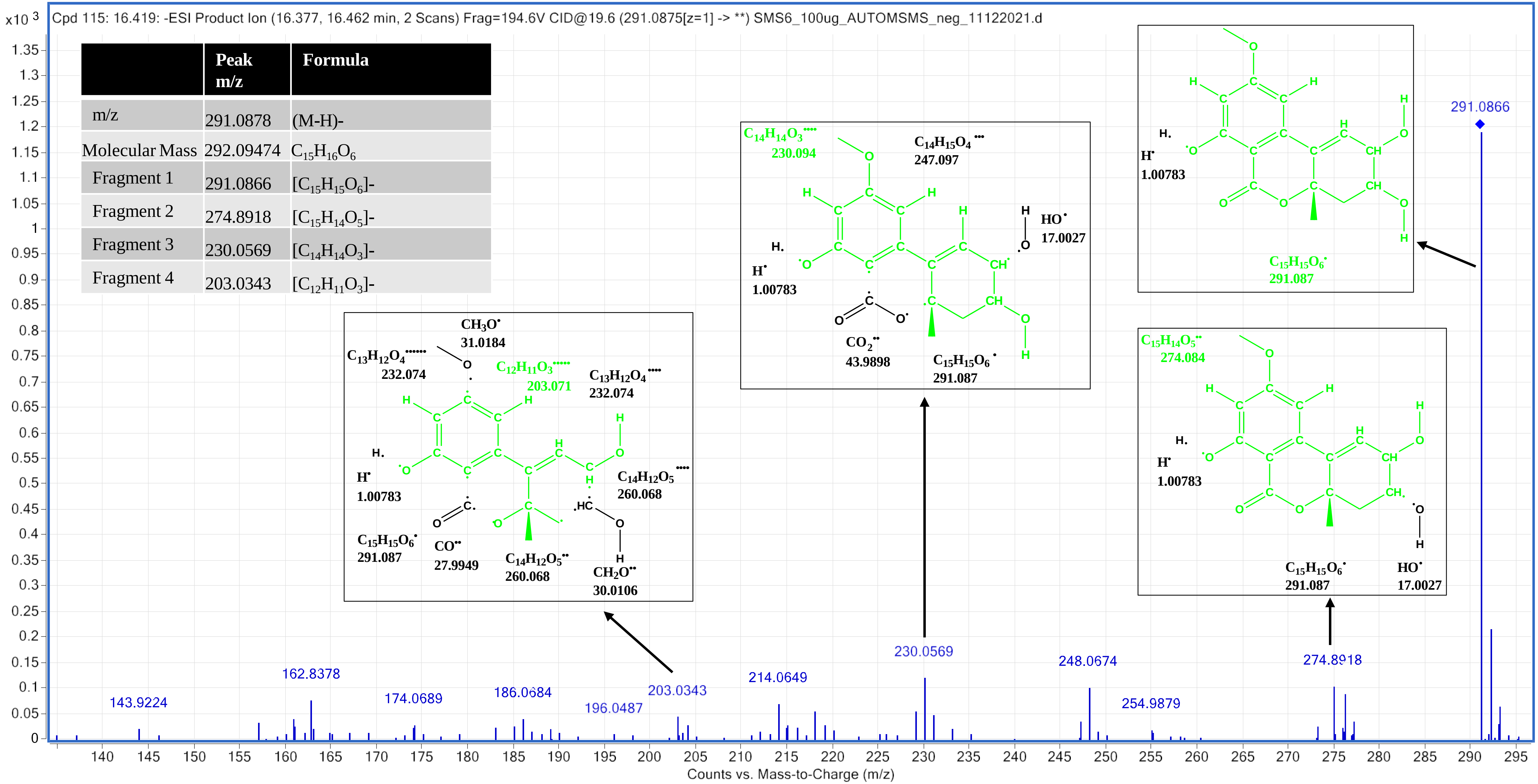

Alternarienonic acid (3CQS) _14.99

	Peak m/z	Formula
m/z	277.07908	(M-H)-
Molecular Mass	278.0792	$C_{14}H_{14}O_6$
Fragment 1	277.0716	$[C_{14}H_{12}O_6]^-$
Fragment 2	233.0813	$[C_{13}H_{13}O_4]^-$
Fragment 3	218.0584	$[C_{12}H_{10}O_4]^-$
Fragment 4	205.0860	$[C_{12}H_{13}O_3]^-$
Fragment 5	190.0639	$[C_{11}H_9O_3]^-$

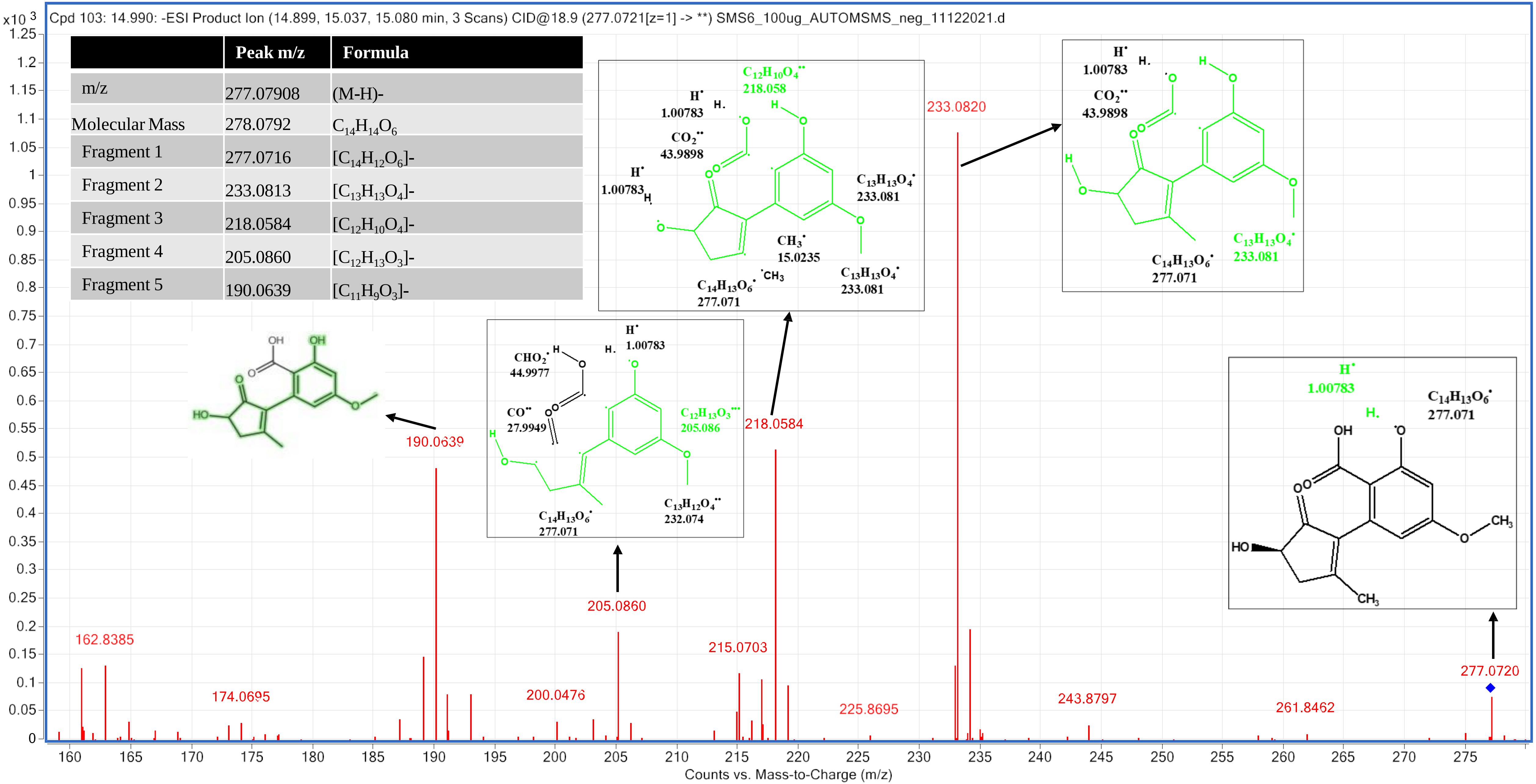

(+)-talaroflavone (4CQS) _21.427

	Peak m/z	Formula
m/z	257.0465	(M-H)- [-H_2O]
Molecular Mass	276.06342	$C_{14}H_{12}O_6$
Fragment 1	257.0457	[$C_{14}H_{10}O_5$]-
Fragment 2	228.9590	[$C_{13}H_8O_4$]-
Fragment 3	215.0355	[$C_{12}H_7O_4$]-
Fragment 4	202.063	[$C_{12}H_{10}O_3$]-

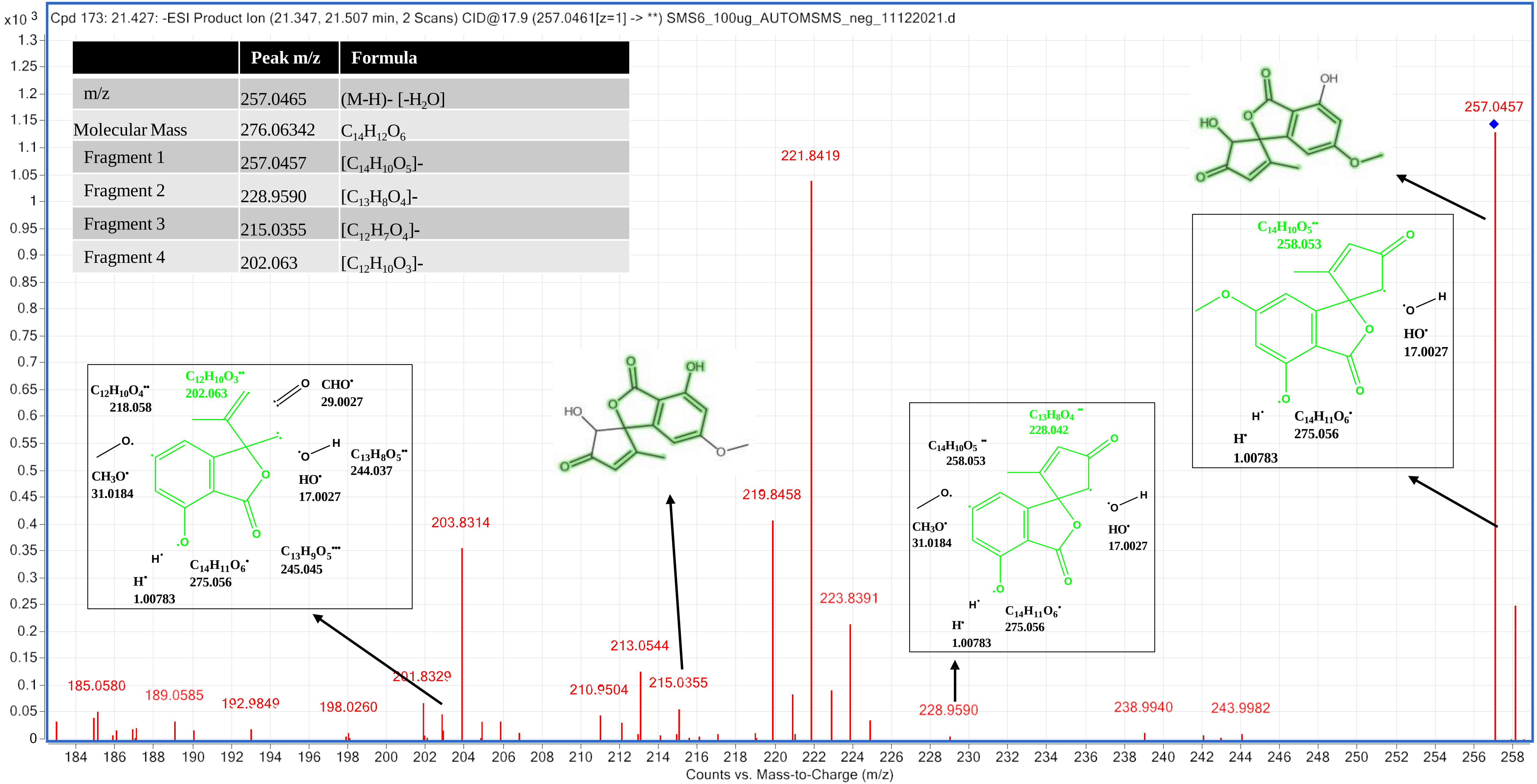

2,5-dimethyl-7-hydroxychromone (5CQS) _14.807

	Peak m/z	Formula
m/z	189.0563	(M-H)-
Molecular Mass	190.06303	$C_{11}H_{10}O_3$
Fragment 1	189.055	$[C_{11}H_9O_3]$-
Fragment 2	174.0355	$[C_{10}H_6O_3]$-
Fragment 3	162.8384	$[C_{10}H_{10}O_2]$-
Fragment 4	105.0328	$[C_{11}H_7O]$-
Fragment 5	146.0358	$[C_9H_7O_2]$-H-

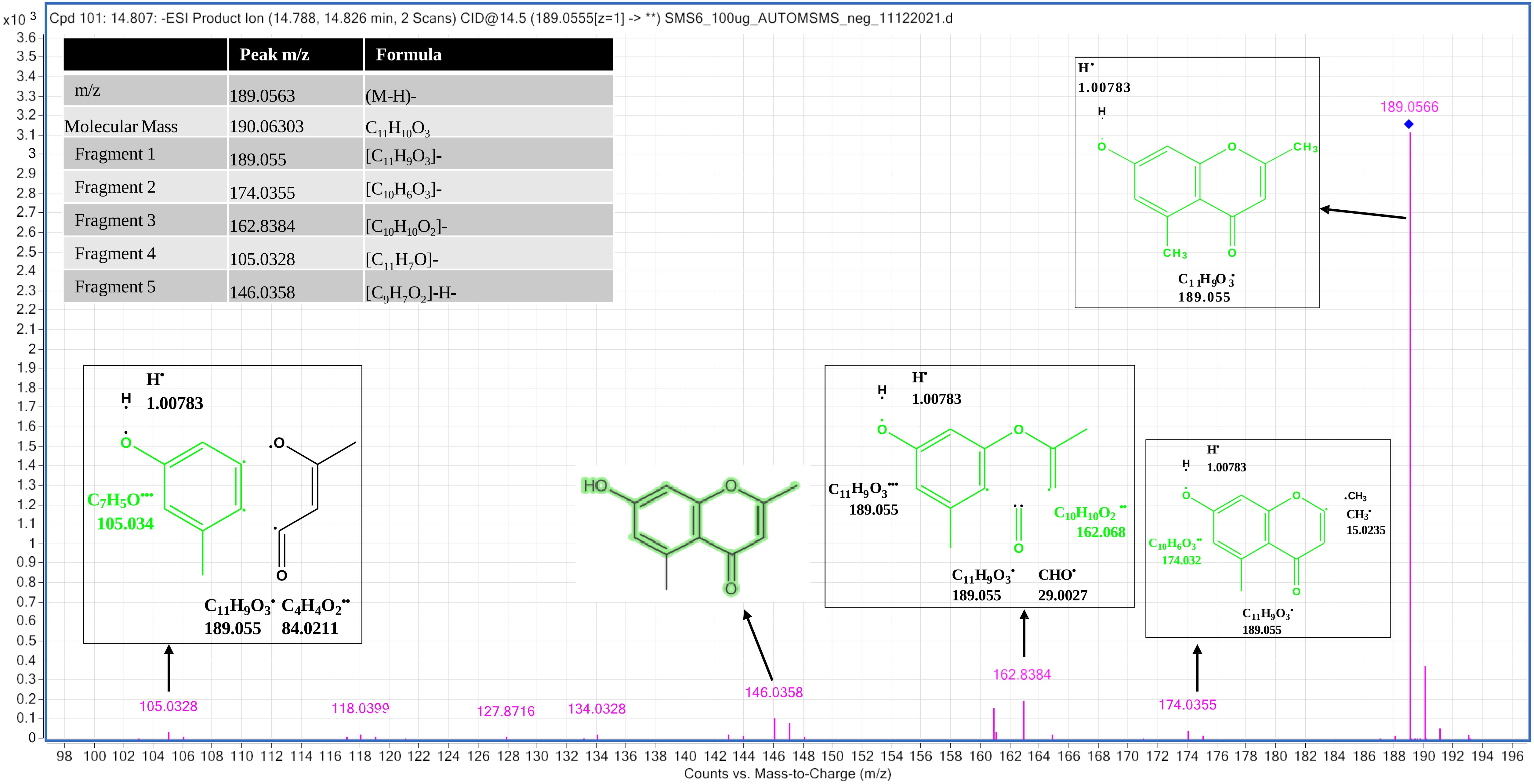

Alternarian acid (6CQS) _15.882

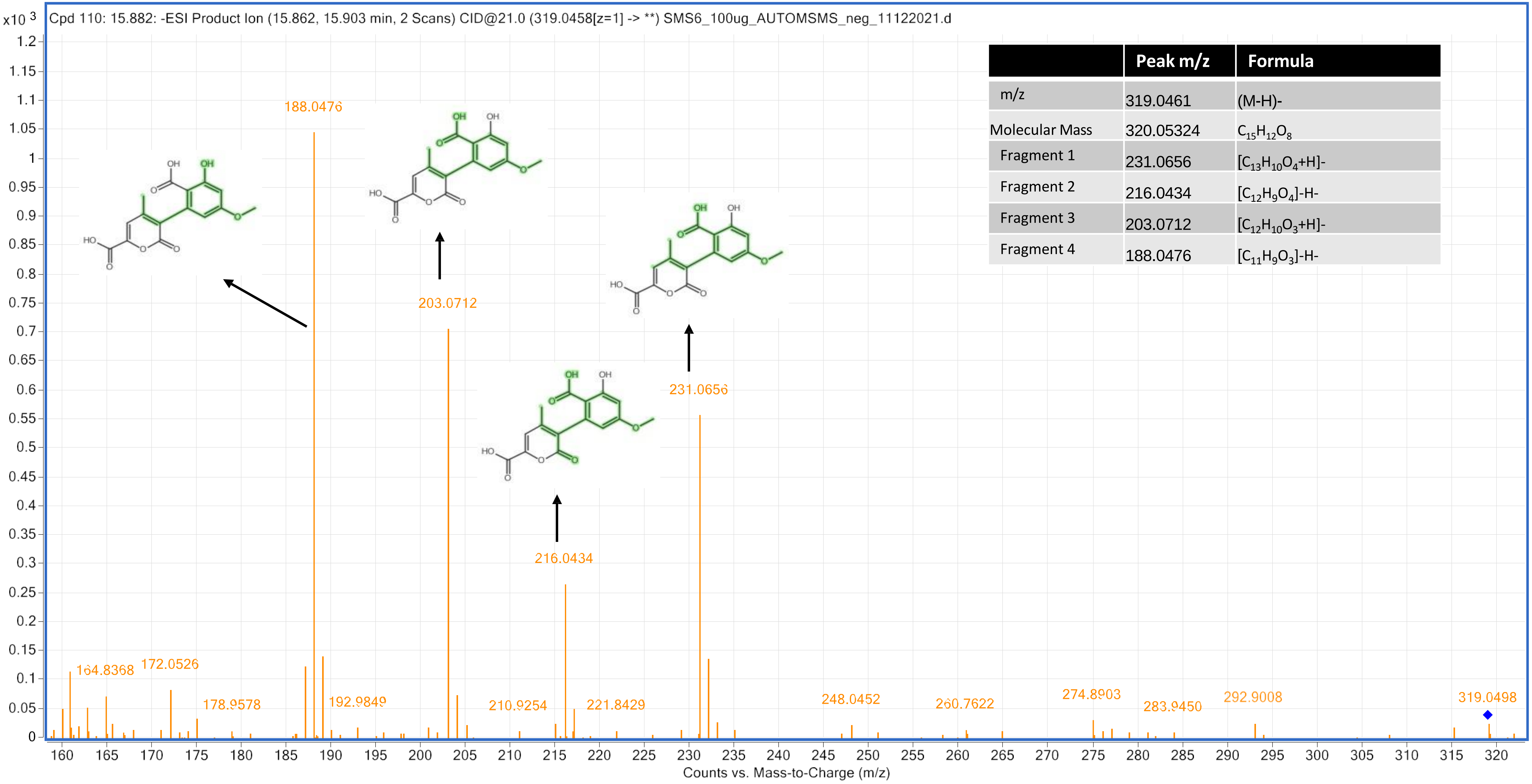

	Peak m/z	Formula
m/z	319.0461	(M-H)-
Molecular Mass	320.05324	$C_{15}H_{12}O_8$
Fragment 1	231.0656	$[C_{13}H_{10}O_4+H]^-$
Fragment 2	216.0434	$[C_{12}H_9O_4]$-H-
Fragment 3	203.0712	$[C_{12}H_{10}O_3+H]^-$
Fragment 4	188.0476	$[C_{11}H_9O_3]$-H-

1,8-dihydroxynaphthalene (7CQS) _10.889

	Peak m/z	Formula
m/z	205.0509	(M+HCOO)-
Molecular Mass	160.05246	$C_{10}H_8O_2$
Fragment 1	143.0502	$[C_{10}H_7O]^-$
Fragment 2	118.9939	$[C_8H_6O]^-$
Fragment 3	105.0351	$[C_7H_5O]^-$

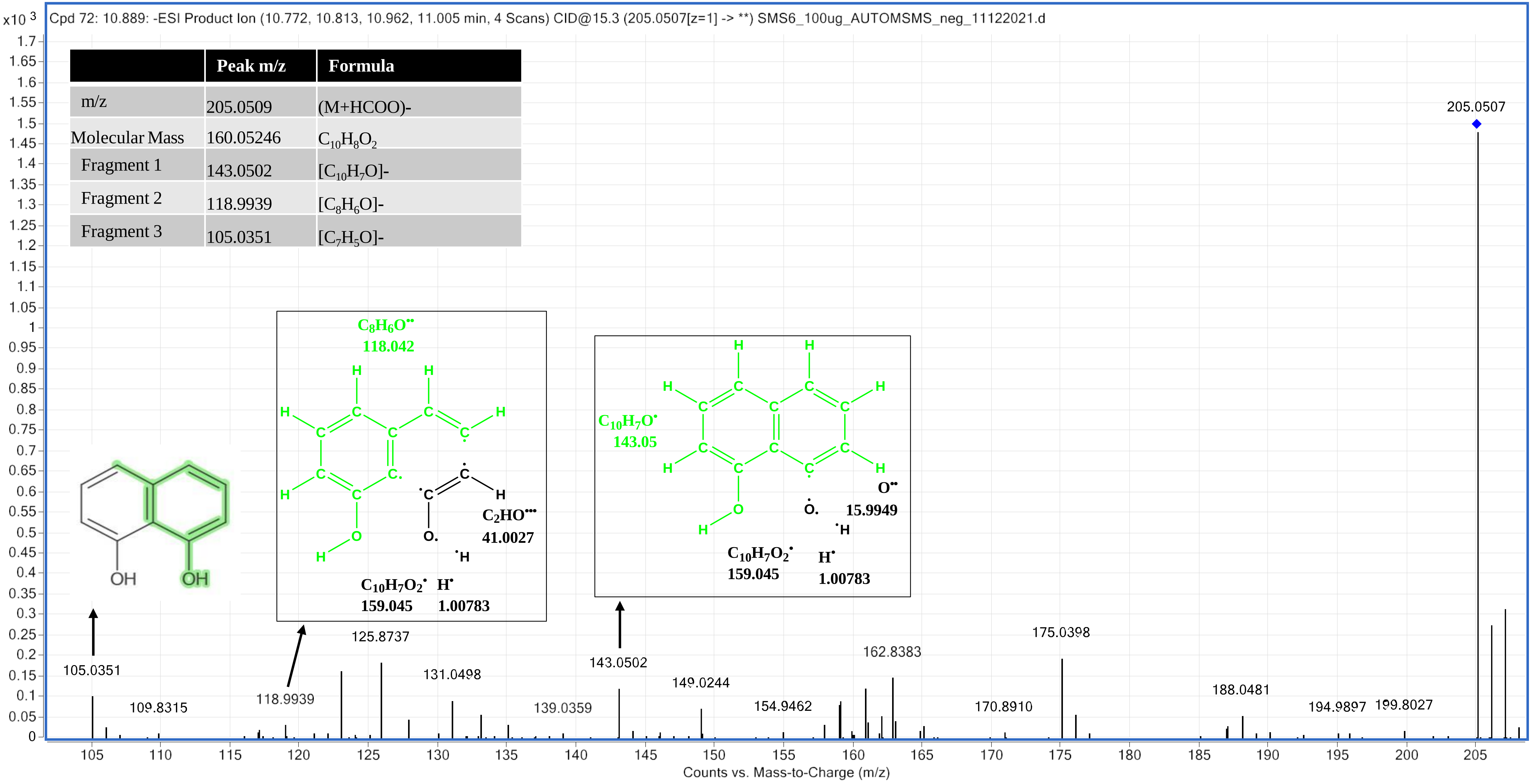

Aspergone Q (8CQS) _17.024

	Peak m/z	Formula
m/z	223.0611	(M-H)- [-H_2O]
Molecular Mass	242.07908	$C_{11}H_{14}O_6$
Fragment 1	191.0352	[$C_{10}H_{10}O_4$-2H]-H-
Fragment 2	149.0247	[$C_8H_7O_3$-H]-H-
Fragment 3	123.0462	[$C_7H_9O_2$-H]-H-

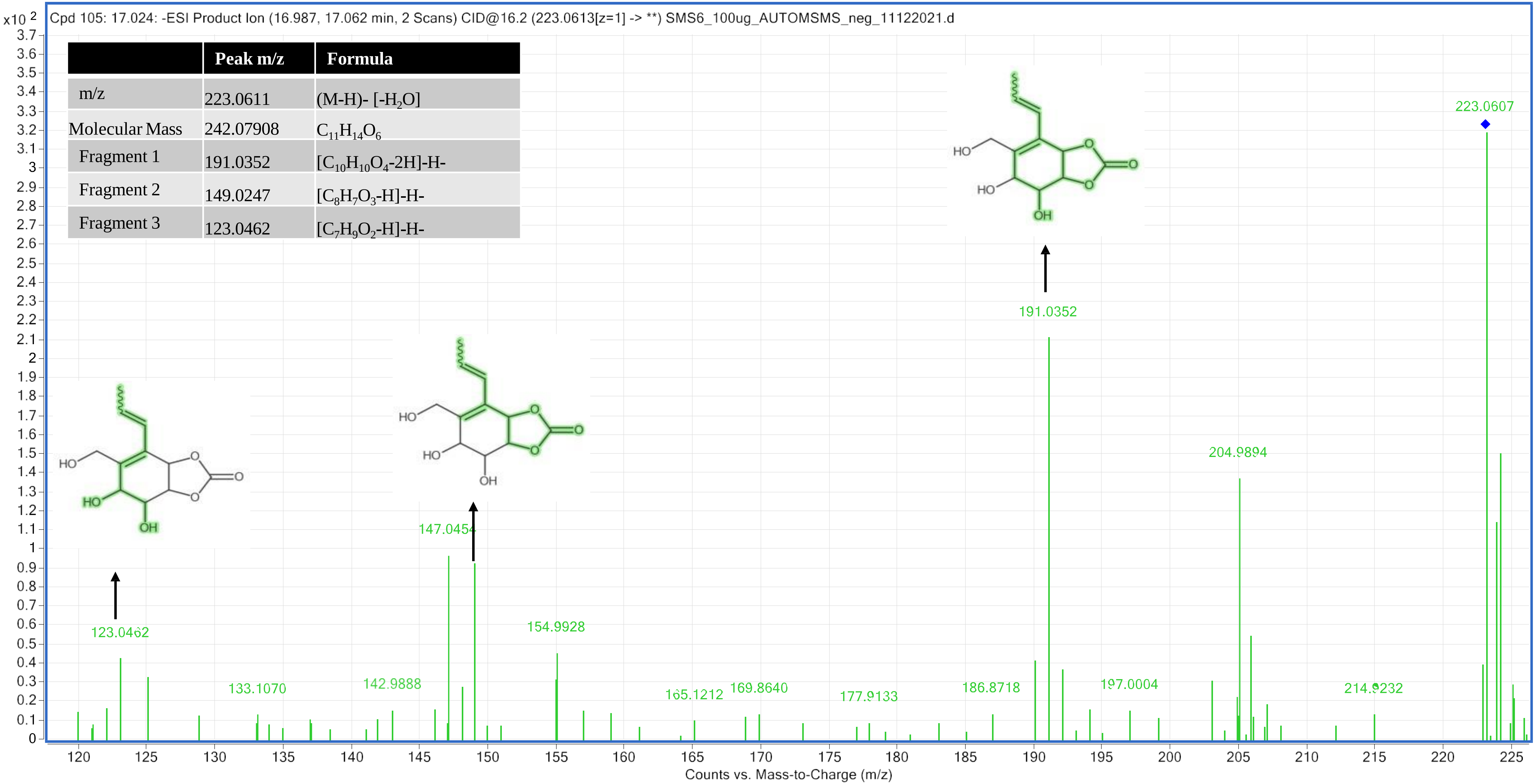

6-Epi-stemphytriol (9CQS) _18.361

	Peak m/z	Formula
m/z	349.0716	(M-H)- [-H_2O]
Molecular Mass	368.08965	$C_{20}H_{16}O_7$
Fragment 1	331.0592	[$C_{20}H_{14}O_5$-2H]-H-
Fragment 2	292.061	[$C_{14}H_{12}O_7$]-
Fragment 3	243.0665	[$C_{14}H_{11}O_4$]-

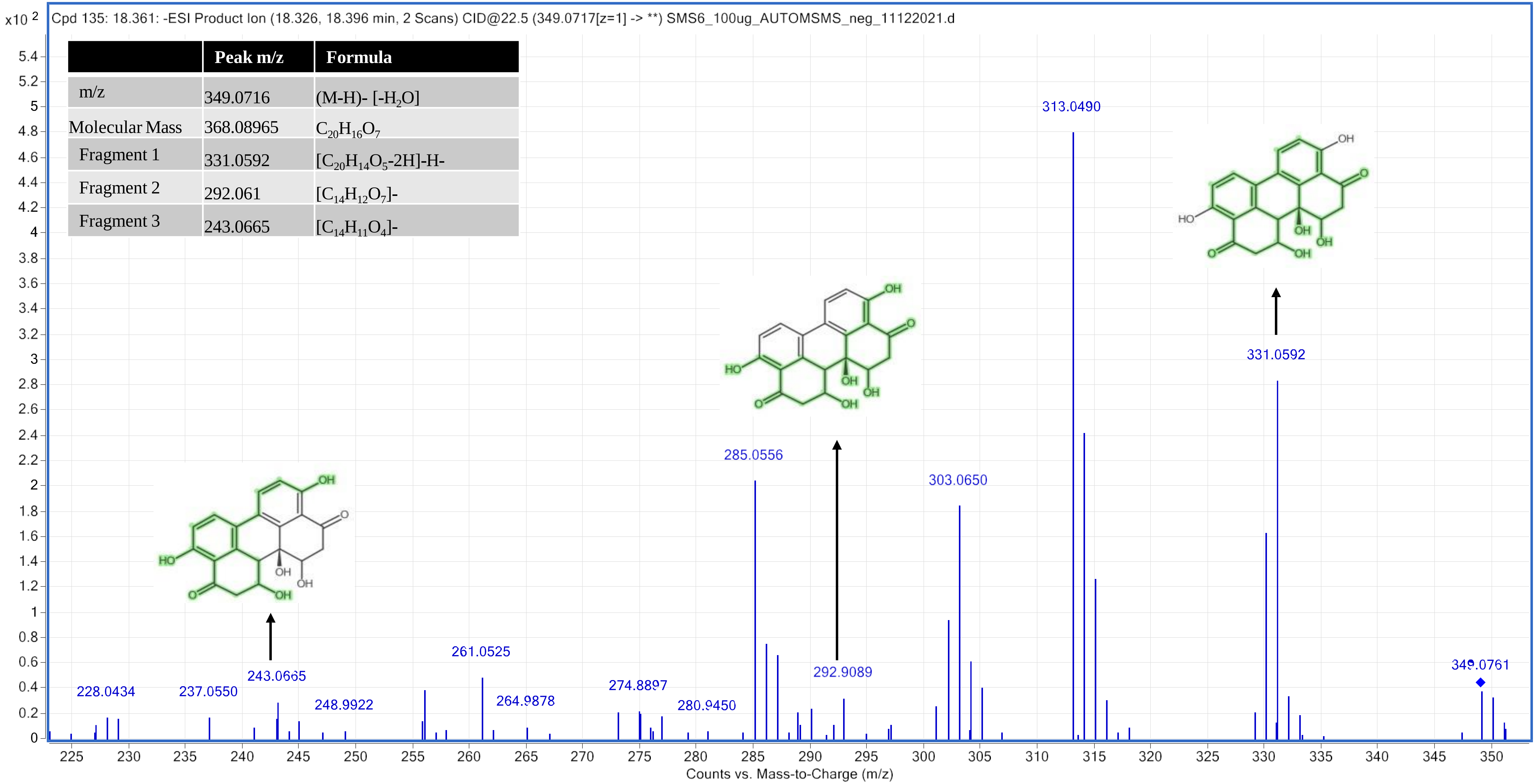

12-Methoxycitromycin (10CQS) _18.456

	Peak m/z	Formula
m/z	287.0568	(M+HCOO)-
Molecular Mass	260.06851	$C_{14}H_{12}O_5$
Fragment 1	229.05	$[C_{13}H_9O_4]^-$
Fragment 2	228.0424	$[C_{13}H_8O_4]^-$
Fragment 3	200.0467	$[C_{12}H_8O_3]^-$
Fragment 4	211.0386	$[C_{13}H_8O_3]$-H-

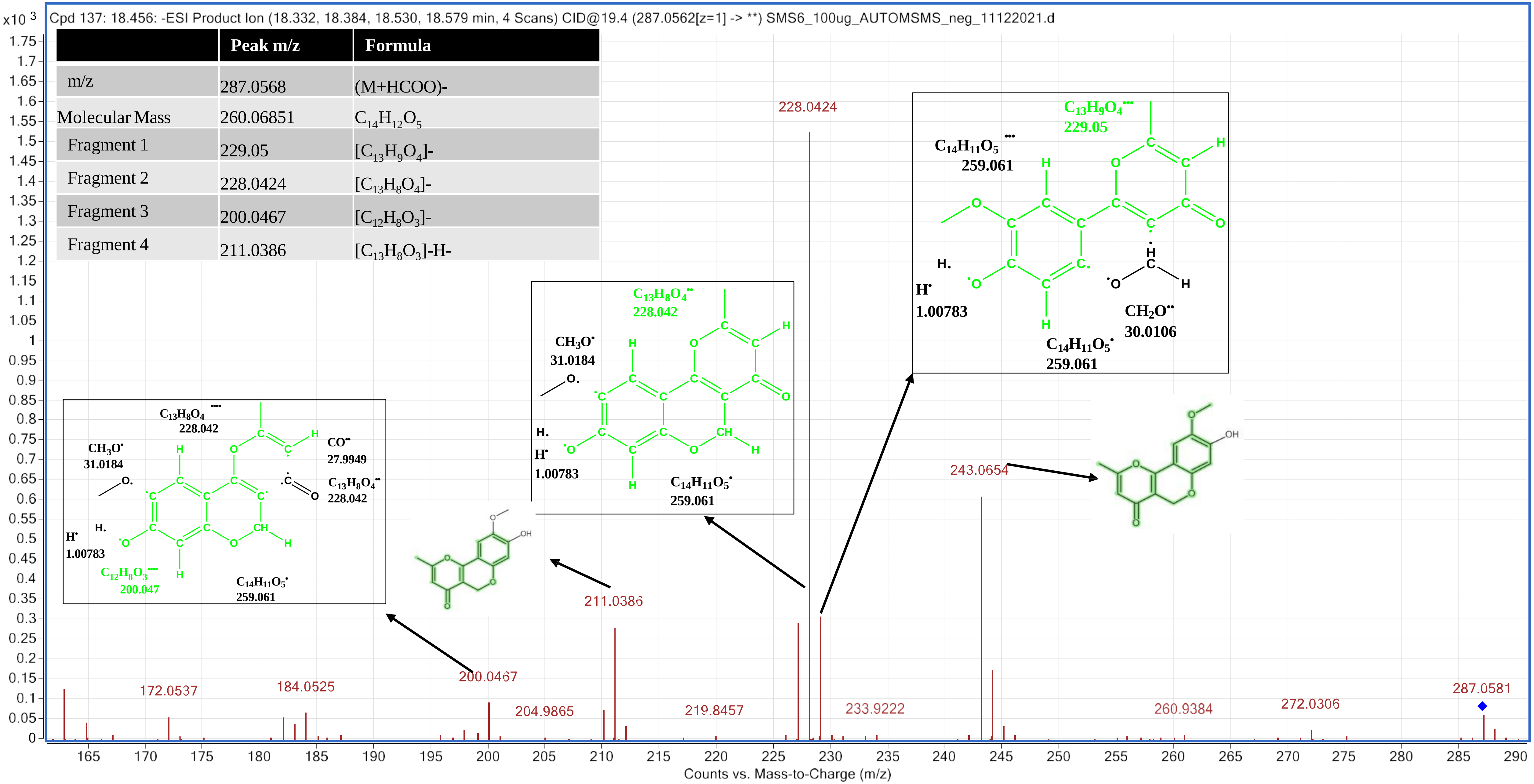

4-Hydroxyalternariol 9-methyl ether (11CQS) _23.997

	Peak m/z	Formula
m/z	287.0571	(M-H)-
Molecular Mass	288.06342	$C_{15}H_{12}O_6$
Fragment 1	287.0571	$[C_{15}H_{11}O_6]^-$
Fragment 2	272.0331	$[C_{14}H_8O_6]^-$
Fragment 3	257.0181	$[C_{13}H_5O_6]^-$
Fragment 4	228.0422	$[C_{14}H_{12}O_3]^-$
Fragment 5	188.047	$[C_{11}H_9O_3]$-H-

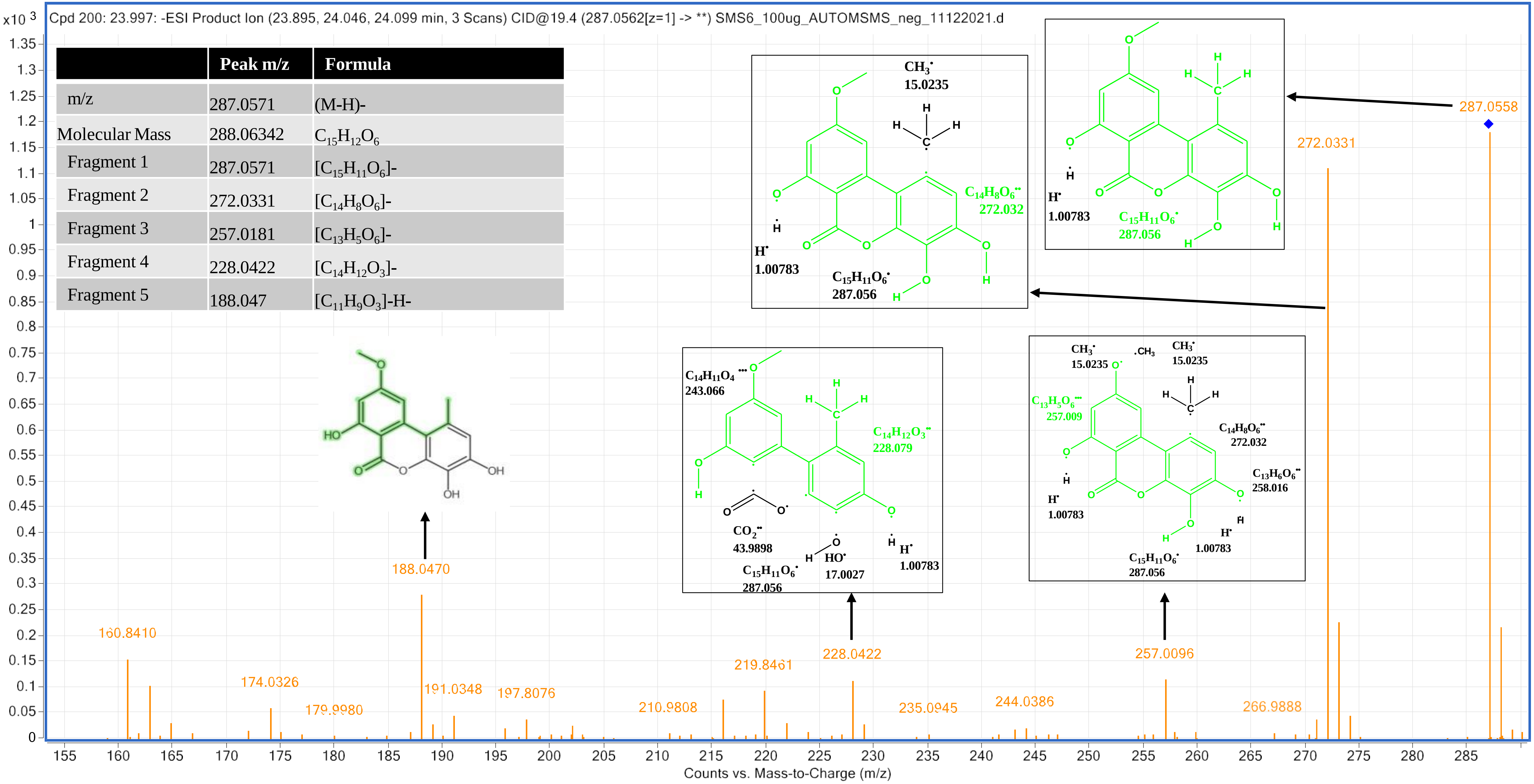

Orthosporin (12CQS) _20.349

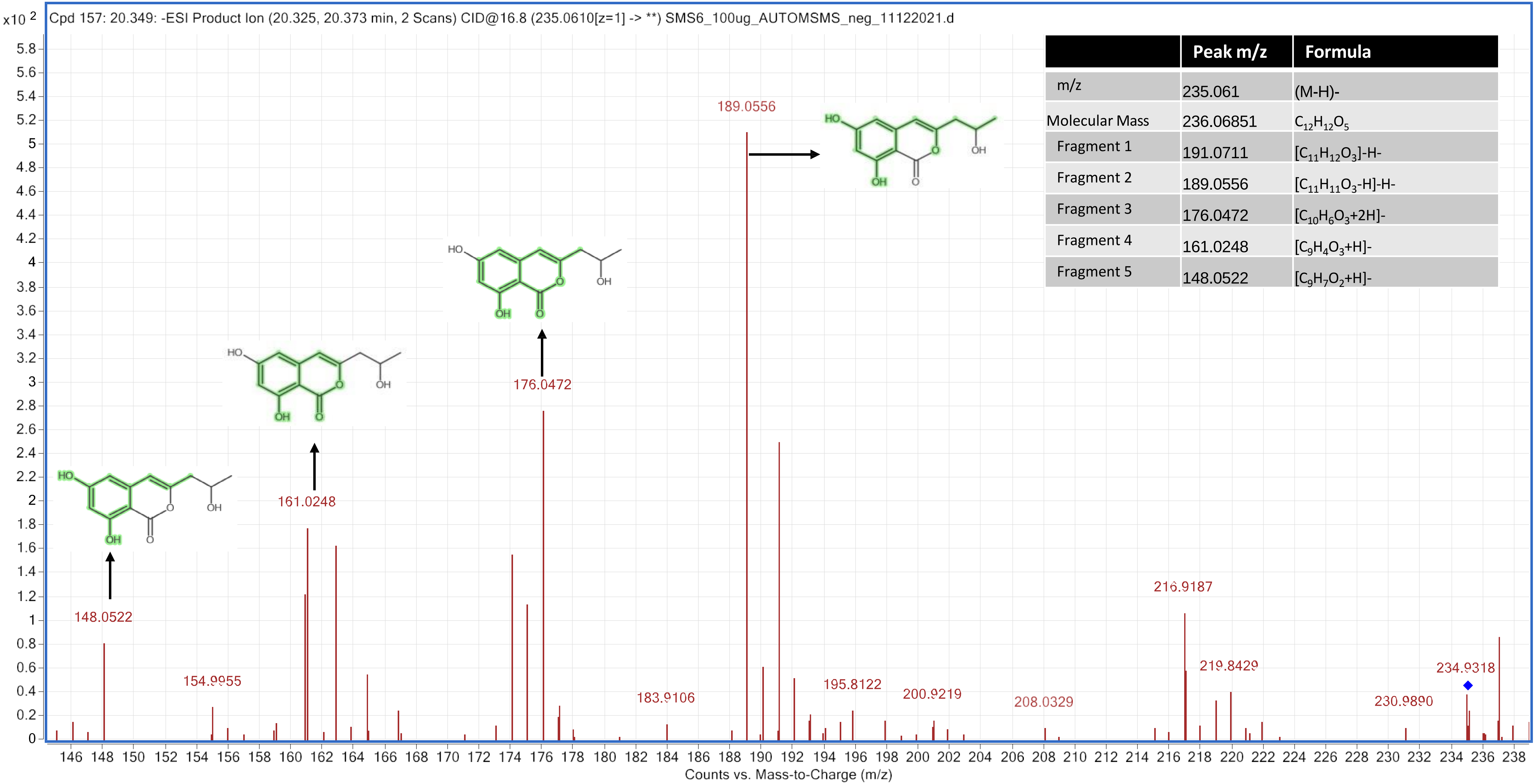

	Peak m/z	Formula
m/z	235.061	(M-H)-
Molecular Mass	236.06851	$C_{12}H_{12}O_5$
Fragment 1	191.0711	$[C_{11}H_{12}O_3]$-H-
Fragment 2	189.0556	$[C_{11}H_{11}O_3$-H]-H-
Fragment 3	176.0472	$[C_{10}H_6O_3$+2H]-
Fragment 4	161.0248	$[C_9H_4O_3$+H]-
Fragment 5	148.0522	$[C_9H_7O_2$+H]-

Altenusin (13CQS) _22.848

	Peak m/z	Formula
m/z	317.0666	(M+HCOO)- [-H_2O]
Molecular Mass	290.07908	$C_{15}H_{14}O_6$
Fragment 1	271.019	$[C_{15}H_{11}O_5]^-$
Fragment 2	270.169	$[C_{15}H_{10}O_5]^-$
Fragment 3	242.0206	$[C_{14}H_{10}O_4]^-$
Fragment 4	214.0267	$[C_{13}H_{10}O_3]^-$
Fragment 5	198.0313	$[C_{13}H_{10}O_2]^-$

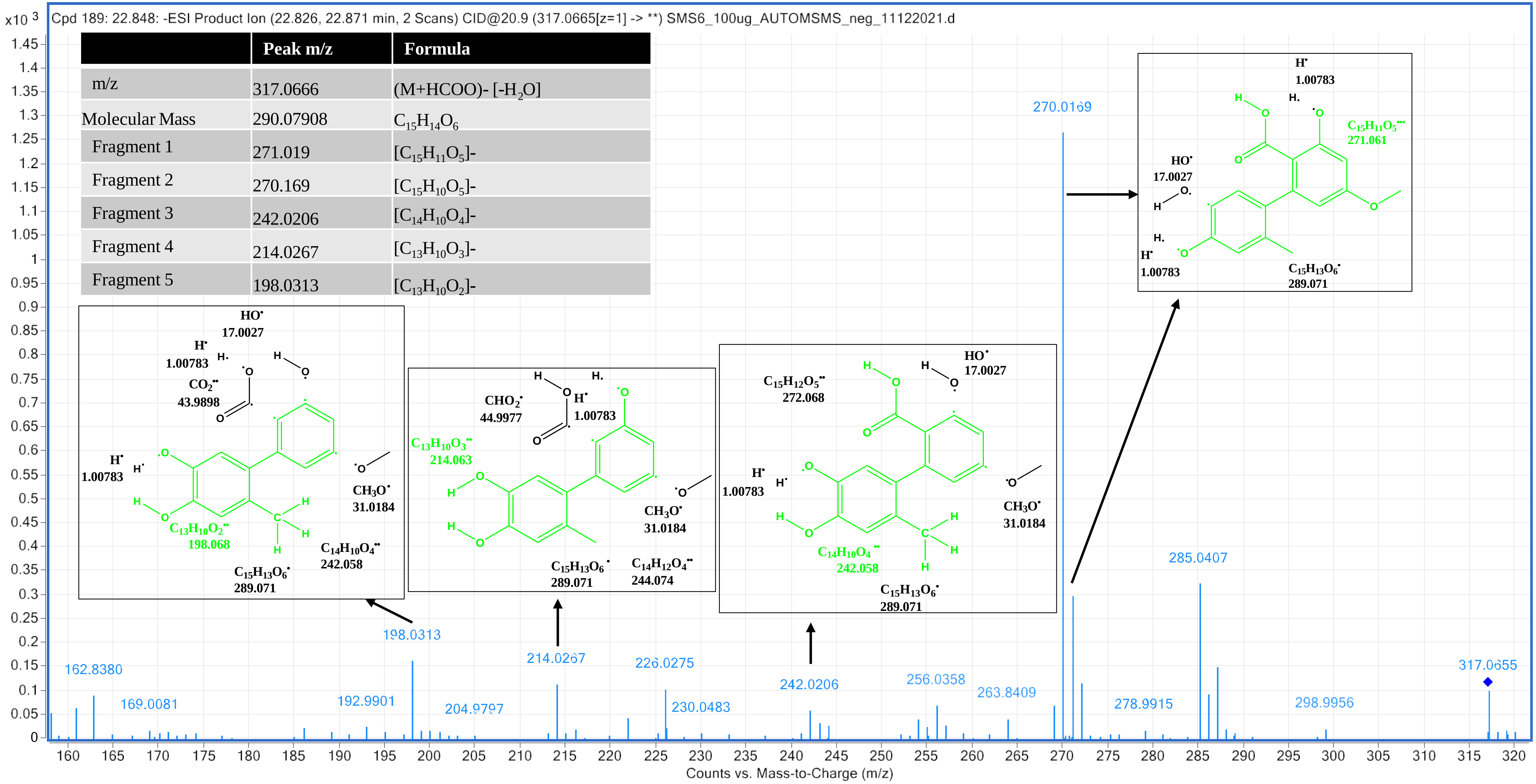

Supplement: Supplementary file 3 [file DataSheet3.pdf]
